# Evidence of disease severity, cognitive and physical outcomes of dance interventions for persons with Parkinson’s Disease: a systematic review and meta-analysis

**DOI:** 10.1186/s12877-021-02446-w

**Published:** 2021-09-22

**Authors:** Sophia Rasheeqa Ismail, Shaun Wen Huey Lee, Dafna Merom, Puteri Sofia Nadira Megat Kamaruddin, Min San Chong, Terence Ong, Nai Ming Lai

**Affiliations:** 1 Institute for Medical Research, National Institutes of Health, Ministry of Health, Shah Alam, Malaysia; 2 School of Pharmacy, Monash University Malaysia, Subang Jaya, Malaysia; 3 University of Western Sydney, Perth, Australia; 4 University of Malaya Medical Centre, Kuala Lumpur, Malaysia; 5 School of Medicine, Faculty of Health and Medical Sciences, Taylor’s University, Subang Jaya, Malaysia

## Abstract

**Background:**

Patients with Parkinson’s Disease (PD) usually experience worsening of both motor and non-motor symptoms. Dancing has been postulated to help patients with Parkinson’s via several mechanisms that lead to improved physical, cognitive and social functions.

**Methods:**

This systematic review was conducted following Cochrane methodology and reported following the PRISMA guideline. Four databases (up to June 2021) were searched for RCTs comparing dance to standard or other physical therapy for improvements in disease severity, quality of life, cognitive and physical outcomes as well as adverse events in patients with PD. We synthesised data using RevMan and included certainty-of-evidence rating (GRADE) for major outcomes.

**Results:**

A total of 20 RCTs (*N* = 723) articles that evaluated Tango, Ballroom, Irish, Waltz-Foxtrot, Folk, Turo, mixed dances and a PD-tailored dance were included. Dancers (versus non-dancers) had better motor experience (MDS-UPDRS 3) (MD -6.01, 95 % CI -9.97 to -3.84; *n* = 148; 5 RCTs) and improved balance (MiniBest Test) (MD 4.47, 95 % CI 2.29 to 6.66; n = 95; 3 RCTs), with no consistent differences on gait, agility and cognitive outcomes. Small samples and methodological limitations resulted in low-certainty-evidence across outcomes.

**Conclusions:**

Apart from a suggestion that dance intervention modestly reduced motor disease severity and improved certain aspects of balance, there is insufficient evidence on all other outcomes, such as agility and motor function, cognitive, mood and social outcomes, quality of life as well as adverse events including the risk of fall. As evidence is insufficient to inform practice, evidence of benefits on motor disease severity and balance needs to be considered in the context of user-perception of benefit versus harm and acceptability in the development of practice guideline recommendations.

**Supplementary Information:**

The online version contains supplementary material available at 10.1186/s12877-021-02446-w.

## Introduction

Parkinson’s disease (PD) is a progressive neurodegenerative condition characterised by loss of dopaminergic neurons in the substantia nigra leading to decreased levels of dopamine in the striatum [1]. With increased prevalence in older adults, PD affects 2 per 1000 people [2]. The loss of dopaminergic neurons in substantia nigra precipitates both motor and non-motor symptoms. People with PD typically have bradykinesia, rigidity, tremor and postural instability, while non-motor features includes cognitive impairment, mood disorders, fatigue, urinary and bowel symptoms [3]. Management is aimed at slowing the decline of motor and non-motor functions to improve quality of life mainly by pharmacological modalities with or without physical therapy. However, pharmacological treatment is accompanied by significant adverse effects and pill-burden [4].

Dance is characterised by the synchronization of movement to musical rhythms that integrates multiple physical, cognitive, emotional and social elements. The complex sensorimotor activities has been shown to activate the medial geniculate nucleus thereby potentially improving motor and non-motor features of the disease [5]. Mirroring, a technique used in dancing, activates the mirror neurons in the brain which would then lead to hormonal changes, positive effects on cognitive function, engagement of body memories for reminiscence therapy, and improvement in social cognitive theory constructs [6–11]. Consequently, positive group dynamics, mutual support and trust, corrective emotional experiences, empowerment, probing social roles, and enactive interpersonal learning experiences would be enhanced. Dance intervention focuses on maintaining posture, balance, movement and strength leading to improvements in gait, balance and reduce risk of fall as seen in healthy older adults [12]. Physical, psychological and social outcomes improvements were also seen in elderly people with dementia, depression and cancer [13–15]. Although the role of exercise in PD has been recognised in treatment guidelines, dance is less often highlighted therefore requires further exploration as compared to standard of care and other physical treatments.

Current trends suggest that the prevalence and burden of PD are expected to continuously increase [16] thereby prompting the search for stronger evidence for the management of people with PD. There are at least five recently published systematic reviews [17–21] and one narrative review [22] that evaluated the role of various forms dancing on various outcomes in PD. However, limitations of the aforementioned reviews included the comprehensiveness of the search, inclusion of non-randomised studies with inherent high risk-of-bias, rigour of methodology and the absence of rating of certainty of evidence which has become essential in the incorporation of evidence into guidelines. In this review, we aimed at evaluating the effectiveness and safety of dance as compared to standard therapy or to other physical intervention in improving disease severity, cognitive and physical outcomes as well as quality of life. We addressed the issues of previous reviews via an up-to-date systematic review of randomised controlled trials, with robust methodology that follows the Cochrane methods and with certainty of evidence rating using an established and widely used Grading of Recommendations Assessment, Development and Evaluation (GRADE) methodology [23]. In this review, we hypothesised that dancing interventions improve motor, cognitive and social outcomes, as well as quality of life for people with Parkinson’s disease.

## Methods

The systematic review is registered with PROSPERO (CRD42018081017). The registered protocol is available at https://www.crd.york.ac.uk/prospero/display_record.php?RecordID=81017.

### Types of studies

We included randomised-controlled trials and controlled trials with unclear allocation methods. We excluded trials that are clearly stated as non-randomised or quasi-experimental.

### Participants

All adults with Parkinson’s disease (PD), diagnosed via established clinical diagnostic criteria and/or neuroimaging and/or biomarker testing. We included studies that examine patients of all severities, stages and durations, and whether or not they are on stable therapy.

### Interventions

Any form of dancing, either recreational or therapeutic, that involves rhythmic movement of body in response to verbal, musical or other form of cues. Examples of well-recognised types of dance include Line-dancing, Ballet, Jazz, Hip-hop, Country, Western, Flamenco, Swing, Latin, Folk dance, Waltz, Tango, Cha-cha, Rhumba, Samba, Mambo, Quickstep, Jive and Zumba.

### Comparisons

We compared dance interventions with standard therapy but no dancing, or with other intervention in the form of physical or non-physical activities. We accepted studies that compare two types of dancing interventions.

### Outcome measures

#### Primary outcomes

Disease severity measured using established instruments such as MDS-UPDRS (total score of part I-IV) (total score 0-209, higher worse) [24], Webster Rating Scale (0–30, higher worse) [25] or other scale evaluating motor disturbance, and specific physical outcomes such as gait balance and range of motion.

#### Secondary outcomes

Cognitive outcomes (memory, processing time, execution and reaction time at so on), quality of life measured using the disease-tailored Quality of Life Scale (e.g. PDQ-39).

Key references to tools used in the evaluation of major outcomes in this review are reported in Additional File 1.

### Search strategies

We searched MEDLINE, EMBASE, Cochrane Central Register of Controlled Trials (CENTRAL) for published studies, and trial registries including the WHO International Trial Registry Platform and ClinicalTrials.gov for on-going studies, from inception up to 18 June 2021, without language restriction. We reviewed the references lists of retrieved articles for further trials for inclusion and contacted the authors of relevant trials to request details of any additional relevant published, unpublished or ongoing studies. Our search strategy for Medline is listed in Additional File 2. We adapted the same search strategy for other databases.

### Selection of studies, data extraction and management

We followed the Cochrane methods, as described in the *Cochrane Handbook for Systematic Reviews of Interventions* [26]. A detailed description of our methods is available in Additional File 3 and following is a summary.

Two authors independently screened the titles and/or abstracts for potentially eligible studies, and two authors then independently evaluated the abstract and full texts of the shortlisted articles to determine eligibility. We delineated the study selection process in a PRISMA diagram. Two review authors independently extracted and coded all data from each included study using a data collection form, including design, population, intervention, comparison and outcomes. Disagreements along these steps were resolved via discussion, with the input of a third author if necessary. One review author transferred the data to Review Manager 5.4 software [27], and a second review author checked the accuracy of data entry.

### Assessment of risk-of-bias

The Cochrane risk-of-bias tool for randomised trials was used for risk of bias assessment of all included studies [28]. Two review authors assessed each included trial for risk-of-bias independently according to the following six major criteria: sequence generation, allocation concealment, blinding of patient and personnel, blinding of outcome assessors, incomplete outcome data and selective outcome reporting. Each domain was judged either low, high or unclear risk of bias (Additional File 4).

### Assessment of heterogeneity, meta-analysis and certainty of evidence rating

We used the I^2^ statistic to quantify the degree of heterogeneity in the results [29], with a cut-off of 50 % and above considered as substantial heterogeneity. We pooled data and performed meta-analysis where appropriate, using a random-effect model in the RevMan 5.4 software [27]. We reported our results using mean difference (MD) and risk ratio (RR) for continuous and dichotomous outcomes respectively with their 95 % confidence intervals (CI). We performed certainty of evidence using the GRADE approach for all the outcomes and highlighted some major outcomes using one ‘Summary of findings’ table for each comparison (Additional File 5) [23]. Further details on our methodology, including how we addressed heterogeneity, undertook meta-analysis and performed GRADE certainty-of-evidence rating are available in Additional File 3.

We performed our meta-analysis by entering all data into the Review Manager software version 5.4, where the effect size calculations were automatically performed, using the inverse variance approach with the following core formula in calculating weighted average: $$Generic inverse-variance weighted average= \frac{\sum {Y}_{i}(1 /{S{E}_{i}}^{2}) }{\sum (1 /{S{E}_{i}}^{2})}$$, and with additional incorporation of the random effect model using DerSimonian and Laird method [30], which includes the following additional formula to calculate the prediction interval that takes into account of the variance between studies: $$M\pm {t}_{k-2} \times \sqrt{{Tau}^{2}+SE{\left(M\right)}^{2}}$$ [31].

## Results

**Fig. 1:**
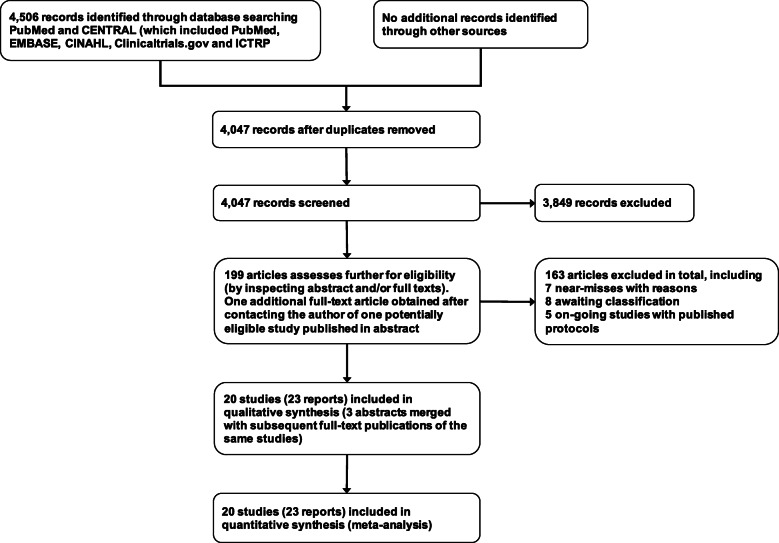
The PRISMA flow diagram of the study identification process from screening to analysis

The search yielded 4,506 records after removal of duplicates. We shortlisted 199 articles and included 20 reports, with eight studies awaiting full-texts and five on-going studies. There were 20 studies described in these 23 reports, as three pairs of reports described the same studies [32–37]. The PRISMA flow diagram of the study identification process is shown in Fig. 1.

Among the 20 included studies, 16 were parallel-group, individually randomised, two-arm RCTs. One study was a randomised, crossover trial [38], one study was a three-arm controlled trial with alternate allocation [37], and two more studies [39, 40] had unclear methods of allocation. Eleven studies were conducted in the United States of America (USA) [36, 37, 40–48], two each in the United Kingdom (UK) [34, 35] and Italy [49, 50], with one each in Ireland [51], Canada [52], Germany [53], Australia [54] and Republic of Korea [38]. The number of participants ranged from 10 [42] to 119 [37].

A list of the essential characteristics of each included study is included in Additional Table 1. A more detailed description of the characteristics of the included studies and their detailed risk-of-bias evaluation, as well as the characteristics of excluded and on-going studies are available in Additional File 4.

### Risk-of-bias in included studies

**Fig. 2:**
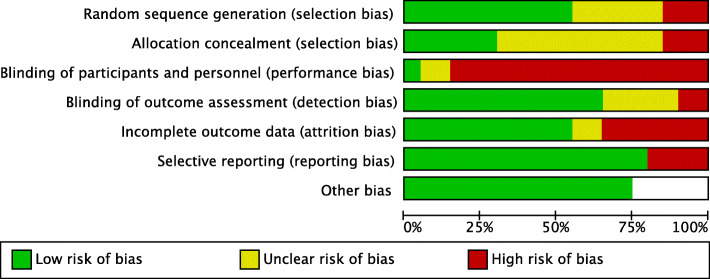
The risk-of-bias graph: proportions of studies with low, high, and unclear risks of bias in each domain

**Fig. 3:**
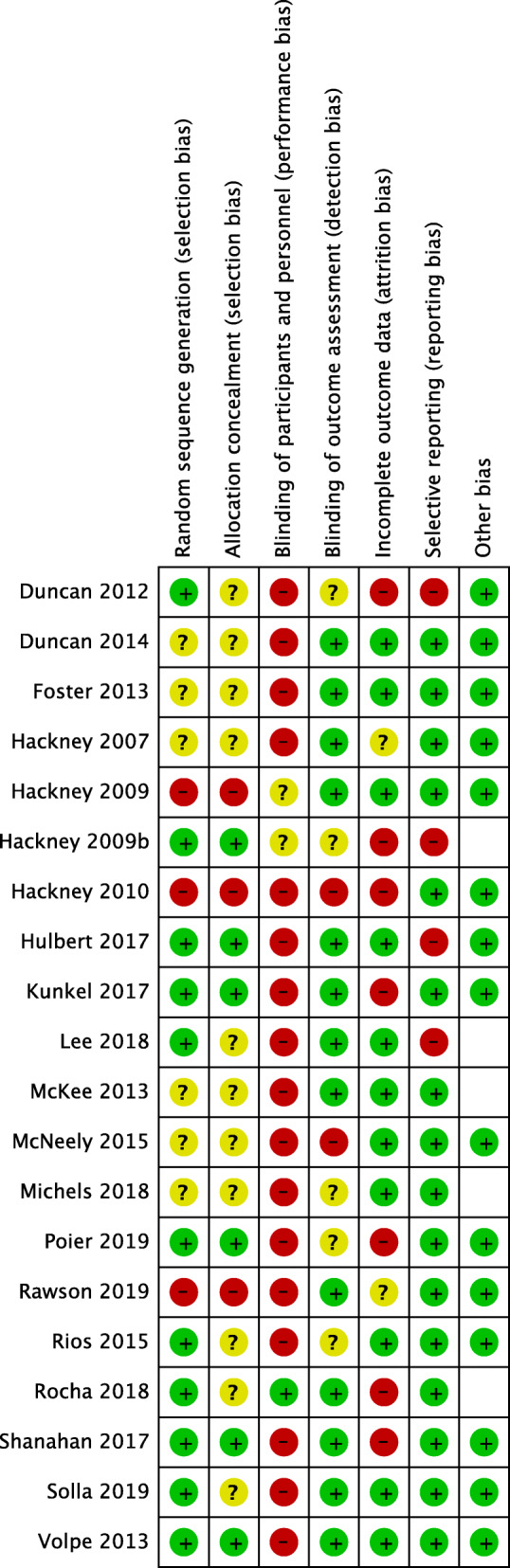
The risk-of-bias summary: the risk-of-bias judgement of each included study in each domain

The proportions of studies with low, high, and unclear risks-of-bias in each domain is illustrated in Fig. 2, and the risk-of-bias judgement of each included study in each domain is depicted in Fig. 3. Overall, there was a wide variation in the risks-of-bias of the studies across domains, with the majority judged to have low risk in blinding of outcome assessment, incomplete outcome data and selective reporting, while a minority having low risk in random sequence generation, allocation concealment and least of all, blinding of participants and personnel. A small but significant proportion of studies were judged to have high risk-of-bias in random sequence generation, allocation concealment, blinding of participants and personnel and incomplete outcome data. Meanwhile, a large proportion of included studies did not provide sufficient information to enable a meaningful risk-of-bias assessment. A detailed description of the risk-of-bias of each study is provided in Additional File 4.

### Effects estimates

In total, 18 studies with 723 participants contributed data, while the outcome data of two studies [34, 47] were not reported sufficiently for meta-analysis. Four major comparisons were evaluated, namely, (1) dancing in various forms versus no dancing; (2) two different forms of dance intervention: (i) specifically-tailored Dance for Parkinson’s Disease (D4PD) versus Tango [39], (ii) Tango versus mixed dances [54], and (iii) partnered versus non-partnered dance [45]; 3) Dance versus different exercises, as follows: i).Tango versus treadmill [37], ii).Tango versus stretching and flexibility exercises [37, 46] and iii) Tango versus Tai-Chi [53]; and 4) Dance versus physiotherapy [49].

Data from 4 studies were extrapolated from the graphs [37, 42, 43, 48]. The data from one study were reported in median and interquartile ranges [51], so they were unsuitable to be included in meta-analysis. The data were displayed in a separate table (Additional File 6).

See Summary of findings Tables 1, 2, 3, 4, 5, 6, 7 and 8 (Additional File 5) for certainty-of-evidence ratings for all outcomes along with reasons for downgrading. A list of all outcome estimates per comparison and their corresponding Forest plots are available in Additional File 7 and Additional File 8 respectively. Below is a summary of the major outcome estimates.

### Comparison 1: Dancing versus no dancing

There were ten studies that compared dance interventions with no dancing. Nine studies compared with usual care and one study compared with education [40]. The types of dances included Tango, Waltz, Foxtrot, ballroom, Latin American dances and Sardinian folk dances adapted to persons with PD. Usual care was mainly defined as the continuation of medications, medical appointments, and any existing usual exercise programs.

#### Primary outcomes

*1. Disease severity*.

a MDS-UPDRS subscales 1 (non-motor experiences of daily living, 0–16) and 2 (motor experiences of daily living, 0–56) (higher score indicates more severe disease).

Based on the same two studies (n = 23), dancers appeared to have lower but insignificant scores in both MDS-UPDRS subscale 1(MD -3.50, 95 % CI -18.68 to 11.67) and MDS-UPDRS subscale 2 (MD -2.09, 95 % CI -7.57 to 3.40) (low-certainty evidence, downgraded due to concerns on risk-of-bias and imprecision).

b. MDS-UPDRS 3 (Motor examination, 0-108) (higher score indicates a more severe disease).

At 3 months, our studies (n = 148) revealed.

**Fig. 4:**
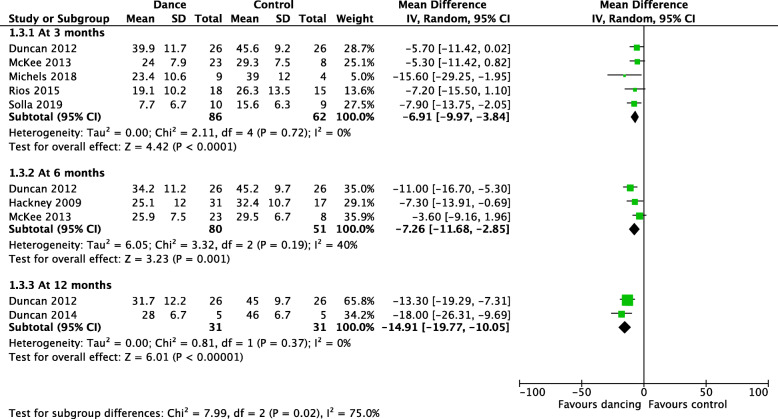
The Forest plot for one of the major outcomes: MDS-UPDRS 3 subscale (motor examination, 1-108, higher score indicates a more severe disease)

Based on five studies, dancers appeared to have lower scores in MDS-UPDRS subscale 3 at 3 months (MD -6.91, 95 % CI -9.97 to -3.84; n = 148; I^2^ = 0 %). Based on three studies, dancers also had lower MDS-UPDRS subscale 3 scores at both 6 months (MD -7.26, 95 % CI -11.68 to -2.85; n = 131; I^2^ = 0 %) and 12 months (MD -14.91, 95 % CI -19.77 to -10.05; n = 62; I^2^ = 0 %)(low-certainty evidence, downgraded due to concerns on risk-of-bias and imprecision) (Fig. 4).

c. MDS-UPDRS 4 (dyskinesia, 0–13, fluctuation, 0–10) (higher score indicates more severe disease).

Based on a single study (n = 33), there were no significant differences in the score in MDS-UPDRS 4 subscale between dancers and non-dancers (dyskinesia subscale: MD -0.10, 95 % CI -0.79 to 0.59; fluctuation subscale: MD 0.60, 95 % CI -0.92 to 2.12;) (low-certainty evidence, downgraded due to concerns on risk-of-bias and imprecision).

*2. Balance*.

a. Mini-BEST Test (0–28, higher score indicates better balance).

Based on three studies (n = 95), dancers may have better balance, assessed using Mini-BEST Test compared to non-dancers (MD 4.47, 95 % CI 2.29 to 6.66; I^2^ = 0 %) (moderate-certainty evidence, downgraded due to concern on risk-of-bias).

b. Berg Balance Scale (0–56, higher score indicates better balance).

Due to large degree of heterogeneity when the results of the five included studies were pooled, we separated the studies into subgroups based on the timing of outcome measurement (at 2, 3, 4 and 6 months), and found a difference in the scores of Berg’s Balance Scale at 3 months favouring dance (MD 8.42, 95 % CI 3.68 to 13.17; n = 32), but no significant differences at 2 months (MD 1.10, 95 %CI -0.81 to 3.01; n = 41), at 4 months (MD 5.10, 95 % CI -0.00 to 10.20; n = 48) and 6 months (MD -1.60, 95 % CI -4.54 to 1.34; n = 46) (low-certainty evidence, downgraded due to concerns on risk-of-bias and imprecision).

c. Activity-specific balance confidence (0-100, higher score indicates better balance confidence).

Based on one study (n = 46), it was unclear whether dancers had better balance confidence compared to non-dancers, as assessed using Activity-specific balance confidence scale (MD 0.20, 95 % CI -12.72 to 13.12) (Low-certainty evidence, downgraded due to concern on risk-of-bias and imprecision).

*3. Gait*.

a. Freezing of gait questionnaire (0–24, higher score indicates more severe condition).

Based on three studies (n = 89), dancers appeared to have lower questionnaire score (MD -0.39, 95 % CI -2.99 to 2.24; I^2^ = 0 %) (low-certainty evidence, downgraded due to concern on risk-of-bias and imprecision).

*4. Range or motion, speed, flexibility or agility*.

a. Timed Up and Go Test (TUG).

Seven studies measured agility using TUG (second). Dancers appeared to perform slightly better in TUG Test (MD -1.16, 95 % CI -2.17 to -0.15; participants = 200; studies = 7; I^2^ = 47 %) (low-certainty evidence, downgraded due to concern on risk-of-bias, inconsistency and imprecision).

There was substantial heterogeneity in the pooled estimate for this outcome, as shown by an I^2^ statistic of 47 %. We found that removing Kunkel 2017 [35] reduced I^2^ to 0 %, while removing one or more others did not reduce I^2^ substantially. We explored possible factors that could have accounted for the difference between Kunkel 2017 and other included studies, from study conduct (risk-of-bias) to characteristics of the population, intervention/comparison, outcome measurement, and risk-of-bias profile. We found a major difference between Kunkel 2017 [35] and other included studies, as the final outcome measurement took place at 6 months, well after the completion of the intervention which lasted for 10 weeks, while all other studies had their final outcome measured at or soon after the completion of the intervention. However, we still considered appropriate to pool the outcome estimates of the six studies in a meta-analysis, rather than placing Kunkel 2017 in a different subgroup.

b. Five Times Sit-to-Stand Test (FTSTT) (seconds, shorter better).

Based on a single study, dancers appeared to perform better in the FTSTT test (MD -4.90, 95 % CI -6.51 to -3.29; participants = 19) (low-certainty evidence, downgraded due to concerns on risk-of-bias and imprecision).

c. Sit-and-Reach Test (SRT) (cm, shorter distance better).

Based on a single study, there was no clear differences between groups in the performance in SRT (MD 4.60, 95 % CI -2.78 to 11.98; participants = 19) (low-certainty evidence, downgraded due to concerns on risk-of-bias and imprecision).

d. Walking distance: six-minute walk test (meters).

Due to large degree of heterogeneity, we separated the four included studies into two subgroups based on the type of dance offered. We found that among the three studies that evaluated Tango (n = 104), there were no significant difference between groups in walking distance in six minutes (MD -1.34, 95 % CI -53.91 to 51.24; I^2^ = 49 %), but in the single study that evaluated Sardinian folk dance, dancers appeared to perform better in the six-minute-walk-test (MD 238.80, 95 % CI 157.99 to 319.61; participants = 19) (low-certainty evidence, downgraded due to concern on risk-of-bias and imprecision).

e. Forward velocity (meters/second).

Based on one study (n = 48), there were no significant differences between groups in forward velocity (meters/second) (MD 0.10, 95 % CI -0.10 to 0.30) (low-certainty evidence, downgraded due to concern on risk-of-bias and imprecision).

f. Standing-start 180 degree turn test and spinal mouse inclination.

Based on one study (n = 46), there were no significant differences between groups in 180 degree turn test in the number of steps taken (MD 1.30, 95 % CI -0.38 to 2.98) and the time taken to complete the turn (MD 0.40, 95 % CI -0.18 to 0.98), as well as spinal mouse inclination degree (MD 0.80, 95 % CI -3.61 to 5.21) (low-certainty evidence, downgraded due to concern on risk-of-bias and imprecision).

g. Back-Scratch test.

A single study showed no clear difference between groups in the BST (MD 5.30, 95 % CI -2.94 to 13.54; participants = 19)) (low-certainty evidence, downgraded due to concern on risk-of-bias and imprecision).

h. Spinal mouse inclination degree.

A single study showed no clear difference between groups in the spinal mouse inclination degree (MD 0.80, 95 % CI -3.61 to 5.21; participants = 46) (low-certainty evidence, downgraded due to concern on risk-of-bias and imprecision).

4. Adverse events: falls during study

A single study showed no significant differences between groups in the risk of fall during the study period (RR 0.56, 95 % CI 0.11 to 2.90, participants = 33) (moderate-certainty evidence, downgraded due to concern on imprecision).

#### Secondary outcomes

There were no significant differences between groups in all secondary outcomes under this comparison, including cognitive function (Montreal Cognitive Assessment Scale), activity participation (Activity Card Sort), depressive symptoms (Beck Depression Inventory), apathy (Apathy Scale), fatigue (Krupp Fatigue Severity Scale) and quality of life (PDQ-39) (for detail please refer to Additional File 7) (low-certainty evidence for all outcome estimates, downgraded due to concern on risk-of-bias and imprecision) (Additional File 5).

### Comparison 2: Two different forms of dance interventions

Three included studies compared two different forms of dance intervention. One study compared a specific dance for PD (D4PD) and Tango [39], another study compared tango with mixed dances [54], and one study compared partnered dancing with non-partnered dancing [45].

### Dance for Parkinson’s disease (D4PD) versus Tango

There were no significant differences between groups in all outcomes estimates under this comparison, including disease severity (MDS-UPDRS subscale 3), Timed Up and Go Test, six-minute walk test, forward velocity and quality of life (for details please refer to Additional File 7). The certainty of evidence is low for all outcomes included in this comparison, due to serious concerns on the risk-of-bias of the included study and imprecision (Additional File 5).

### Tango versus mixed dances

There were no significant differences between groups in all outcomes estimates under this comparison, including Timed Up and Go Test, Functional Gait Assessment, Freezing Gait Questionnaire, Berg Balance Scale, disease severity (MDS-UPDRS 2 & 3), and quality of life (PDQ39) (for details please refer to Additional File 7). The certainty of evidence is low for all outcomes included in this comparison, due to serious concerns on the risk-of-bias of the included study and imprecision (Additional File 5).

### Partnered vs. non-partnered dance

Based on one study in this comparison (n = 39), there were no significant differences between groups in balance (Berg Balance Scale) as well as Timed Up and Go Test (for details please refer to Additional File 7). The certainty of evidence is very low for both outcomes, due to very serious concerns on risk-of-bias of the included study (downgraded two levels) and imprecision (downgraded one level) (Additional File 5).

### Comparison 3: Dancing versus different exercises

There were three comparisons made under this category: i).Tango versus treadmill [37], ii).Tango versus stretching and flexibility exercises [37, 46] and iii) Tango versus Tai-Chi [53].

### Tango versus treadmill

There were no significant differences between groups in all outcomes estimates under this comparison, including disease severity (MDS-UPDRS subscale 3), balance (Mini Best Test), six-minute walk test, forward velocity and quality of life (PDQ-39) (for details please refer to Additional File 7). The certainty of evidence is very low for all outcomes included in this comparison, due to very serious concerns on the risk-of-bias of the included study (downgraded two levels) and serious concern on imprecision (downgraded one level) (Additional File 5).

### Tango versus stretching or flexibility exercises

There were no significant differences between groups in all outcomes estimates under this comparison, including disease severity (MDS-UPDRS subscale 3), balance (Mini Best Test and Berg Balance Scale), Freezing of gait questionnaire score, six-minute walk test, forward velocity and quality of life (PDQ-39) (for details please refer to Additional File 7). The certainty of evidence is low to very low for all outcomes included in this comparison, due to either serious or very serious concerns on the risk-of-bias of the included study, which resulted in a downgrade of one or two levels respectively, and imprecision (downgraded one level) (Additional File 5).

### Tango versus Tai-Chi

Based on a single study (n = 29), there were no significant differences between groups in quality of life (PDQ-39) and Life satisfaction (Brief Multidimensional Life Satisfaction Scale (BMLSS) scores. The certainty of evidence is low for both outcomes due to serious concerns on the risk-of-bias and imprecision (downgraded one level) (Additional File 5).

### Comparison 4: Dance versus physiotherapy

#### Irish Dance vs. physiotherapy

Based on one study in this comparison (n = 24), dancers appeared to have lower severity of disease in the motor examination subscale of the MDS-UPDRS subscale 3 (MD -3.60, 95 % CI -6.42 to -0.78), improved balance as measured by the Berg Balance Scale (MD 7.20, 95 % CI 0.36 to 14.04) and better ratings in the Freezing of Gait Questionnaire (MD -5.30, 95 % CI -8.11 to -2.49). However, there were no significant differences in the risks of fall of any cause between groups either during the time of intervention or during the entire study period (during the time of intervention: RR 3.00, 95 % CI 0.13 to 67.06; during the entire study: RR 0.90, 95 % CI 0.60 to 1.36), as well as the quality of life (PDQ-39) (MD -5.40, 95 % CI -12.63 to 1.83) (for details please refer to Additional File 7).

The certainty of evidence is low for all outcomes included in this comparison, due to serious concerns on the risk-of-bias of the included study and imprecision (downgraded one level each) (Additional File 5).

## Discussion

Based on limited evidence, this review highlights the modest improvement of motor function, non-motor function, and balance in people with Parkinson’s disease who underwent dance intervention compared to usual care. Irish dance intervention also offered modest improvement in motor function, balance and gait, as compared to physiotherapy. There were no clear differences between groups in most other outcomes evaluated in the meta-analysis. The available evidence was limited by the small samples and serious concerns on the risk of bias of the included studies.

The overall findings of this review were consistent with the findings of other reviews [17, 18, 20, 21]. Based on five RCTs (159 participants), Dos 2018 [17] concluded that dance conferred significant improvements in disease severity as measured using MDS-UPDRS III, as well as reaction time measured using TUG test, compared to other types of exercise. All five RCTs included in Dos 2018 [17] were included in our review. Even with the inclusion of mixture of RCTs and non-RCTs as in Lötzke 2015 [18] and Kalyani 2019 [20], motor and cognitive improvements were similar. Both Lötzke 2015 [18] and Kalyani 2019 [20] concluded that there were moderate motor and possibly cognitive benefits in favour of Argentine Tango specifically [18] and dance in general [20], although the certainty of evidence was unclear.

Cognitive assessment was evaluated in two studies that compared dance with usual care or education and showed small, insignificant cognitive improvements. The review by Zhang 2019 [19] evaluated mainly cognitive and mood symptoms and included seven RCTs (185 participants). While only two of the seven RCTs included in Zhang 2019 [19] was included in our review, due possibly to different scope of search, the authors concluded that dance improved executive function but did not seem to improve global cognitive and mood outcomes. The lack of cognitive assessment and the resulting inconsistent results as compared to sensorimotor assessment was highlighted in a review by Bek 2020 [22].

### Strengths and Limitations

We believe we have captured most literature that are relevant to this review through our comprehensive search strategy from multiple databases with independent screening, selection, and assessment of eligible studies. However, future inclusion of the eight on-going studies may change some findings due to the small number of studies and participants in most outcomes of this review. Additionally, our review has the advantage of incorporating the certainty of evidence rating, which was not included in the aforementioned reviews. Of the five similar reviews on dance interventions and Parkinson’s disease, Carapellotti 2020 [21] was recently published and included the most articles thus far. However, four RCTs were missed, strength of evidence was not included, and intervention comparisons were only grouped into dance versus no or active intervention only. The lack of certainty of evidence rating might lead to inappropriate confidence in the findings, as possibly reflected in the reviews above in the tone of their conclusions about the effects or lack thereof of dance therapy.

Apart from two studies, the rest of the included studies were conducted in Europe or North America, thereby limiting the generalisability of the findings. Findings were also limited due to the very low to low certainty of evidence in almost all outcomes which were contributed by serious concerns of risk-of-bias, imprecision of the outcome estimates and inconsistency of the estimates. Serious concerns were made for performance bias, selection bias, and attrition bias domains. As many outcomes such as disease severity, balance and quality of life were subjective outcomes, the lack of blinding of participants and care personnel in all studies could have had potential impact on outcome performance. Although participant blinding would be impossible, blinding of outcome assessor would have been possible and would have limited this concern. Serious concerns on attrition bias were due to the high withdrawal rates in individual studies (15–54 %) and reasons for withdrawal were likely outcome-related thereby causing under- or overestimation of reported outcomes. Imprecision of outcome estimates, as reflected by wide confidence intervals, was due to the small sample sizes in single studies which were underpowered to detect important differences in the effect estimates.

## Conclusions

Although certainty of evidence was low, our review suggests that dance intervention modestly reduced motor disease severity and improved certain aspects of balance, while there is insufficient evidence on all other outcomes. The wide variety of dance intervention types and outcome assessed diluted the strength of the evidence base on the effectiveness of dance in people with PD. Evidence of benefits on motor disease severity and balance needs to be considered in the context of user-perception of benefit versus harm and acceptability in the development of practice guideline recommendations. Given the difficulties in achieving blinding of participants and personnel, it is essential that future RCTs adhere to rigorous standards in random sequence generation, allocation concealment and blinding of outcome assessor, with clear documentation, to offer any improvement in the overall certainty of evidence.

## Supplementary Information

**Additional file 1.** Key references to tools used in the evaluation of major outcomes.

**Additional file 2. **MEDLINE (PubMed) search strategy.

**Additional file 3. **Additional details on the methodology.

**Additional file 4. **Characteristics of studies in greater details.

**Additional file 5. **Summary of findings tables with certainty of evidence rating.

**Additional file 6. **Outcome data for the study with skewed data.

**Additional file 7. **Summary of all outcome estimates.

**Additional file 8.** Forest plots.

**Additional file 9. **Completed PRISMA checklist for reporting of systematic review.

**Additional file 10: Table S1**. Characteristics of included studies

## Data Availability

The dataset used and analysed during this review are available from the corresponding author on reasonable request.
