# Supplementary material for: Evidence of disease severity, cognitive and physical outcomes of dance interventions for persons with Parkinson’s Disease: a systematic review and meta-analysis
Source: BMC Geriatr. 2021 Sep 22;21:503. doi: 10.1186/s12877-021-02446-w (PMC8456607; doi:10.1186/s12877-021-02446-w)
Supplement: Supplementary file 1 — Additional file 1. Key references to tools used in the evaluation of major outcomes. [file 12877_2021_2446_MOESM1_ESM.pdf]

Evidence of disease severity, cognitive and physical outcomes of dance interventions for persons with Parkinson's Disease: a systematic review and meta-analysis

Sophia Rasheeqa Ismail[1*], Shaun Wen Huey Lee[2], Dafna Merom[3], Puteri Sofia Nadira Megat Kamaruddin[1], Min San Chong[4], Terence Ong[4], Nai Ming Lai[2,5]

[1] Institute for Medical Research, National Institutes of Health, Ministry of Health, Malaysia

[2] School of Pharmacy, Monash University Malaysia, Malaysia

[3] University of Western Sydney, Australia

[4] University of Malaya Medical Centre, Malaysia.

[5] School of Medicine, Faculty of Health and Medical Sciences, Taylor's University Malaysia

Additional File 1

Key references to tools used in the evaluation of major outcomes in the review

**Outcome**	**Tool**	**Citation of key reference**
Disease severity (motor, behaviour, mentation, mood, activities of daily living)	MDS- Unified Parkinson's Disease Rating Scale (MDS-UPDRS)	Goetz CG, Tilley BC, Shaftman SR, Stebbins GT, Fahn S, Martinez-Martin P, Poewe W, Sampaio C, Stern MB, Dodel R *et al*: **Movement Disorder Society-sponsored revision of the Unified Parkinson's Disease Rating Scale (MDS-UPDRS): scale presentation and clinimetric testing results**. *Movement disorders : official journal of the Movement Disorder Society* 2008, **23**(15):2129-2170.
Balance	Mini Best Test	King L, Horak F: **On the Mini-BESTest: Scoring and the Reporting of Total Scores**. *Physical Therapy* 2013, **93**(4):571-575.
	Berg's Balance Scale	Berg K, Wood-Dauphinee S, Williams JI: **The Balance Scale: reliability assessment with elderly residents and patients with an acute stroke**. *Scandinavian journal of rehabilitation medicine* 1995, **27**(1):27-36.
	Spinal-mouse inclination score	S Keller, AF Mannion, Grob D: **Reliability of a new measuring device („spinalmouse") in recording the sagittal profile of the back**. *Eur Spine J* 2000, **9**(4):283-313.
	Activities-specific Balance Confidence Scale (ABC)	Powell LE, Myers AM: **The Activities-specific Balance Confidence (ABC) Scale**. *The journals of gerontology Series A, Biological sciences and medical sciences* 1995, **50a**(1):M28-34.
Gait and mobility	Freezing of Gait Questionnaire	Giladi N, Tal J, Azulay T, Rascol O, Brooks DJ, Melamed E, Oertel W, Poewe WH, Stocchi F, Tolosa E: **Validation of the freezing of gait questionnaire in patients with Parkinson's disease**. *Movement disorders : official journal of the Movement Disorder Society* 2009, **24**(5):655-661.
	Six-minute Walk Test	Garber CE, Friedman JH: **Effects of fatigue on physical activity and function in patients with Parkinson's disease**. *Neurology* 2003, **60**(7):1119-1124.
	Standing-start 180° turn test	Stack E, Ashburn A: **Dysfunctional turning in Parkinson's disease**. *Disability and Rehabilitation* 2008, **30**(16):1222-1229.
	Time-up-and-go (TUG) test	Podsiadlo D, Richardson S: **The timed "Up & Go": a test of basic functional mobility for frail elderly persons**. *Journal of the American Geriatrics Society* 1991, **39**(2):142-148.
	Five Times Sit-to-Stand Test	Duncan RP, Leddy AL, Earhart GM: **Five times sit-to-stand test performance in Parkinson's disease**. *Archives of physical medicine and rehabilitation* 2011, **92**(9):1431-1436.
	Sit-and-Reach Test	Bozic PR, Pazin NR, Berjan BB, Planic NM, Cuk ID: **Evaluation of the field tests of flexibility of the lower extremity: reliability and the concurrent and factorial**

		**validity**. *Journal of strength and conditioning research* 2010, **24**(9):2523-2531.
	Back Scratch Test	Roberta ER, Jones CJ: **Development and Validation of a Functional Fitness Test for Community-Residing Older Adults**. *Journal of Aging and Physical Activity* 1999, **7**(2):129-161.
	Nine-hole peg test	Earhart GM, Cavanaugh JT, Ellis T, Ford MP, Foreman KB, Dibble L: **The 9-hole PEG test of upper extremity function: average values, test-retest reliability, and factors contributing to performance in people with Parkinson disease**. *Journal of neurologic physical therapy : JNPT* 2011, **35**(4):157-163.
	Purdue pegboard	Desrosiers J, Hébert R, Bravo G, Dutil E: **The Purdue Pegboard Test: normative data for people aged 60 and over**. *Disabil Rehabil* 1995, **17**(5):217-224.
Risk of fall	Falls questionnaire (Canadian Community Health Survey (CCHS)— Healthy Aging (May, 2010) adapted to focus on the past 3 months.	https://www.statcan.gc.ca/eng/statistical-programs/document/5146_D1_T1_V1-eng.pdf
Cognitive/Mood outcomes	Montreal Cognitive Assessment (MoCA)	Nazem S, Siderowf AD, Duda JE, Have TT, Colcher A, Horn SS, Moberg PJ, Wilkinson JR, Hurtig HI, Stern MB *et al*: **Montreal cognitive assessment performance in patients with Parkinson's disease with "normal" global cognition according to mini-mental state examination score**. *Journal of the American Geriatrics Society* 2009, **57**(2):304-308.
	Depression: Beck's Depression Inventory	Beck AT, Ward CH, Mendelson M, Mock J, Erbaugh J: **An inventory for measuring depression**. *Archives of general psychiatry* 1961, **4**:561-571.
	Apathy: Apathy Scale	Starkstein SE, Mayberg HS, Preziosi TJ, Andrezejewski P, Leiguarda R, Robinson RG: **Reliability, validity, and clinical correlates of apathy in Parkinson's disease**. *The Journal of neuropsychiatry and clinical neurosciences* 1992, **4**(2):134-139.
	Fatigue: Krupp Fatigue Severity Scale	Alves G, Wentzel-Larsen T, Larsen JP: **Is fatigue an independent and persistent symptom in patients with Parkinson disease?** *Neurology* 2004, **63**(10):1908-1911.
Participation in activities of daily living	Activity Card Sort	Baum C, Edwards D: **Activity Card Sort (ACS)**. Bethesda, MD: AOTA Press 2008.
Quality of life	Parkinson's Disease Questionnaire - 39 (PDQ-39)	Peto V, Jenkinson C, Fitzpatrick R: PDQ-39: a review of the development, validation and application of a Parkinson's disease quality of life questionnaire and its associated measures. Journal of neurology 1998, 245 Suppl 1:S10-14.

	Brief Multidimensional Life Satisfaction Scale (BMLSS)	Büssing A, Fischer J, Haller A, Heusser P, Ostermann T, Matthiessen PF: **Validation of the brief multidimensional life satisfaction scale in patients with chronic diseases**. *Eur J Med Res* 2009, **14**(4):171-177.
