# Supplementary material for: Evidence of disease severity, cognitive and physical outcomes of dance interventions for persons with Parkinson’s Disease: a systematic review and meta-analysis
Source: BMC Geriatr. 2021 Sep 22;21:503. doi: 10.1186/s12877-021-02446-w (PMC8456607; doi:10.1186/s12877-021-02446-w)
Supplement: Supplementary file 2 — Additional file 2. MEDLINE (PubMed) search strategy. [file 12877_2021_2446_MOESM2_ESM.pdf]

Evidence of disease severity, cognitive and physical outcomes of dance interventions for persons with Parkinson's Disease: a systematic review and meta-analysis

Sophia Rasheeqa Ismail[1*], Shaun Wen Huey Lee[2], Dafna Merom[3], Puteri Sofia Nadira Megat Kamaruddin[1], Min San Chong[4], Terence Ong[4], Nai Ming Lai[2,5]

[1] Institute for Medical Research, National Institutes of Health, Ministry of Health, Malaysia

[2] School of Pharmacy, Monash University Malaysia, Malaysia

[3] University of Western Sydney, Australia

[4] University of Malaya Medical Centre, Malaysia.

[5] School of Medicine, Faculty of Health and Medical Sciences, Taylor's University Malaysia

Additional File 2

MEDLINE (PubMed) search strategy

The same search strategy was adapted in the searches of other databases

#1 danc*[Title/Abstract]

#2 dance therapy[MeSH Terms]

#3 dancing[MeSH Terms]

#4 movement* [Title/Abstract]

#5 exercise movement techniques[MeSH Terms]

#6 Tango [Title/Abstract]

#7 Ballroom [Title/Abstract]

#8 Foxtrot [Title/Abstract]

#9 Ballet [Title/Abstract]

#10 Waltz [Title/Abstract]

#11 Cha-Cha [Title/Abstract]

#12 "Cha Cha" [Title/Abstract]

#13 Rumba [Title/Abstract]

#14 Samba [Title/Abstract]

#15 Mambo [Title/Abstract]

#16 Quickstep [Title/Abstract]

#17 Jive [Title/Abstract]

#18 Salsa [Title/Abstract]

#19 Flamenco [Title/Abstract]

#20 Lambada [Title/Abstract]

#21 Polka [Title/Abstract]

#22 Swing [Title/Abstract]

#23 Jazz [Title/Abstract]

#24 #1 OR #2 OR #3 OR #4 OR #5 OR #6 Or #7 OR #8 OR #9 OR #10 OR #11 OR #12 OR #13 OR #14 OR #15 OR #16 OR #17 OR #18 OR #19 OR #20 OR #21 OR #22 OR #23

#25 parkinson's disease[MeSH Terms]

#26 parkinson*[Title/Abstract]

#27 paralysis agitans[Title/Abstract]

#28 paralysis agitans[MeSH Terms]

#29 #25 OR #26 OR #27 OR #28

#30 #24 AND #29

#31 "clinical study"[Publication Type]

#32 #30 AND #31
