# Supplementary material for: Evidence of disease severity, cognitive and physical outcomes of dance interventions for persons with Parkinson’s Disease: a systematic review and meta-analysis
Source: BMC Geriatr. 2021 Sep 22;21:503. doi: 10.1186/s12877-021-02446-w (PMC8456607; doi:10.1186/s12877-021-02446-w)
Supplement: Supplementary file 3 — Additional file 3. Additional details on the methodology. [file 12877_2021_2446_MOESM3_ESM.pdf]

Evidence of disease severity, cognitive and physical outcomes of dance interventions for persons with Parkinson’s Disease: a systematic review and meta-analysis

Sophia Rasheeqa Ismail[1*], Shaun Wen Huey Lee[2], Dafna Merom[3], Puteri Sofia Nadira Megat Kamaruddin[1], Min San Chong[4], Terence Ong[4], Nai Ming Lai[2,5]

[1] Institute for Medical Research, National Institutes of Health, Ministry of Health, Malaysia

[2] School of Pharmacy, Monash University Malaysia, Malaysia

[3] University of Western Sydney, Australia

[4] University of Malaya Medical Centre, Malaysia.

[5] School of Medicine, Faculty of Health and Medical Sciences, Taylor's University Malaysia

Additional File 3

Additional details on the methodology

Data extraction and management

Two review authors (SRI and MSC) independently extracted and coded all data from each included study using a dedicated data collection form, after an assessment of its usability via a round of piloting on three included studies. We collected study characteristics, including study design, setting, country, methods of allocation, participants, interventions, comparators, outcomes, sponsorship details, declaration of interests of the primary investigators, methods used to control possible conflicts of interests, and other information considered relevant according to Section 7.3 of the *Cochrane Handbook for Systematic Reviews of Interventions*[1]. We resolved potential discrepancies through discussion and involved a third review author if necessary.

We extracted the outcome data using an electronic data collection form. For continuous data, we extracted the mean value of the outcome measurement in each group at each time point (or, if this was unavailable, the mean change from baseline), the standard deviation (SD) values, and the number of participants used to measure the outcome for each group. For dichotomous outcomes, we extracted the number of participants in each outcome group at each time point. We contacted the study authors to obtain important missing data. If the study report only provided the summary effect sizes (e.g. risk ratio (RR) for dichotomous data and mean difference (MD) or standardised mean difference (SMD) for continuous data), we extracted those measures as well as the accompanying standard errors (SE) or 95% confidence intervals (CI) to prepare the data for combination via the generic inverse variance method. For studies that provided the outcome data in graphs without accompanying annotation or numerical report, we estimated the data from the graphs manually. Once the data was collected, one review author (NML) transferred the data to Review Manager 5 software [2], and a second review author (SRI) checked the accuracy of data entry.

Assessment of risk of bias in included studies

Two review authors (SRI, NML) assessed each included trial for risk of bias independently according to the following six major criteria, as recommended in the Cochrane Handbook for Systematic Reviews of Interventions: Sequence generation, allocation concealment, blinding of patient and personnel, blinding of outcome assessors, incomplete outcome data and selective outcome reporting. We assigned a judgment of either 'low', 'high' or 'unclear' risk with justifications on each criterion by completing a 'Risk of bias' table for each included trial. We discussed any disagreement among the review authors and would have involved a third review author if necessary (this was not needed).

Measures of treatment effect

We reported the pooled outcome estimates for continuous data using mean difference (MDs) if all data were of the same measurement scale, and for categorical data, we used risk ratio (RRs). We reported the point estimates with their respective 95% confidence intervals (CI). If pooled analyses were not possible due to reasons such as major discrepancies in study characteristics or outcome reporting, as detailed under the Assessment of heterogeneity section, we reported the results of the studies individually or in separate subgroup without combining the subgroup estimates.

Dealing with missing data

We followed the recommendations in Chapter 8.13.2 in the *Cochrane Handbook for Systematic Reviews of Intervention* in assessing the risk of bias from incomplete outcome data [3].

We performed our analyses for all outcomes, where possible, using intention-to-treat (ITT) data (analysed according to randomisation, irrespective of subsequent discontinuation of the study or deviation from the protocol, if the outcome data of these participants were available or were imputed by the study authors). If there were missing outcome data that were not imputed, we would have performed a modified ITT analysis (analysed according to randomisation with only available outcome data and without the missing data) [3]. If ITT data were not provided, we included outcome data of the participants either in a 'per protocol' or 'as treated' manner, as provided by the study authors, but made a corresponding note in the Characteristics of included studies table.

Assessment of heterogeneity

We used the I^2 statistic to quantify the degree of inconsistency in the results [4], with a cut-off of 50% and above considered as the level at which the degree of heterogeneity was of sufficient concern to justify an exploration of possible explanations. In such a situation, we evaluated studies in terms of their clinical and methodological characteristics using the following criteria to determine whether the degree of heterogeneity may be explained by differences in those characteristics, and whether a meta-analysis was appropriate.

We assessed the following criteria.

- Characteristics of the participants (e.g. age, type, and mode of diagnosis of Parkinson's disease).
- Settings of the studies (e.g. community or institution).
- Interventions (type of dance, dosage (intensity or duration of therapy)).
- Risk of bias (as detailed in the Assessment of risk of bias in included studies section).

If we identified any of the above-mentioned factors during our exploration that we considered to be a plausible explanation of the observed heterogeneity, we separated the studies into subgroups according to the factors concerned if there were sufficient studies in each subgroup. In the case of risk of bias, we conducted sensitivity analyses excluding the studies at higher risk of bias.

Assessment of reporting biases

We planned to use a funnel plot and Egger's test to screen for publication bias if there were at least 10 studies included in the analysis of the relevant outcomes [5]. If publication bias was suggested by significant asymmetry of the funnel plot, we would have included a statement in our results with a corresponding note of caution in our discussion, bearing in mind that funnel plot asymmetry does not necessarily equate to the presence of publication bias. If possible, we would have compared conference abstracts and available trial protocols of included studies with published data.

Data synthesis

We pooled the study data and perform meta-analysis if more than one study reported the same outcome and if the included studies were sufficiently homogenous in terms of population, intervention, comparison and outcome measured. We applied random effect model in our meta-analysis using the RevMan 5.3 software [2].
However, if there were marked differences between the study characteristics and reported outcomes, we would have summarised the results of the study narratively.

Where there were more than one group of dance intervention, for example, in [6], two groups of participants were allocated to Waltz/Foxtrot and Tango respective with a third group serving as control, we combined the estimates of the two intervention groups (mean, SD, number of participants) into a single group using the formula as recommended in Table 7.7a, Chapter 7 of the *Cochrane Handbook of Intervention* [1].

Subgroup analysis and investigation of heterogeneity

If sufficient data that were relevant were available, we would have performed subgroup analyses as follows based on the participant characteristics including gender (men, women or other) and age (under 65 years old; 65 years old and over), setting (community or institution), severity of Parkinson's disease, type of dance; intensity and length of intervention (intensity: weekly or less frequent vs twice weekly or more frequent; one hour or less per session vs more than one hour; length of intervention: shorter than 3 months vs longer).

Summary of findings' table

We performed certainty of evidence using the GRADE approach for all the outcomes included in this review, and highlighted some major outcomes using one 'Summary of findings' table for each comparison. We used the five GRADE criteria (study limitations, consistency of effect, imprecision, indirectness, and publication bias) to assess the certainty of the evidence for each of these outcomes based on the body of evidence generated by the studies that contributed data to the meta-analyses [7].

Specifically, for the criterion of study limitations, we made the decision on the overall risk of bias across the pool of relevant studies that contributed to each specific outcome rated on two levels: 1. determining the overall risk of bias of any single study, and 2. determining the risk of bias across the pool of relevant studies (namely, overall study limitation). To determine the overall risk of bias of any single study, we assigned the overall risk of bias status of the single study according to the worst risk of bias domain that was relevant to the specific outcome, apart from the domain of selective outcome reporting. To determine the risk of bias across the pool of relevant studies, we referred to the guideline as detailed in Table 12.2.d of the *Cochrane Handbook for Systematic Reviews of Intervention* [7].

If we identified an issue in any of the five GRADE criteria that we considered to pose a serious enough risk to influence the outcome estimate, we downgraded the certainty of evidence by one level, and when we considered the issue to be very serious, we downgraded the certainty of evidence by two levels [7]. Whenever we decided to downgrade the certainty of evidence from the default high certainty, we justified our decision and described the level of downgrading in the footnotes of the table. We constructed the 'Summary of findings' table using an Internet-based version of GRADEpro software [8], according to the methods and recommendations described in Chapter 11 of the *Cochrane Handbook for Systematic Reviews of Interventions* [9].

Sensitivity analysis

We planned to perform sensitivity analysis by evaluating the results with and without the inclusion of trials with high risk of selection and attrition biases, if we had sufficient number of studies. However, due to insufficient data, we did not perform any sensitivity analysis.

References

1. Higgins JP, Altman DG, Sterne JA. Chapter 7: Selecting studies and collecting data. In: Higgins JP, Green S, editor(s). Cochrane Handbook for Systematic Reviews of Interventions Version 5.1.0 (updated March 2011). The Cochrane Collaboration, 2011. Available from handbookcochraneorg.
2. Review Manager 5 (RevMan 5) [program]. Copenhagen: Nordic Cochrane Centre, The Cochrane Collaboration, 2014.
3. Higgins JP, Savovic J, Page MJ, et al. Chapter 8: Assessing risk of bias in a randomised trial. In: Higgins JP, Thomas J, Chandler J, Cumpston M, Li T, Page MJ, et al (editor(s). Cochrane Handbook for Systematic Reviews of Interventions Version 6.0 (updated July 2019). Cochrane, 2019. Available from wwwtrainingcochraneorg/handbook.
4. Higgins JP, Altman DG, Sterne JA. Chapter 9: Analysing data and undertaking meta-analysis. In: Higgins JP, Green S, editor(s). Cochrane Handbook for Systematic Reviews of Interventions Version 5.1.0 (updated March 2011). The Cochrane Collaboration, 2011. Available from handbookcochraneorg.
5. Egger M, Davey Smith G, Schneider M, et al. Bias in meta-analysis detected by a simple, graphical test. *BMJ* 1997;315(7109):629-34.
6. Hackney M E, Earhart G M. Effects of dance on movement control in Parkinson's disease: A comparison of Argentine tango and American ballroom. *Journal of Rehabilitative Medicine* 2009;41(6):475-81.
7. Schünemann HJ, Oxman AJ, Vist GE, et al. Chapter 12: Interpreting results and drawing conclusions. In: Higgins JP, Green S, editor(s). Cochrane Handbook for Systematic Reviews of Interventions Version 5.1.0 (updated March 2011). The Cochrane Collaboration, 2011. Available from handbookcochraneorg.
8. GRADEpro GDT [program]. Hamilton (ON): McMaster University (developed by Evidence Prime), 2015.
9. Higgins JP, Altman DG, Sterne JA. Chapter 11: Presenting results and "Summary of findings" table. In: Higgins JP, Green S, editor(s). Cochrane Handbook for Systematic Reviews of Interventions Version 5.1.0 (updated March 2011). The Cochrane Collaboration, 2011. Available from handbookcochraneorg.
