# Supplementary material for: Evidence of disease severity, cognitive and physical outcomes of dance interventions for persons with Parkinson’s Disease: a systematic review and meta-analysis
Source: BMC Geriatr. 2021 Sep 22;21:503. doi: 10.1186/s12877-021-02446-w (PMC8456607; doi:10.1186/s12877-021-02446-w)
Supplement: Supplementary file 4 — Additional file 4. Characteristics of studies in greater details. [file 12877_2021_2446_MOESM4_ESM.pdf]

Evidence of disease severity, cognitive and physical outcomes of dance interventions for persons with Parkinson's Disease: a systematic review and meta-analysis

Sophia Rasheeqa Ismail[1*], Shaun Wen Huey Lee[2], Dafna Merom[3], Puteri Sofia Nadira Megat Kamaruddin[1], Min San Chong[4], Terence Ong[4], Nai Ming Lai[2,5]

[1] Institute for Medical Research, National Institutes of Health, Ministry of Health, Malaysia

[2] School of Pharmacy, Monash University Malaysia, Malaysia

[3] University of Western Sydney, Australia

[4] University of Malaya Medical Centre, Malaysia.

[5] School of Medicine, Faculty of Health and Medical Sciences, Taylor's University Malaysia

Additional File 4

Characteristics of studies

Characteristics and risk of bias profile of included studies

Duncan 2012

**Methods**	Randomized controlled trial (US)
**Participants**	Diagnosis: Clinically defined "definite PD" (Hoehn& Yahr Stages I-IV) (n=62)<br>Location: Washington University Movement Disorders Center<br>Duration: 12 months<br>Exclusion criteria: 1) serious medical condition, 2) evidence of abnormality other than PD related changes on brain imaging (previously done for clinical evaluations), 3) history or evidence of neurological deficit other than PD, or 4) history or evidence of musculoskeletal problem.
**Interventions**	Intervention group: One hour twice weekly community-based Argentine Tango classes for 12 months. Participants danced both leader and follower roles, changed partners frequently, and learned new steps and/or integrated previously learned steps in new ways at each class throughout the 12 months.<br>Control group: No prescribed exercise and were instructed to go about their lives as usual.
**Outcomes**	**1. Disease Severity:** MDS-UPDRS sections 1-3 were used to measure disease severity. Section 1 examines non-motor experiences, section 2 covers ADLs, and section 3 assesses motor symptoms including tremor, rigidity, bradykinesia, gait, and postural instability. Higher scores indicate greater disease severity.<br><br>**2. Balance:** MiniBESTest (14-item tool measuring performance of dynamic balance tasks).<br>Lower scores indicate greater deficits in balance.<br><br>3. **Gait:** Freezing of Gait Questionnaire (FOGQ) for freezing gait, Six Minute Walk Test (6MWT) for walking endurance, and 4.87m GAITRite (CIR Systems, Inc, Havertown, PA) for walking velocity during comfortable forward, fast as possible forward, dual task, and backward walking.<br><br>4. **Upper Extremity Function:** Nine Hole Peg Test (9HPT)
**Notes**	

Risk of bias table

**Bias**	**Authors' judgement**	**Support for judgement**
Random sequence generation (selection bias)	Low risk	Randomisation was performed using an online random number generator
Allocation concealment (selection bias)	Unclear risk	No information was available to enable a meaningful assessment of the relationship between random sequence generation and allocation.
Blinding of participants and personnel (performance bias)	High risk	Not reported by the authors, although blinding was highly unlikely.
Blinding of outcome assessment (detection bias)	Unclear risk	Not reported
Incomplete outcome data (attrition bias)	High risk	There was high attrition rate in the Tango group (50%)
Selective reporting (reporting bias)	High risk	Other than MDS-UPDRS-3 and MiniBest test scores, all other outcome data were insufficiently reported to enable their inclusion in meta-analysis, as they were only reported in the form of F value and p value, without accompanying mean differences to enable a conversion to standard deviation, which is required for meta-analysis.
Other bias	Unclear risk	

Duncan 2014

**Methods**	Randomized controlled trial (US)

**Participants**	Diagnosis: "Definite" idiopathic Parkinson's Disease on levodopa<br>Age: Older than 40 years old<br>N: 10 participants<br>Sex: 4 out of 5 male in intervention and 4 out of 5 male in control group<br>Duration: 24 months<br>Control participants were matched by age, sex, and MDS-UPDRS III score to AT participants<br>Exclusion criteria: (1) a serious medical condition, (2) history or evidence of neurologic deficit other than PD, (3) evidence of brain abnormality other than PD-related changes on brain imaging (previously done, not a part of this study), or (4) history or evidence of a musculoskeletal problem that limited movement.
**Interventions**	Intervention group: Argentine Tango (AT)- twice-weekly, 1-hour community based sessions.<br><br>Control group: No prescribed exercise. Required to maintain their current levels of physical activity during the study.
**Outcomes**	Assessment of outcomes at baseline, 12 months and 24 months<br>**1. Disease severity**<br>- 1) MDS-UPDRS I : non-motor symptoms<br>- 2) MDS-UPDRS II : performance of ADLs<br>- 3) MDS-UPDRS III : motor symptoms<br><br>2. **Balance:** Mini-Balance Evaluation Systems Test (Mini-BESTest)<br><br>3. **Functional mobility**<br>- 1) GAITRite (CIR Systems, Sparta, NJ): walkway, walking velocity<br>- 2) Timed Up and Go (TUG)<br>- 3) dual-task TUG<br>- 4) Six-Minute Walk Test (6MWT)<br>- 5) Freezing of Gait Questionnaire (FOGQ).27
**Notes**	

Risk of bias table

**Bias**	**Authors' judgement**	**Support for judgement**
Random sequence generation (selection bias)	Unclear risk	Methods of random sequence generation was not reported
Allocation concealment (selection bias)	Unclear risk	No information was available to enable a meaningful assessment of the relationship between random sequence generation and allocation.
Blinding of participants and personnel (performance bias)	High risk	Blinding of participants and personnel was not reported, although it was highly unlikely.
Blinding of outcome assessment (detection bias)	Low risk	A trained physical therapist, blinded to group assignment, completed all assessments
Incomplete outcome data (attrition bias)	Low risk	All participants completed the assessments at baselines, 12 months and 24 months
Selective reporting (reporting bias)	Low risk	All outcome data were presented in graph form, and they had to be converted into an estimate of the numerical outcome data to enable their inclusion in meta-analysis.
Other bias	Unclear risk	

Foster 2013

**Methods**	Single-blind randomized controlled trial (US)
**Participants**	Diagnosis: All participants were diagnosed with idiopathic PD using published clinical diagnostic criteria.<br>N: 62 (intervention = 32, control = 30)<br>Sex: 15 out of 26 male in control group, 15 out of 26 male in intervention group<br>Inclusion criteria:<br>1. PD classified as Hoehn and Yahr stages I–IV 31, and experienced clear motor benefit from levodopa.<br>2. Participants had to be able to walk independently for 10 feet with or without an assistive device<br>Exclusion criteria:<br>1. History of neurological deficit other than PD<br>2. Serious medical problem(s)

	3. Evidence of abnormality other than PD-related changes on brain imaging<br>4. History or evidence of musculoskeletal or psychological problems
**Interventions**	Duration of intervention: 12 months<br>Intervention: Progressive Argentine tango lessons - one-hour dance classes two times per week for 12 months. Individuals with PD were paired within individuals who did not have PD. These dance partners were caregivers (e.g., spouses, family members) who accompanied PD participants to the classes and healthy young volunteers recruited from health-related graduate and undergraduate departments at Washington University in St. Louis (volunteers received special training on fall prevention and safety). All individuals, regardless of gender, were asked to dance in both the leader and follower roles to ensure that everyone spent similar amounts of time moving forward and backward. In addition, participants changed partners every ten minutes, a practice commonly used in dance classes to facilitate learning.<br>Control: Asked to continue the normal life routine that they had engaged in before enrolling in the study.
**Outcomes**	Assessment of outcomes at 3, 6 and 12 months.<br>Primary Outcome:<br>**1. Participation -** Activity Card Sort (ACS) **-** perceived level of participation in daily life activities as well as changes in participation in relation to certain events (e.g., the onset of disease or disability, beginning a new treatment regimen) or over specified periods of time (e.g., in the past five years).<br>Secondary Outcomes:<br>1. **Motor function** - Unified Parkinson's Disease Rating Scale (Sections 1-3)<br>**2. Depressive symptoms -** Beck Depression Inventory II
**Notes**	

Risk of bias table

**Bias**	**Authors' judgement**	**Support for judgement**
Random sequence generation (selection bias)	Unclear risk	Methods of random sequence generation was not reported
Allocation concealment (selection bias)	Unclear risk	No information was available to enable a meaningful assessment of the relationship between random sequence generation and allocation.
Blinding of participants and personnel (performance bias)	High risk	Blinding of participants and personnel was not reported, although it was highly unlikely.
Blinding of outcome assessment (detection bias)	Low risk	All assessments were conducted by a blinded rater
Incomplete outcome data (attrition bias)	Low risk	52/62 (83.9%) participants completed the study and were included in the analysis. The loss of follow-up was balanced between the two groups.
Selective reporting (reporting bias)	Low risk	All major and expected outcomes were presented in graph form without concurrent figures, and needed to be estimated from the graphs.
Other bias	Unclear risk	

Hackney 2007

**Methods**	Randomized controlled trial (US)
**Participants**	Diagnosis: idiopathic "definite" PD<br>N: 19<br>Sex: 6 out of 9 male in tango group, 6 out of 10 male in exercise group<br>Inclusion criteria:<br>1. Each subject also demonstrated clear benefit from PD medications.
**Interventions**	Duration of intervention : 13 weeks (or 20 sessions)<br>Intervention group: Progressive tango dance lessons<br>- two one-hour sessions per week for a total of 20 sessions completed within 13 weeks<br>Control group: Exercise lessons<br>- Structured strength/flexibility exercise classes designed for people with PD and/or elderly individuals (adapted fromFit 'N Fun)." (Subjects and training sessions, paragraph 1, lines 15-19)<br>- two one-hour sessions per week for a total of 20 sessions completed within 13 weeks.
**Outcomes**	Assessment of outcomes at baseline and after 20th training session<br>1. **Motor function :** UPDRS, Motor Subscale 3.<br>2. **Balance :** Berg Balance Scale<br>3. **Gait velocity :** 5-m path with and without a concurrent dual task

	4. **Mobility** : Timed Up and Go (TUG)<br>5. **Freezing of gait :** Freezing of Gait questionnaire.
**Notes**	

Risk of bias table

**Bias**	**Authors' judgement**	**Support for judgement**
Random sequence generation (selection bias)	Unclear risk	Methods of random sequence generation was not reported
Allocation concealment (selection bias)	Unclear risk	No information was available to enable a meaningful assessment of the relationship between random sequence generation and allocation.
Blinding of participants and personnel (performance bias)	High risk	Blinding of participants and personnel was not reported, although it was highly unlikely.
Blinding of outcome assessment (detection bias)	Low risk	All assessments were videotaped and all data files coded to allow for blinded ratings by a rater.
Incomplete outcome data (attrition bias)	Unclear risk	The number of participants analysed was not reported.
Selective reporting (reporting bias)	Low risk	All major and expected outcomes appeared to have been reported in sufficient detail.
Other bias	Unclear risk	

Hackney 2009

**Methods**	Randomized controlled trial (US)
**Participants**	Diagnosis: idiopathic "definite" PD<br>N: 58<br>Sex: 11 out of 17 male in waltz/foxtrot group, 11 out of 14 male in tango group, and 12 out of 17 male in control group<br>Inclusion criteria:<br>1. At least 40 years of age<br>2. Ability to stand for at least 30 min<br>3. Ability to walk independently for ≥ 3 m with or without an assistive device<br>4. PD with Hoehn and Yahr (H&Y) stages I–III<br>5. Demonstrated clear benefit from levodopa and were tested on medications at a standardized time to reduce the effects of medication-related fluctuations in performance.<br>Exclusion criteria:<br>1. History of neurological deficit other than PD.
**Interventions**	Duration of intervention: 20 completed sessions in 13 weeks<br>Intervention group 1: Waltz/foxtrot dance lessons<br>Intervention group 2: Tango dance lessons<br>Control group: No dance lessons
**Outcomes**	Assessment of outcomes during the week prior to the initiation of training lessons and during the week of completion of the 20th training lesson.<br>1. M**otor function :** UPDRS, Motor Subscale 3.<br>2. **Balance :** Berg Balance Scale<br>3. **Gait velocity :** 5-m path with and without a concurrent dual task<br>4. **Mobility** : Timed Up and Go (TUG)<br>5. **Freezing of gait :** Freezing of Gait questionnaire.<br>6. **Walking distance** : 6-minute walk test (6MWT)<br>7. **Forward and backward gai**t : 5 m instrumented, computerized GAITRite walkway (CIR Systems, Inc., Havertown, PA, USA).
**Notes**	

Risk of bias table

**Bias**	**Authors' judgement**	**Support for judgement**
Random sequence generation (selection bias)	High risk	The first author assigned individuals to waltz/foxtrot, tango and control by randomly selecting one of the 3 conditions from a hat.

Allocation concealment (selection bias)	High risk	The first author assigned individuals to waltz/foxtrot, tango and control by randomly selecting one of the 3 conditions from a hat.
Blinding of participants and personnel (performance bias)	Unclear risk	Participants were told only that they were participating in the study to obtain further information about the effects of exercise in those with PD. They were were not informed of the study hypothesis. We were unclear where this would have affected the outcome measured.
Blinding of outcome assessment (detection bias)	Low risk	The evaluations were videotaped for a rater who was a specially trained physiotherapy student otherwise not involved in the study
Incomplete outcome data (attrition bias)	Low risk	Low rate of attrition and reasons for missing outcome data unlikely to be related to true outcome
Selective reporting (reporting bias)	Low risk	All major and expected outcomes appeared to have been reported in sufficient detail.
Other bias	Unclear risk	

Hackney 2009b

**Methods**	Randomised controlled trial
**Participants**	Diagnosis: Idiopathic PD (Hoehn and Yahr stages of I-III)<br>N: 75<br>Age:<br>1. Waltz/Foxtrot: mean 66.8 (SE2.4) years<br>2. Tango: mean 68.2 (SE 1.4) years<br>3. Tai Chi: mean 64.9 (SE 2.3) years<br>4. No intervention: mean 66.5 (SE 2.8) years<br>Sex:<br>1. Waltz/Foxtrot: Male 11 / Female 6<br>2. Tango: Male 11 / Female 3<br>3. Tai Chi: Male 11 / Female 2<br>4. No intervention: Male 12 / Female 5<br>Inclusion criteria:<br>1. At least 40 years of age<br>2. Could stand for at least 30 minutes<br>3. Walk independently 3 or more meters with or without an assistive device<br>Exclusion criteria:<br>1. history of neurological deficit other than PD<br>2. participants received alterations in their medication schedules or doses
**Interventions**	Duration of intervention: 13 weeks<br>Intervention timing: 20 twice weekly one-hour sessions<br>Intervention group 1: Waltz/Foxtrot (n=19)<br>Intervention group 2: Tango (n=19)<br>Intervention group 3: Tai Chi (n=17)Control group: No intervention (n=20)
**Outcomes**	1. Health Related Quality of Life (HRQoL)
**Notes**	

Risk of bias table

**Bias**	**Authors' judgement**	**Support for judgement**
Random sequence generation (selection bias)	Low risk	The first author completed group assignment by randomly selecting one of the four conditions from a hat
Allocation concealment (selection bias)	Low risk	The first author completed group assignment by randomly selecting one of the four conditions from a hat
Blinding of participants and personnel (performance bias)	Unclear risk	Not reported
Blinding of outcome assessment (detection bias)	Unclear risk	Not reported
Incomplete outcome data (attrition bias)	High risk	Attrition rate was high (18.7%) and reasons for missing outcome data are likely to be related to true outcome
Selective reporting (reporting bias)	High risk	The major and expected outcomes were insufficiently reported in detail - especially for each of the different types of intervention.

Other bias	Unclear risk	

Hackney 2010

**Methods**	Randomized controlled trial (US)
**Participants**	Diagnosis: idiopathic "definite" PD (Hoehn and Yahr [H&Y] stages I-III)<br>N: 39<br>Inclusion criteria:<br>1. No history of other neurological deficits<br>2. At least 40 years of age<br>3. Able to stand for at least 30 minutes<br>4. Able to walk independently for 3 or more meters with or without an assistive device<br>5. Demonstrated clear benefit from levodopa
**Interventions**	"Both partner and non-partner tango classes began with identical standing warm-ups to upbeat Latin music. After warm-up, both classes listened to and danced to identical commercial tango music selections, in the same order of presentation. In the partner class, both sexes spent equal time leading and following dance steps, performed in a "closed practice" position, an adaptation of the traditional ballroom frame in which participants hold hands facing one another with bent elbows, maintaining forearms parallel to the floor. The non-partner group learned the same Argentine "leading" and<br>"following" tango-based steps as the partner group but performed them without a partner. The instructor advised participants to take breaks as needed. In the partnered dance class, participants with PD always danced with individuals without PD. These individuals included caregivers and loved ones who elected to participate<br>in classes as well as young adult volunteers. These volunteers were recruited from physical therapy, pre–physical therapy, and pre–medical programs at Washington<br>University in St. Louis. Caregivers, loved ones, and volunteers participated in the non-partner class as well." (Intervention, paragraph 2 & 3, lines 14-44)
**Outcomes**	Gait and balance.<br>"Participants were assessed on the Berg Balance Scale (BBS), tandem stance, 1-leg stance, the Timed Up and Go test, and the 6-minute walk test. Comfortable and fast-as-possible gait were assessed along a 5-m instrumented, computerized GAITRite walkway (CIR Systems, Inc, Havertown, PA). Variables of interest were<br>gait velocity, cadence, stride length, swing percentage, and double support percentage." (Testing protocol, paragraph 1, lines 11-18)
**Notes**	

Risk of bias table

**Bias**	**Authors' judgement**	**Support for judgement**
Random sequence generation (selection bias)	High risk	First author assigned individuals to the partnered or non-partnered dance class by randomly selecting a condition from a hat
Allocation concealment (selection bias)	High risk	First author assigned individuals to the partnered or non-partnered dance class by randomly selecting a condition from a hat
Blinding of participants and personnel (performance bias)	High risk	Participants knew which intervention they received (one group with partner and another without).
Blinding of outcome assessment (detection bias)	High risk	It was not clearly stated whether the assessors of the outcomes were blinded to the status of the allocation, but some of the outcomes were assessed by the participants themselves who knew the intervention that they received.
Incomplete outcome data (attrition bias)	High risk	31/39 (79.4%) participants were analysed. The rate of loss of follow up was considered too high.
Selective reporting (reporting bias)	Low risk	All major and expected outcomes appeared to have been reported in sufficient detail.
Other bias	Unclear risk	

Hulbert 2017

**Methods**	Randomized controlled trial (UK)
**Participants**	Diagnosis: Parkinson's disease (Hoehn and Yahr (H & Y) scale 1–3)

	N: 27 participants (intervention group = 15, control group = 12)<br>Sex: 5 out of 12 male in control group, 7 out of 15 male in intervention group<br>Exclusion criteria:<br>1. Not able to follow commands or remember instructions<br>2. Had uncorrected visual or hearing impairments<br>3. Unable to tolerate 90 min of data collection<br>4. Had other concurrent neurological conditions affecting their physical performance
**Interventions**	Duration of intervention: 10 weeks<br>Intervention group: Dance group<br>- Twice-weekly partnered dance classes of 1 hour, for 10 weeks.<br>- These covered the basic steps for beginner classes of ballroom and Latin American dance<br>Control group: Usual care
**Outcomes**	Assessment of outcomes were performed within 14 days prior to and after the 10-week intervention period.<br>1. **Whole body coordination during turning** - 12 on-the-spot turns at a self-selected pace<br>2. **Movement of eyes during turning** - VNG Ulmer<br>3. **Centre of pressure before and during the tur**n - Kistler force plate<br>4. **Number of turning steps, turn time, type and quality** - Standing Start 180' Turn Test
**Notes**	

Risk of bias table

**Bias**	**Authors' judgement**	**Support for judgement**
Random sequence generation (selection bias)	Low risk	Participants were randomly allocated by remote access telephone randomisation as part of the larger study to 2 groups; dance intervention alongside usual care (Dancers) and usual care only (Controls).
Allocation concealment (selection bias)	Low risk	Participants were randomly allocated by remote access telephone randomisation as part of the larger study to 2 groups; dance intervention alongside usual care (Dancers) and usual care only (Controls).
Blinding of participants and personnel (performance bias)	High risk	Blinding of participants and personnel was not reported, although it was highly unlikely.
Blinding of outcome assessment (detection bias)	Low risk	All data were re-coded with random numbers, allowing blinding of the group allocation and assessment time (pre/post) during the extraction process and interaction with the data. They were then un-coded for group and time analysis across the turning variables.
Incomplete outcome data (attrition bias)	Low risk	Low attrition rate and those lost were excluded due to unrelated reasons to the outcome.<br>" 25/27 participants completed the study and 24 data sets (12 dancers/12 controls) were included. One withdrew from the dance intervention due to un-related medical circumstances and one was excluded after deterioration in cognitive ability prevented accurate following of commands during assessment."
Selective reporting (reporting bias)	High risk	The outcomes of clinical interest, standing start 180 degree turn text, were not reported in sufficient details to enable data extraction for meta-analysis, as there was only f statistics and p value reported, without accompanying means to enable suitable derivation. The outcomes that were reported in sufficient details, namely, rotation of various body segments, were of uncertain clinical significance.
Other bias	Unclear risk	

Kunkel 2017

**Methods**	Randomised controlled trial (UK)
**Participants**	Diagnosis: Persons with Parkinson's<br>Age: mean 71 years (49-85)<br>Sex: 25 out of 51 were male<br>N = 51 participants (n=15 for controls and n=36 for experimental group)
**Interventions**	Duration of intervention = 10 weeks<br>Intervention group= Three ballroom (Social Foxtrot, Waltz, Tango) and 3 Latin American (Rumba, Cha Cha, Rock ‘n’ Roll) dances were taught by professional teachers in a dance centre. The classes lasted one hour, twice a week, for 10 weeks and PwP danced with their spouse, a friend or a volunteer.<br>"Control group participants were encouraged to continue with usual care, which typically comprised medication, attending medical clinics and routine visits from Parkinson’s nurses."
**Outcomes**	Assessment of outcomes at 3 months and 6 months

	**1. Balance**<br>**2. Confidence**<br>**3. Spinal posture**<br>**4. Mobility**<br>**5. Health outcome**<br>**6. Feasibility**<br>- 1) recruitment<br>- 2) retention<br>- 3) outcome measures<br>- 4) dance selection
**Notes**	Feasibility study published in abstract.

Risk of bias table

**Bias**	**Authors' judgement**	**Support for judgement**
Random sequence generation (selection bias)	Low risk	Methods, paragraph 3:<br>" ...randomization was completed in blocks: one block with 11 participants (eight dance and three controls); and three blocks with 13 participants (nine dance and four controls)"<br>"A second researcher (SH) (a physiotherapist with dance experience) obtained group allocations by telephone from the trial medical statistician". Although not clearly stated, the sequence generation was considered likely to be random.
Allocation concealment (selection bias)	Low risk	Methods, paragraph 3:<br>"A second researcher (SH) (a physiotherapist with dance experience) obtained group allocations by telephone from the trial medical statistician". Although not clearly stated, there appeared to be allocation concealment, as the allocation was carried out by an independent statistician.
Blinding of participants and personnel (performance bias)	High risk	Blinding of participants were not reported, although it was highly unlikely.
Blinding of outcome assessment (detection bias)	Low risk	Assessment was performed by an assessor who was unaware of participant group allocation.
Incomplete outcome data (attrition bias)	High risk	Although the drop-out rate was reasonable low with 6 out of 51 participants (11.8%) not analysed in the results, all 6 participants who were not analysed were from the dance group (6 out of 36 (16.7%)), while all 15 participants from the control group were included in the analysis. We considered the study as having high risk for incomplete outcome date due to the large differential rates of drop-out between groups.
Selective reporting (reporting bias)	Low risk	All major and expected outcomes appeared to have been reported in sufficient detail.
Other bias	Unclear risk	

Lee 2018

**Methods**	Randomized, blinded, waiting-list controlled partial crossover trial
**Participants**	Diagnosis: PD<br>N: 32<br>Age: mean 65.7 (SD 6.8) years<br>Sex: 58.5% female<br>Inclusion criteria:<br>1. age between 50 and 80 years<br>2. stage 1 to 3 on the Hoehn and Yahr scales3. no other neurological or cognitive impairments (K-MMES > 20)<br>4. not having received any exercise therapy within the 3 months prior to the study<br>Exclusion criteria: Not reported
**Interventions**	Duration of intervention: 8 weeks<br>Timing of intervention: 60-minute sessions twice a week<br>Intervention group 1: Turo PD group<br>Control group 1: Wait-list control
**Outcomes**	1. Motor function: UPDRS<br>2. Perceived health status: PDQL<br>3. Depression: Beck Depression Inventory (BDI)<br>4. Balance: Berg Balance Scale (BBS)

Notes	

Risk of bias table

Bias	Authors' judgement	Support for judgement
Random sequence generation (selection bias)	Low risk	The patients were assigned to either the Turo PD group or the waiting-list control group using computer-generated block randomization.
Allocation concealment (selection bias)	Unclear risk	Not reported
Blinding of participants and personnel (performance bias)	High risk	Blinding of participants were not reported, although it was highly unlikely.
Blinding of outcome assessment (detection bias)	Low risk	The assessors were blinded to the participants' treatment assignments.
Incomplete outcome data (attrition bias)	Low risk	Low rate of attrition and reasons for missing outcome data unlikely to be related to true outcome
Selective reporting (reporting bias)	High risk	All major and expected outcomes appeared to have been reported in sufficient detail
Other bias	Unclear risk	

McKee 2013

**Methods**	Sequentially randomised, controlled trial
**Participants**	Diagnosis: Idiopathic definite PD<br>Age: Control: mean 74.4 (6.5) years. Intervention: mean68.4 (7.5) years<br>Sex: Control: male 8 female 1. Intervention: male 12 female 12<br>Inclusion criteria:Participants (Hoehn and Yahr (H&Y) stages I–III) had no history of other neurological insult, were 50 years or older, and could walk 3+ meters with or without assistance.
**Interventions**	Duration of intervention: 12 weeks<br>Duration of each session: 90 minutes<br>Total number of sessions: 20 sessions<br><br>**Tango Group**<br>'Nine dance instructors without clinical qualifications participated in a 12-hour workshop on adapted tango methods, PD-specific motor impairments, and fall prevention, followed by an additional 3 hours of individual training from the senior author. Teachers were given an adapted tango manual, prepared for this study from former work about a 20h program as well as a 30h program. The manual delineates: PD motor impairments, fall risk and prevention, partnering enhancement and rhythmic entrainment, and a 20-class syllabus and format.'<br><br>**Education Group**<br>'Highly diverse health-related topics were delivered in a seminar designed to encourage extensive interaction and socialization. In 90-minute sessions, medical students and professors from local universities delivered one hour of lecture/discussion followed by one half hour of partnered and group learning through structured activities, question and answers, and further discussion. These seminars, on physical, mental and social wellbeing as well as contemporary scientific advances, included some PD-related information, and were moderated by a graduate student and several undergraduate students.
**Outcomes**	1. Cognitive measures: MoCA, Reverse Corsi Blocks, and Brooks Spatial Task<br>2. Disease severity, mobility and balance: UPDRS-III, Fullerton Advanced Balance Scale, Four-Square Step Test, Single/Dual timed up and go with single task (TUG), dual-cognitive (Cognitive-TUG: counting backward by 3s from a randomly generated number between 20 and 100) and dual-manual (Manual-TUG: carrying full cup of water) conditions and everyday fall incidence outside of class.
**Notes**	

Risk of bias table

Bias	Authors' judgement	Support for judgement
Random sequence generation (selection bias)	Unclear risk	While the authors stated that participants were assigned to the intervention and the control groups, there were no information on the manner and methods of assignment.

Allocation concealment (selection bias)	Unclear risk	While the authors stated that participants were assigned to the intervention and the control groups, there were no information on the manner and methods of assignment.
Blinding of participants and personnel (performance bias)	High risk	Blinding of personnel and participants were not reported, although this was highly unlikely.
Blinding of outcome assessment (detection bias)	Low risk	Methods, outcome measures: "All assessments were videotaped for blinded ratings by third year doctorate of physical therapy students and a medical student."
Incomplete outcome data (attrition bias)	Low risk	3 out of 33 participants (9.1%), all in the intervention group did not complete the required number of lessons. The authors included their data by way of imputation based on the last reading carried forward.
Selective reporting (reporting bias)	Low risk	3 out of 33 participants (9.1%), all in the intervention group did not complete the required number of lessons. The authors included their data by way of imputation based on the last reading carried forward.
Other bias	Unclear risk	

McNeely 2015

**Methods**	Controlled trial- it was unclear whether the allocation was randomised (US)
**Participants**	Diagnosis: idiopathic "definite" PD.<br>N: 19 (11 in intervention group and 8 in tango group)<br>Sex: 4 out of 8 male in both groups<br>Inclusion criteria:<br>1. Clear benefit from levodopa<br>2. Able to stand independently for at least 30 min<br>3. No evidence of dementia (MMSE ≥ 26)<br>4. No serious medical problem (aside from PD)<br>5. No evidence of abnormality on brain imaging (previously done for clinical evaluations – not part of this research)<br>6. No history or evidence of other neurological deficit, such as previous stroke or muscle disease<br>7. No deep brain stimulation<br>8. No other recent surgeries or injuries affecting movement.
**Interventions**	Duration of intervention: 12 weeks<br>**Intervention group: Dance for Parkinson's Disease (D4PD) Classes**<br>-Participants participated in 12 weeks of dance, meeting two times per week for 1 h each sessions<br>- Classes began with a 30-min seated warm-up in chairs. Seated movements focused on arm movements, foot and ankle movements, articulation of the spine and head, and facial expressions. Movements ranged in quality, sharp to continuous, and in speed. A cognitive activity challenging memory, rhythm, or sequence order also took place. This was followed by 5 min at the "barre," which for our class were the handrails of treadmills. The barre combinations concentrated on bigger movements, making shapes, extending through lines made by the body, bending of the knees, and testing/finding balance. The last 25 min were devoted to moving across the floor, integrating the whole body in coordinated movements. This portion of the class was often a mixture of choreographed sequences, improvisation, theatrical interpretation, and group dancing. Though the classes were based in ballet and modern dance, aspects of choreographic repertory, theater dance, jazz, tap, square dancing, Irish dancing, salsa, and flamenco were also incorporated. Teaching methods included verbal instruction, imagery, visualization, repetition, cognitive activities, and variations on movement, including direction, speed, quality of movement, and sequence. Each class included elements of improvisation and creativity, such as creating their own movement sequences, shaping movements of others as if they were "clay statues," mirroring each other's movement, passing a gesture around the circle, making an artistic choice, and building a group dance to music.<br>**Control group: Tango dance lessons**<br>"Tango classes began with a brief warm-up that focused on range of motion of all joints, trunk rotations, and weight shifts. This was followed by 45 min of instruction and partnered tango dancing. Participants danced both leading and following roles and changed partners to ensure that everyone spent time moving forward and backward and got experience dancing with different partners. In the leading role, participants practiced self-directed, internally generated movements, while the following role involved recognizing and responding to external cues from their dance partner. Traditional tango steps were often modified to accommodate the abilities and balance limitations of the participants (Hackney, 2010). Emphasis was placed on practicing weight shifts, walking backward, proper posture, rhythmic training, and moving with a partner. There was a brief cool down period at the end of each class. The tango classes were progressive in nature with new steps and sequences being added and integrated with known steps over the course of the intervention.
**Outcomes**	Outcomes assessment were performed before and after the 12-week dance intervention<br>1. **Motor Sign Severity & Quality of life -** MDS-UPDRS-III, Mini mental status exam (MMSE) & PDQ-39<br>**2. Balance** - Mini-BESTest<br>**3. Mobility**

	1) Time up and go (TUG)<br>2) 6-min walk test<br>3) four square step test<br>4) five times sit-to-stand test<br>**4. Gait -**<br>1) forward preferred speed<br>2) forward as fast as possible<br>3) backward<br>4) dual task walking
**Notes**	

Risk of bias table

**Bias**	**Authors' judgement**	**Support for judgement**
Random sequence generation (selection bias)	Unclear risk	The basis of allocation to two intervention groups was unclear.
Allocation concealment (selection bias)	Unclear risk	There was insufficient information to enable a meaningful assessment of the relationship between random sequence generation and allocation.
Blinding of participants and personnel (performance bias)	High risk	Blinding of personnel and participants were not reported, although this was highly unlikely.
Blinding of outcome assessment (detection bias)	High risk	Blinding of outcome assessment was not reported, although it was highly unlikely.
Incomplete outcome data (attrition bias)	Low risk	Low attrition rate and reasons for exclusion were not related to the outcome.<br>'Reasons for dropouts included a leg injury unrelated to the class, scheduling conflicts, and increased family member care responsibilities. The eight participants who completed the intervention were matched to a subset of participants with PD who participated in tango classes as part of a separate ongoing study'
Selective reporting (reporting bias)	Low risk	All major and expected outcomes appeared to have been reported in sufficient detail.
Other bias	Unclear risk	

Michels 2018

**Methods**	Randomised controlled study
**Participants**	Diagnosis: Idiopathic PD<br>N: 13<br>Age: Mean 69.2 (SD 8.7) years<br>Sex: 6 male and 7 female participants<br>Inclusion criteria:<br>1. Any Hoehn & Yahr (H&Y) stage or disease severity<br>2. On a stable PD medication regimen for at least one month prior to the study and continue that regimen without any changes throughout the course of the study.<br>Exclusion criteria:<br>1. Participated in any therapeutic dance intervention within the three months before the start of the study<br>2. Initiated any new PD treatments<br>3. Involvement in other PD-focused interventions throughout the course of the study<br>4. Significant cognitive impairment determined by a Montreal Cognitive Assessment score (MoCA) less than 24<br>5. Under the age of 18
**Interventions**	Duration of intervention : 10 weeks<br>Timing of intervention: 60 minutes weekly<br>Intervention group 1: Dance/movement Therapy (DT)<br>Control group 1: Support group control
**Outcomes**	1. Motor outcomes: UPDRS MDS, Berg Balance Scale (BBS), and Timed Up and GO (TUG)<br>2. Non-motor outcomes: MOCA, PDQ39, Beck Depression Inventory (BDI), Fatigue Severity Scale (FSS), Visual Analog Fatigue Scale
**Notes**	

Risk of bias table

Bias	Authors' judgement	Support for judgement
Random sequence generation (selection bias)	Unclear risk	Method of randomisation was not reported
Allocation concealment (selection bias)	Unclear risk	Method of allocation of study group was not reported
Blinding of participants and personnel (performance bias)	High risk	Blinding of personnel and participants were not reported, although this was highly unlikely.
Blinding of outcome assessment (detection bias)	Unclear risk	Outcome assessors were not specified
Incomplete outcome data (attrition bias)	Low risk	There were no missing patients reported
Selective reporting (reporting bias)	Low risk	All major and expected outcomes appeared to have been reported in sufficient detail.
Other bias	Unclear risk	

Poier 2019

**Methods**	Randomised controlled trial (Germany)
**Participants**	Study participants were recruited between 10/2015 and 06/2016, and consisted of "Individuals diagnosed with PD (aged between 50 and 90 years and with signed informed consent)." (Page 2:Paragraph 1, 'Methods – Study Population')<br>The following participants were excluded:" Patients with significant cognitive impairments (no independent completing of questionnaires) and/or patients who are permanently bound to a wheelchair/walker were not included."<br>Total number of participants: 29 (Tango: 14, Tai-Chi: 15).
**Interventions**	The intervention group received Argentinian Tango. Each lesson started with a tango-specific warm-up. Basic tango-thera- peutic contents were, e.g., communication with the partner, clear technique for going in different directions, dissociation of the left/right side of the body, good body axis, and dynamics. In both groups for each patient, a partner without PD (mostly wife/husband; in a few cases father, cousin, mother, etc.) was included in the intervention: The intervention lasted for one hour per week for 10 weeks.<br>The comparison group underwent Tai-Chi practice with the same frequency and duration.
**Outcomes**	The main outcome was quality of life, measured using the 39-item Parkinson's Disease Questionnaire (PDQ-39), as well as the The 10-item Brief Multidimensional Life Satisfaction Scale (BMLSS). The outcomes were measured at the beginning (baseline), in the middle (during intervention), and finally after the 10-week intervention (post-intervention). For this review, only the final outcome measure were included in our meta-analysis.
**Notes**	The study was stopped before scheduled completion at interim analysis due to the lack of significant benefit in the dance group, "To avoid further burden of patients by attending the interventions (which showed no significant benefit)" (Results, paragraph 3).

Risk of bias table

Bias	Authors' judgement	Support for judgement
Random sequence generation (selection bias)	Low risk	Methods, patient recruitement and randomization:<br>"A computer-generated list (statistician) was used for a random block allocation (block length 4 without strata)."
Allocation concealment (selection bias)	Low risk	Methods, patient recruitement and randomization:<br>"A computer-generated list (statistician) was used for a random block allocation (block length 4 without strata)." Although the involvement of the statistician in patient recruitement was not stated, we considered it as unlikely that the statistician would be directly involved in recruitment and patient allocation (hence the independence of sequence generation and allocation would most likely to have been preserved), and have therefore rated the study as having low-risk in allocation concealment.
Blinding of participants and personnel (performance bias)	High risk	Although not clearly stated, it was unlikely that the participants and personnel were blinded. In view of the main outcome of PDQ-39 being a subjective outcome, we rated the study as having high risk of performmance bias.

Blinding of outcome assessment (detection bias)	Unclear risk	It was not stated whether the researcher who assessed the outcome was blinded to the allocation status.
Incomplete outcome data (attrition bias)	High risk	Eight out of 29 (27%) of the participants, 4 in each group, dropped out during the study, which was by absolute standard a large proportion.
Selective reporting (reporting bias)	Low risk	The main outcomes specified in the methods (PDQ-39 and BMLSS) were reported in sufficient details in the results.
Other bias	Low risk	None identified.

Rawson 2019

**Methods**	Controlled trial with alternate allocation (US)
**Participants**	Diagnosis: Idiopathic PD<br>N: 119 (tango=43, treadmill=41, stretching =35)<br>Sex: 25 out of 39 in tango group, 17 out of 31 in treadmill group, and 14 out of 26 in stretching group<br>Inclusion criteria:<br>1. 30 years and older<br>2. clear benefit from levodopa<br>3. Hoehn & Yahr stages I to IV<br>4. ability to walk independently with or without an assistive device for at least 10 ft<br>5. no history of vestibular disease or dementia<br>6. diagnosis of “clinically definite PD.”<br>Exclusion criteria:<br>1. Medical condition for which exercise is contradicted<br>2. evidence of abnormality other than PD-related changes on brain imaging<br>3. history or evidence of neurological deficit other than PD such as stroke or muscle disease, or orthopedic or muscular problem
**Interventions**	Duration of intervention: twice per week for 12 weeks<br>**Intervention 1: Tango**<br>Participants practiced Argentine tango using an adapted curriculum for persons with PD. Initial classes focused on basic steps; more complex steps and sequences were added over 12 weeks. Participants were asked to change partners and to change roles from leader to follower several times during the course of each session. Dance partners were spouses, caregivers, volunteers, and laboratory staff. The syllabus was standardized and the same instructors taught all tango classes to maintain consistency.<br>**Intervention 2: Treadmill**<br>To approximate the intensity of activity in the tango classes, treadmill participants walked at their preferred overground walking speed. Preferred speed was assessed weekly and treadmill settingswere adjusted individually to match overground walking speeds. Treadmills were arranged in groups of 4 (2 pairs facing each other) to allow for social interactions.<br>**Control: Stretching**<br>This active control group focused on gentle stretching and whole-body flexibility exercises designed for people with PD. Exercises targeting strength were not included. All exercises were performed seated or standing with support to limit balance challenge.
**Outcomes**	Outcomes assessments were performed at baseline, after the 12-week intervention (posttest), and follow-up (12 weeks after posttest).<br>**1. Dynamic balance** - Mini-Balance Evaluation Systems Test (Mini- BESTest)<br>**2. Motor function** - MDS-UPDRS-III scores with Hoehn & Yahr staging<br>**3. Quality of life** - Parkinson Disease Questionnaire-39 (PDQ-39)<br>**4. Gait**<br>1) 5-m GAITRite walkway (CIR Systems Inc, Franklin, New Jersey).<br>2) forward (FWD)<br>3) backward (BKD)<br>4) 6-minute walk test (SMWT)
**Notes**	

Risk of bias table

**Bias**	**Authors' judgement**	**Support for judgement**
Random sequence generation (selection bias)	High risk	Serial enrolment (alternate allocation), rather than random allocation, was required to accommodate limited availability of exercise instructors and the need to complete the trial in a timely fashion.
Allocation concealment (selection bias)	High risk	Participants were recruited and serially assigned in a fashion similar to an alternate method to the exercise intervention currently enrolling by a research assistant.

Blinding of participants and personnel (performance bias)	High risk	Blinding of participants and personnel was not reported, although it was highly unlikely.
Blinding of outcome assessment (detection bias)	Low risk	Blinded ratings were used in analyses for the MDS-UPDRS-III and the Mini-BESTest. Videos were assigned unpatterned 10-character alphanumeric codes to ensure the MDS-certified rater was blinded to group, medication status and time point
Incomplete outcome data (attrition bias)	Unclear risk	Low attrition rate (22 drop outs out of 119 (18.5%)).
Selective reporting (reporting bias)	Low risk	All major and expected outcomes were reported in sufficient detail.
Other bias	Unclear risk	

Rios 2015

**Methods**	Randomized controlled trial (Canada)
**Participants**	Diagnosis: Idiopathic PD with Hoehn and Yahr stage I—III.<br>N:33 (control = 15, tango = 18)<br>Sex: 7 out of 15 in control group, and 12 out of 18 in tango group<br>Inclusion criteria:<br>1. Spoke either English or French sufficiently to fill out questionnaires and understand the instructions for dance classes<br>Exclusion criteria:<br>1. Could not stand for at least 30 min or walk for ≥3 m without an assistive device<br>2. Dementia (defined according to MDS dementia criteria)<br>3. Severe hearing and vision problems<br>4. Change in dopaminergic therapy over the preceding three months<br>5. Serious medical conditions which precluded dancing or could be worsened by exercise<br>6. More than 3 falls in the 12 preceding months (to ensure safety of intervention)<br>7. Other medical conditions which could affect study participation (e.g. drug abuse/alcoholism).
**Interventions**	Duration of intervention: 12 weeks<br>Intervention: Tango<br>- 1-h''traditional Argentine tango'' classes twice a week for 12 weeks<br>- Dance partners were primarily spouses and friends, who were healthy, without any exclusion criteria described above. For patients without an available partner, we provided partners who had some experience in tango<br>-Throughout the tango session patients continued with their usual physical activities and exercise program, but were instructed not to introduce new exercise programs or dancing classes.<br>Control: Instructions to exercise<br>- The control group was a wait-list group of patients with PD.<br>- Control participants followed their usual schedule of pharmacological treatment. In addition, they were provided a pamphlet about exercise in PD (''Exercises for people with Parkinson's'' Parkinson Society of Canada) and instructed to practice the exercises at home daily. If they were already engaged in intensive regular exercise programs, they were allowed to continue their usual schedule of exercise and were not required to start this new exercise program.
**Outcomes**	Outcomes assessment were performed at the end of the intervention, 3 months after its commencement.<br>**Primary Outcome**<br>1. **Motor function** - MDS-UPRDS-3<br>**Secondary Outcomes**<br>1) . **Motor/Gait**<br>**1. Off fluctuations and dyskinesia** - MDS-UPDRS.<br>**2. Balance** - Mini-Balance Evaluation Systems Test (Mini-BESTest-) of Dynamic Balance, Balance Evaluation—Systems Test.<br>**3. Gait & balance** - Timed Up and Go and Dual-task Timed Up and Go<br>4. **Fall** - Falls questionnaire (Canadian Community Health Survey(CCHS) — Healthy Aging (May, 2010) adapted to focus on the past 3 months<br>5. **Freezing gait** - Freezing of Gait Questionnaire (FOG Q).<br>6. **Upper extremity function** - Purdue Pegboard<br>**2) Cognitive/Mood outcomes**<br>1. **Cognitive dysfunction** - Montreal Cognitive Assessment (MoCA), using alternate versions (7.1—7.3, randomly-distributed order to limit training effects).<br>2. **Depression** - Beck Depression Inventory (BDI).<br>3. **Apathy** - Apathy Scale (AS).<br>**3.Fatigue -** Krupp Fatigue Severity Scale.<br>4. **Quality of life** - Parkinson's Disease Questionnaire-39 (PDQ-39).

	5. **Clincial impression of change** - Clinical Global Impression of Change (CGI-C), completed by both the examiner and the patient, with overall sever-ity of disease as the target symptom, scored from-3(severe worsening) to +3 (dramatic improvement).<br>6. Level of enjoyment - An exit questionnaire ranking level of enjoyment and over-all satisfaction with their dance/exercise program, scored from 1 (strongly agree) to 5 (strongly disagree), with open questions about willingness continuing practicing tango.
**Notes**	

Risk of bias table

**Bias**	**Authors' judgement**	**Support for judgement**
Random sequence generation (selection bias)	Low risk	Patients were randomised to either dance or exercise therapy via a random number generator.
Allocation concealment (selection bias)	Unclear risk	No relevant information was available to enable a meaningful assessment of the relationship between sequence generation and allocation.
Blinding of participants and personnel (performance bias)	High risk	This was not reported, although blinding was highly unlikely.
Blinding of outcome assessment (detection bias)	Unclear risk	Not reported.
Incomplete outcome data (attrition bias)	Low risk	All 33 participants that were randomised were included in the analysis.
Selective reporting (reporting bias)	Low risk	All major and expected outcomes were reported in sufficient detail.
Other bias	Unclear risk	

Rocha 2018

**Methods**	Randomised controlled trial
**Participants**	Diagnosis: Idiopathic PD<br>N: 21<br>Age:<br>1. Argentine tango group: mean 70.2 (SD5.5) years<br>2. Mixed dance group: mean 72.9 (SD5.5) years<br>Sex:<br>1. Argentine tango group: male 4 female 6<br>2. Mixed dance group: male 4 female 7<br>Inclusion criteria:<br>1. Rated I–IV on the modified Hoehn and Yahr scale<br>2. able to stand for at least 2 minutes<br>3. able to walk independently for more than 3 metres with or without assistive devices<br>Exclusion criteria:<br>1. Could not understand spoken English<br>2. Had scores on the Mini-Mental State Examination (MMSE) lower than 24 out of 30<br>3. Had any comorbidities that prevented exercise<br>4. Were unable to travel to the dance venue<br>5. Had previously received deep-brain stimulation surgery
**Interventions**	Duration of intervention: 8 weeks<br>Timing of intervention: 60-minute, in-person community dancing class once a week<br>Intervention group 1: Artegntine tango group<br>Control group 1: Mixed dance group<br>Co-intervention for both groups: Once-weekly, 40-minute, self-managed home dance programme.
**Outcomes**	1. Time Up and Go Test<br>2. Berg Balance Scale<br>3. Functional Gait Assessment<br>4. Freezing of Gait questionnaire<br>5. Movement Disorders Society Unified Parkinson's Disease Rating Scale sections II and III<br>6. 39-item Parkinson's Disease Questionnaire.
**Notes**	

Risk of bias table

**Bias**	**Authors' judgement**	**Support for judgement**

Random sequence generation (selection bias)	Low risk	A third party randomised the participants using computer-generated number sequences.
Allocation concealment (selection bias)	Unclear risk	Allocation of study group was not reported
Blinding of participants and personnel (performance bias)	Low risk	The dance teachers, assistants, and participants were blind to the aims of the trial and were not involved in any other aspects of the research.
Blinding of outcome assessment (detection bias)	Low risk	Participants were assessed at baseline by a blinded assessor 1 week before the start of the dancing programme. They were then assessed 1 week after the end of the programme by the same assessor.
Incomplete outcome data (attrition bias)	High risk	In view of the small sample size, the attrition rate was high (15%) and the reasons for missing outcome data are likely to be related to true outcome
Selective reporting (reporting bias)	Low risk	All major and expected outcomes were reported in sufficient detail.
Other bias	Unclear risk	

Shanahan 2017

**Methods**	Randomized controlled trial (Ireland)
**Participants**	Individuals with idiopathic Parkinson disease<br>- all criteria was mentioned in another publication<br>N: 90 (dance =45, and control = 45)<br>Sex: 13 out of 20 male in dance group, and 13 out of 21 male in control group
**Interventions**	Duration of intervention: 10 weeks<br><br>**Intervention group: Dance**<br>- Weekly 1.5-hour class for 10 weeks<br>- Classes were led by set dancing teachers who were also clinicians or experienced teaching clinical populations.<br>- Classes started with a warm-up, targeting movement speed and size, postural alignment, and other physiological systems required for dance.<br>- Exercises were progressed from sitting to standing according to abilities.<br><br>**Control group: Usual care**<br>The usual care group continued with their usual medication treatment. No additional intervention was offered to the usual care group.
**Outcomes**	Outcomes assessments were performed at around 3 months<br>**Primary outcome:**<br>1) **Feasibility**<br>**1.** Quantifying the success of randomization and allocation procedures.<br>2. Resources. -Availability and cost of buildings, dance studios, and personnel (researchers, assessors, independent mediator, dance partners, dance teachers, and health practitioners) were documented.<br>3. Success of recruitment - If 100 participants could be recruited in 1 year. T<br>4. Willingness of participants to be randomized<br>5. Attrition rates for the entire study<br>6. Safety<br>7. Adherence to the Irish set dancing intervention<br><br>**Secondary outcomes**<br>**1. Motor function -** Unified Parkinson's Disease Rating Scale<br>**2. Functional endurance -** 6-minute walk test<br>**3. Balance -** mini-BESTest<br>**4. Quality of life-** Parkinson's Disease Questionnaire-39 (PDQ-39)
**Notes**	

Risk of bias table

**Bias**	**Authors' judgement**	**Support for judgement**
Random sequence generation (selection bias)	Low risk	An independent mediator who was otherwise uninvolved and blinded to the hypothesis of the study was responsible for storing the sealed envelopes in an undisclosed location, generating the random allocation sequence, informing participants of their group allocation, and maintaining an undisclosed record of participant allocation.

Allocation concealment (selection bias)	Low risk	Eligible participants were randomly allocated to the dance or usual care group using sealed brown envelopes. An individual blinded to the hypothesis of the study prepared the envelopes by consequently placing an allocation form for each group into the envelopes. An independent mediator who was otherwise uninvolved<br>and blinded to the hypothesis of the study was responsible for storing the sealed envelopes in an undisclosed location, generating the random allocation sequence, informing participants of their group allocation, and maintaining an undisclosed record of participant allocation.
Blinding of participants and personnel (performance bias)	High risk	Participants were informed of their group allocation after completing baseline assessments.
Blinding of outcome assessment (detection bias)	Low risk	Assessors were blinded to group allocation.
Incomplete outcome data (attrition bias)	High risk	High attrition rate - after randomisation, 49 out of 90 participants dropped out (54.4%)
Selective reporting (reporting bias)	Low risk	Given that this study mainly assessed feasibility, all expected outcomes were reported in sufficient detail.
Other bias	Unclear risk	

Solla 2019

**Methods**	Randomised controlled trial (Italy)
**Participants**	Consecutive subjects with a definite diagnosis of PD were recruited from patients attending the outpatient Movement Disorders Clinic of the University of Cagliari (Total number randomised: 20). Inclusion criteria for the study included a clinical diagnosis of PD according to Gelb's criteria, a score ≤ 3 on the Hoehn and Yahr (H&Y) scale, ability to walk without walking aids, stable medication regimen in the 4 weeks before the study and a score ≥ 24 on the Mini-Mental State Examination. Exclusion criteria for the study were as follows: H&Y stage >3, diagnosis of dementia according to Diagnostic and Statistical Manual of Mental Disorders 5 (DSM-5) criteria, atypical parkinsonism, pharmacologic treatment with drugs not approved for PD, the presence of any complementary disability or autonomic problems that precluded the training program, or any specific health condition for which exercise was contraindicated.
**Interventions**	Intervention: Sardinian folk dance adapted for Parkinson's disease. The training program consisted of 24, 90-min class sessions, performed twice per week for 12 weeks.<br>Comparison group received usual care with medical therapy.
**Outcomes**	Motor disability was assessed using the motor component of Unified Parkinson's Disease Rating Scale Part-III (UDPRS-III). Functional performance was evaluated using a set of standardised tests, including the 6-minute walking test (6MWT) to evaluate cardiovascular fitness and the Five Times Sit-To-Stand Test (FTSST) to estimate dynamic strength in the lower limbs. Neuromotor performance was assessed using the Timed Up-and-Go (TUG) test for functional mobility and using the Berg Balance Scale (BBS) to evaluate static balance. Participant's lower-body joint mobility was assessed by the Sit-and-Reach Test (SRT), and the Back Scratch Test (BST) was used to assess upper-body joint mobility.
**Notes**	

Risk of bias table

**Bias**	**Authors' judgement**	**Support for judgement**
Random sequence generation (selection bias)	Low risk	Methods, Study design and participants, paragraph 2:<br>"Twenty patients meeting eligibility criteria (13M, 7F; mean age 67.4 – 6.1 years) were randomly allocated into two groups using a random number program generator (Research Randomizer 4.0 software)."
Allocation concealment (selection bias)	Unclear risk	No relevant information was available to enable a meaningful assessment of the relationship between sequence generation and allocation.
Blinding of participants and personnel (performance bias)	High risk	The authors stated that the study was a single-blind trial, but only he evaluators were said to have been blinded (see below). Although not clearly stated, participants and personnel were likely not blinded. As some of the outcomes assessed (PFS-16 and BDI-II) were subjective, we rated the study as having high risk of bias in this domain.
Blinding of outcome assessment (detection bias)	Low risk	Methods, Experimental procedures, paragraph 1:<br>"Evaluators were blinded to group allocation and not involved in routine clinical follow-up."

Incomplete outcome data (attrition bias)	Low risk	Only one out of 20 participants (control group) was excluded after study commencement, due to "too severe symptoms". Otherwise it appeared that all participants recruited were analysed.<br>Results, paragraph 1:<br>"Data from one participant in the control group were discarded after initial review as severe dyskinesia and freezing significantly altered the registration of gait patterns<br>during analyses".
Selective reporting (reporting bias)	Low risk	All outcomes pre-specified in the methods and expected from this study were reported in sufficient details in the results.
Other bias	Low risk	None identified.

Volpe 2013

**Methods**	Randomized controlled trial feasibility study (Italy)
**Participants**	Diagnosis: Idiopathic Parkinson's disease as diagnosed by a medical practitioner and were rated level 0–2.5 on the modified Hoehn & Yahr scale.<br>N: 24 (12 in each group)<br>Sex: 7 out of 12 in intervention group, and 6 out of 12 in control group<br>Inclusion criteria:<br>1.mild to moderately severe PD for safety reasons<br>2. Scocered more than 24/30 on the MMSE<br>Exclusion criteria:<br>1. did not speak Italian<br>2. co-morbidities that prevented dancing, mobility or safe exercise<br>3. received deep brain stimulation surgery<br>4. unable to travel to the dancing or physiotherapy venues)
**Interventions**	Duration of intervention : 6 months - 1.5hours per week<br>**Intervention group: Irish set dancing group**<br>- 90 minute set dancing class weekly for six months in a dance studio located in Venice.<br>- The Irish set dancing class included a preliminary warm up consisting of range of movement, balance and postural exercises. The protocol incorporated 10 minutes of warm up range of movement, balance and postural exercises, 70 minutes of Irish dance lessons and a 10 minutes cool down. Each person with PD was also given a video with recordings of the steps danced by the teacher. They were requested to watch the video at home once during each week, for a period of 1 hour.<br>**Control group: physiotherapy exercise**<br>- weekly standard physiotherapy exercise sessions included individual sessions delivered by a physiotherapist or physiotherapy assistant designed to improve muscle<br>strength, mobility, balance, and postural control. The physiotherapy program was in according to the KNGF Guidelines for physical therapy in Parkinson's disease.<br>- They were requested to watch the video at home once during each week, for a period of 1 hour.
**Outcomes**	Outcomes assessments performed at baseline (3 weeks prior to therapy), within 3 weeks of<br>the final week of the six month therapy period, and 3 weeks after discharge.<br>Primary outcome:<br>**1) Feasibility**<br><br>Secondary outcomes:<br>1) **Motor function** - UPDRS<br>2) **Gait** - Timed Up and Go<br>3) **Balance** - Berg Balance Scale<br>4) **Freezing gait** - Freezing of Gait Questionnaire<br>5) **Quality of life** - PDQ-39
**Notes**	

Risk of bias table

**Bias**	**Authors' judgement**	**Support for judgement**
Random sequence generation (selection bias)	Low risk	"We used a blocked stratified randomization procedure, based on the modified Hoehn & Yahr (1967) score, to allocate participants to one of the two groups. We used computer generated number sequences for randomization and this procedure was conducted by a third party."
Allocation concealment (selection bias)	Low risk	"Opaque envelopes were used to conceal allocation", and sequence generation was conducted by a "third party".
Blinding of participants and personnel (performance bias)	High risk	"Physiotherapists and dancing teachers providing the intervention could not be blinded to group allocation".

Blinding of outcome assessment (detection bias)	Low risk	Trained assessors who were blinded to group allocation conducted all of the assessments
Incomplete outcome data (attrition bias)	Low risk	Low attrition rate - Only 10.6% of sessions were not delivered due to personal reasons or due to illnesses not related to Parkinson's disease.
Selective reporting (reporting bias)	Low risk	Given that the main aim was feasibility, all expected outcomes were reported in sufficient detail.
Other bias	Unclear risk	

Footnotes

Shortlisted but excluded studies: main reasons for exclusion

Ajimsha 2014

**Reason for exclusion**	There is no dance element in the intervention group. Excluded on the basis of intervention.

Ashburn 2015

**Reason for exclusion**	Although the authors labelled the trial as a "randomised feasibility trial", the participants were drawn from a group of participants who joined dancing programme and another group who did not join. Although equal number of participants (n=7) were drawn from each group, the allocation of the participants to dancing or non-dancing group did not appear to be done at random, as the total participants who joined the dancing group was 35 and total who did not join was 15. This is a conference abstract. Excluded on the basis on study design.

Hackney 2018

**Reason for exclusion**	This study compares different forms of guiding (internal and external) during tango lessons and wellness education control group (that still performs the tango dance lessons). Excluded on the basis of intervention, as the nature of dance intervention in both groups were identical.

Kalyani 2019

**Reason for exclusion**	A quasi-experimental controlled on the effects of dance (Dance for Parkinson's Disease (D4PD)) on cognition, psychological symptoms and quality of life in Parkinson's Disease. Excluded on the basis of study design.

Monticone 2015

**Reason for exclusion**	The intervention involves multidisciplinary rehabilitation. There is no direct involvement of dance techniques in the intervention. Excluded on the basis of intervention.

Seidler 2016

**Reason for exclusion**	The study assessed the effects of telehealth application in a population where Tango was taught in both the intervention and the control group. Telehealth application is the intervention of interest. Excluded on the basis of intervention.

Characteristics of studies awaiting classification

Earhart 2010

**Methods**	Randomised controlled trial
**Participants**	Diagnosis: Idiopathic PD<br>N: 62 participants
**Interventions**	Duration of intervention: 3 months<br>Intervention group: community-based tango program<br>- twice weekly, one hour class<br>Control group: No exercise
**Outcomes**	1. Motor & non-motor function - MDS-UPDRS
**Notes**	Outcomes assessor was blinded to participant's group allocation

Foroud 2016

**Methods**	Nonrandomised controlled trial
**Participants**	N: 30 participants (18 PD and 12 healthy controls)
**Interventions**	Intervention: 5 months<br>- Weekly Dancing Parkinson's YYC
**Outcomes**	Functional behaviour:<br>1. pouring a drink<br>2. carrying a drink<br>3. getting dressed<br>4. moving about in room
**Notes**	

Garretto 2011

**Methods**	Non controlled trial
**Participants**	Diagnosis: Parkinson's disease (H&Y I - III)<br>N: 9 participants<br>Sex: 2 out of 7 participants were males
**Interventions**	Duration of intervention: 16 weeks<br>- 90 minutes weekly tango sessions
**Outcomes**	1. Motor function - UDPRS III<br>2. Balance - Berg Balance Scale<br>3. Gait - 15-minutes walk test<br>4. Quality of life - PDQ-39
**Notes**	Two out of 9 participants dropped out

Grosset 2016

**Methods**	Controlled trial
**Participants**	Diagnosis: Parkinson'sDisease<br>N: 140(70 in the intervention group, 70 in the control group)<br>Control group (age and sex matched index cases) comprises of caregivers, spouses or friends.
**Interventions**	Duration of intervention: Not specified<br>Intervention: Weekly one-hour dance classes
**Outcomes**	1. Quality of life - PDQ39<br>2. Life satisfaction
**Notes**	

Heiberger 2011

**Methods**	Non-controlled intervention trial

**Participants**	Diagnosis: Mild-moderate PD<br>N: 11 participants
**Interventions**	Duration of intervention: 8 months<br>- Once a week dance classes, lasting 1h15min each
**Outcomes**	1. Motor function - UPDRS<br>2. Gait - TUG<br>3. Balance - Semitandem-Test (SeTa)<br>4. Quality of life - Oregon QLS
**Notes**	

Krishnamurthi 2013

**Methods**	Non-randomised non-controlled trial
**Participants**	Diagnosis: Mild-moderate PD<br>N: 19 participants<br>Sex: 11 females and 8 males
**Interventions**	Duration of intervention: 10 weeks<br>- two 10h sessions per week
**Outcomes**	1. Gait - overground walking<br>2. Balance - dynamic weight shifts tasks<br>3. Range of motion - different joints
**Notes**	15 out of 19 participants completed the study

Kunte 2018

**Methods**	Non-randomised controlled trial
**Participants**	Diagnosis: Parkinson’s disease (Hoehn &Yahr stage- II to IV)<br>N: 34 (17 in intervention, and 17 in control group)
**Interventions**	Duration of intervention: 12 weeks<br>Intervention group: Dance intervention study<br>- Once a week, 1-hour group therapy sessions<br>Control group: Traditional exercise group therapy
**Outcomes**	1. physical functioning<br>2. cognitive abilities<br>3. anxiety and depression<br>4. mood : PANAS and HADS<br>5. quality of life
**Notes**	1 out of 17 participant in the intervention group was lost to follow up<br>6 out of 17 participants in the control grouo was lost to follow up<br>Method of sampling: Convenience sampling

Rodriguez-Quiroga 2013

**Methods**	Noncontrolled trial
**Participants**	Diagnosis: Parkinson's disease<br>N: 20 participants
**Interventions**	Duration of intervention:16 weeks<br>- 120minutes tango sessions per week
**Outcomes**	1.Motor function: UPDRS and MOCA<br>2. Gait and balance - GABS, six minute-walk test<br>3. Quality of life - PDQ-39
**Notes**	2 out of 20 participants dropped out

Characteristics of ongoing studies

Capato 2015

**Study name**	Randomized controlled trial protocol: balance training with rhythmical cues to improve and maintain balance control in Parkinson's disease
**Methods**	Parallel, prospective, single-blind, randomized clinical trial.
**Participants**	"We will examine 150 PD patients before and after training.<br><br>**Inclusion criteria**<br>All of the subjects will be diagnosed by neurologists from the Movement Disorders Ambulatory Clinic of the University of São Paulo Faculty of Medicine Clinics Hospital, according to the UK Brain Bank criteria and they should be at H&Y classification stage II or III, with a Mini Mental Status Examination (MMSE) and they should have a score of above 24. All patients should also present fall history in the past months. They should have the capacity to ambulate independently indoors without aid.<br><br>**Exclusion criteria**<br>These are the presence of neurological, orthopedic or cardiopulmonary problems, an unstable medication regime, and an inability to understand or adhere to the protocol because of, for example, cognitive, auditory or visual problems. Patients receiving physical therapy training will also be excluded from the training." (Participants, lines 1-18)
**Interventions**	"Subjects were randomly distributed among three groups. The first experimental group will be led by a physiotherapist, it will receive motor skill training with rhythmical<br>auditory cues marked by a metronome (GBRT); the second experimental group will receive the same training without rhythmical cues (MT); and the control group (CT) will receive exercises in general orientation only with a general orientation." (Design and procedures, paragraph 1, lines 5-12)
**Outcomes**	"The primary outcome is balance, which will be assessed by the Berg Balance Scale (BBS), postural stress test (PST), push and release test (PRT) and Mini BESTest (MBESTest). The secondary outcome is gait, which will be evaluated by the timed up and go test (TUG) and by the freezing of gait, using the Freezing of Gait Questionnaire. Independence in activities of daily living (ADLs), and motor performance will be assessed by the unified Parkinson's disease rating scale<br>(UPDRS). Falls and fear of falling will be evaluated during a variety of everyday activities and measured by the Falls Efficacy Scale-International (FES-I). The number of falls will be registered by the physiotherapist at the hospital and by the patient at home. A weekly follow-up of falling will be conducted." (Outcome measures and test procedure, paragraphs 1 & 2, lines 1-15)
**Starting date**	
**Contact information**	taminec@yahoo.com.br<br>1. Department of Physical therapy, University of São Paulo, Av Dr Enéias de Aguiar, 255 – 05403.000 São Paulo, São Paulo, Brazil<br>2. PHYSICAL, Rua Cubatão 929 conj, 142 - 04013-043 São Paulo, São Paulo, Brazil
**Notes**	

Capato 2016

**Study name**	Randomized controlled trial protocol: Balance training with rhythmical cues to improve and maintain balance control in Parkinson's disease
**Methods**	Single-blind randomised study
**Participants**	Diagnosis: Parkinson's Disease H&Y Stages II-III<br>Inclusion criteria:<br>1. Asymptomatic for depression and dementia<br>2. History of fall<br>3. 50 years to 70 years<br>4. capacity to ambulate independently indoors without aid.<br>Exclusion criteria:<br>1. These are the presence of neurological, orthopedic or cardiopulmonary problems, an unstable medication regime, and an inability to understand or adhere to the protocol because of, for example, cognitive, auditory or visual problems.<br>2. Patients receiving physical therapy training will also be excluded from the training.
**Interventions**	Three study groups:<br>**Group 1: Balance training with rhythmical (BRT)**<br>- the BRT group received a motor program to improve balance associated with rhythmical auditory cues (RAC)<br>- The exercise program specific to balance is of 5 weeks' duration with two sessions per week, 45 minutes each, and consists of general physiotherapy exercises. Each session is divided into five warm-up minutes—30 minutes for the main part and 10 minutes for the cool down. The training progresses

	and intensifies each week depending on the individual's performance. The subjects should be able to execute 10 repetitions of the exercise sequences correctly to progress to the next movement.<br>**Group 2: Motor training (MT)**<br>- the MT group performed motor training with the same aims as those in the BRT group but without RACs<br>Group 3: Control group (CG)<br>- the control group (CG) was trained only in orientations
**Outcomes**	Assessment of outcome: before and after 10th training session, and 4 and 30 weeks after end of training<br>Primary outcome: Balance - Mini BESTest<br>Secondary outcomes:<br>1. Balance - Berg Balance Scale<br>2. Motor and non-motor function - UPDRS<br>3. Gait and agility - Time Up and Go test<br>4. Posture - Push and Release Test<br>5. Freezing - New Freezing of gait questionnaire<br>6 Fear of falling - Falls Efficacy Scale-International (FES-I)
**Starting date**	
**Contact information**	
**Notes**	Randomisation method: Computer-generated random-sequence table<br>Study completed in Nov 2018
