# Supplementary material for: Evidence of disease severity, cognitive and physical outcomes of dance interventions for persons with Parkinson’s Disease: a systematic review and meta-analysis
Source: BMC Geriatr. 2021 Sep 22;21:503. doi: 10.1186/s12877-021-02446-w (PMC8456607; doi:10.1186/s12877-021-02446-w)
Supplement: Supplementary file 5 — Additional file 5. Summary of findings tables with certainty of evidence rating. [file 12877_2021_2446_MOESM5_ESM.pdf]

Evidence of disease severity, cognitive and physical outcomes of dance interventions for persons with Parkinson's Disease: a systematic review and meta-analysis

Sophia Rasheeqa Ismail[1*], Shaun Wen Huey Lee[2], Dafna Merom[3], Puteri Sofia Nadira Megat Kamaruddin[1], Min San Chong[4], Terence Ong[4], Nai Ming Lai[2,5]

[1] Institute for Medical Research, National Institutes of Health, Ministry of Health, Malaysia

[2] School of Pharmacy, Monash University Malaysia, Malaysia

[3] University of Western Sydney, Australia

[4] University of Malaya Medical Centre, Malaysia.

[5] School of Medicine, Faculty of Health and Medical Sciences, Taylor's University Malaysia

Additional File 5

Summary of findings tables with certainty of evidence rating

Table 1 Dance compared to no dance/ usual care for people with Parkinson's Disease

**Dance compared to no dance/ usual care for people with Parkinson's Disease**							
**Patient or population:** people with Parkinson's Disease<br>**Setting:**<br>**Intervention:** Dance<br>**Comparison:** usual care							
**Outcomes**	**Anticipated absolute effects[*] (95% CI)**		**Relative effect (95% CI)**	**№ of participants (studies)**	**Certainty of the evidence (GRADE)**	**Comments**
	**Risk with usual care**	**Risk with Dance**				
Disease severity (measured using MDS-UPDRS 1 , non-motor experiences of daily living)	The mean disease severity (measured using MDS-UPDRS I Intellectual function, mood, behavior) ranged from 9.3 to 14.8 points	MD 3.5 points lower (18.68 lower to 11.67 higher)	-	23 (2 RCT)	⊕⊕⊖⊖ LOW [3 4]	
Disease severity (measured using MDS-UPDRS 2, motor experiences of daily living)	The mean disease severity (measured using MDS-UPDRS II activities of daily living) ranged from 11 to 17.2 points	MD 2.09 points lower (7.57 lower to 3.40 higher)	-	23 (2 RCT)	⊕⊕⊖⊖ LOW [3 4]	
Disease severity (measured using MDS-UPDRS 3 motor examination) - At 3 months	The mean disease severity (measured using MDS-SPDRS III motor subscale) - At 3 months ranged from 15.6 to 45.6 points	MD 6.91 points lower (9.97 lower to 3.84 lower)	-	148 (5 RCTs)	⊕⊕⊖⊖ LOW [3 4]	
Disease severity (measured using MDS-UPDRS 3 motor examination) - At 6 months	The mean disease severity (measured using MDS-SPDRS III motor subscale) - At 6 months ranged from 29.3-45.2 points	MD 7.26 points lower (11.68 lower to 2.85 lower)	-	131 (3 RCTs)	⊕⊖⊖⊖ LOW [3 4]	
Disease severity (measured using MDS-UPDRS 3 motor examination) - At 12 months	The mean disease severity (measured using MDS-SPDRS III motor subscale) - At 12 months ranged from 45-46 points	MD 14.91 points lower (19.78 lower to 10.05 lower)	-	62 (2 RCTs)	⊕⊕⊖⊖ LOW [3 4]	
Disease severity (MDS-UPDRS 4- dyskinesia)	The mean disease severity (MDS-UPDRS IV- dyskinesia) was 0.7 points	MD 0.1 points lower (0.79 lower to 0.59 higher)	-	33 (1 RCT)	⊕⊕⊖⊖ LOW [3 4]	
Disease severity (MDS-UPDRS 4- fluctuation)	The mean disease severity (MDS-UPDRS IV- fluctuation) was 1.1 points	MD 0.6 points higher (0.92 lower to 2.12 higher)	-	33 (1 RCT)	⊕⊕⊖⊖ LOW [1 2]	
Balance (MiniBest Test)	The mean balance (MiniBest Test) ranged from 16.7-31.3 points	MD 4.47 points higher (2.29 higher to 6.66 higher)	-	95 (3 RCTs)	⊕⊕⊕⊖ MODERATE [3]	
Balance (Berg Balance Scale)- at 3 months	The mean balance (Berg Balance Scale) ranged from 36.6 to 49 points	MD 8.42 points higher (3.68 higher to 13.17 higher)		32 (2 RCT)	⊕⊖⊖⊖ LOW	
Balance (Berg Balance Scale)- at 4 months	The mean balance (Berg Balance Scale) was 47 points	MD 5.10 points higher (no difference to 10.20 higher)	-	48 (1 RCT)	⊕⊖⊖⊖ LOW [5 6]	
Balance (Berg Balance Scale)- at 6 months	The mean balance (Berg Balance Scale) was 52 points	MD 1.60 points lower (4.54 lower to 1.34 higher)		46 (1 RCT)	⊕⊖⊖⊖ LOW [5 6]	

Activity specific balance confidence	The mean activity specific balance confidence was 75.4 points	MD 0.2 points higher (12.72 lower to 13.12 higher)	-	46 (1 RCT)	⊕⊕⊖⊖ LOW [1 2]	
Freezing of gait questionnaire	The mean freezing of gait questionnaire ranged from 5.7-8 points	MD 0.38 points lower (2.99 lower to 2.24 higher)	-	89 (3 RCTs)	⊕⊕⊖⊖ LOW [4 6]	
Timed Up and Go Test (TUG)	The mean timed Up and Go Test (TUG) ranged from 7-14.4 seconds	MD 1.16 seconds lower (2.17 lower to 0.15 lower)	-	200 (7 RCTs)	⊕⊕⊖⊖ LOW [3 7]	
Five Times Sit-to-Stand Test (FTSTT)	The mean time (seconds) for completion of Five Times Sit-to-Stand Test (FTSTT) was 11.5 seconds	MD 4.90 seconds lower (6.51 lower to 3.29 lower)		19 (1 RCT)	⊕⊕⊖⊖ LOW [2,3]	
Sit-and-Reach Test (SRT)	The mean gap (cm) in Sit-and-Reach Test (SRT) was 5.9 cm	MD 4.60 cm longer (2.78 shorter to 12.98 longer)		19 (1 RCT)	⊕⊕⊖⊖ LOW [2,3]	
Walking distance: six-minute-walk test (Tango with other dance types)	The mean walking distance: six-minute-walk test ranged from 360.9-470 meters	MD 1.34 meters lower (53.91 lower to 51.24 higher)	-	104 (3 RCTs)	⊕⊕⊖⊖ LOW [3 8]	
Walking distance: six-minute-walk test (Sardinian folk dance)	The mean walking distance: six-minute-walk test was 331.4 meters	MD 238.8 meters more (158.0 more to 319.6 more)		19 (1 RCT)	⊕⊕⊖⊖ LOW [2,3]	
Forward velocity (meter/second)	The mean forward velocity (meter/second) ranged from 0.91-1.1 meter/second	MD 0 meter/second (0.11 lower to 0.11 higher)	-	48 (1 RCT)	⊕⊕⊖⊖ LOW [2 9]	
Standing-start 180 degree turn test (number of steps)	The mean standing-start 180 degree turn test (number of steps) was 4 steps	MD 1.3 steps higher (0.38 lower to 2.98 higher)	-	46 (1 RCT)	⊕⊕⊖⊖ LOW [2 3]	
Standing-start 180 degree turn test (second)	The mean standing-start 180 degree turn test (second) was 1.9 seconds	MD 0.4 seconds higher (0.18 lower to 0.98 higher)	-	46 (1 RCT)	⊕⊕⊖⊖ LOW [2 3]	
Back Scratch Test (BST) (cm)	The mean gap (cm) in the Back Scratch Test (BST) was 13.9 cm.	MD 5.30 cm longer (2.94 shorter to 13.54 longer)		19 (1 RCT)	⊕⊕⊖⊖ LOW [2,3]	
Spinal mouse: inclination degree	The mean spinal mouse: inclination score ranged from 7.9 degree	MD 0.8 degree higher (3.61 lower to 5.21 higher)	-	46 (1 RCT)	⊕⊕⊖⊖ LOW [2 3]	
Adverse events: falls during study	Study population		RR 0.56 (0.11 to 2.90)	33 (1 RCT)	⊕⊕⊕⊖ MODERATE [2]	
	200 per 1,000	112 per 1,000 (22 to 580)				
Cognitive function: Montreal Cognitive Assessment Scale Scale from: 0 to 30 (higher better function)	The mean cognitive function: Montreal Cognitive Assessment Scale ranged from 26.1 to 26.3 points	MD 0.62 points higher (0.84 lower to 2.08 higher)	-	64 (2 RCT)	⊕⊕⊖⊖ LOW [1 10]	
Quality of life: PDQ 39	The mean quality of life: PDQ 39 ranged from 17.7 to 18.5 points	MD 2.25 points higher (3.69 lower to 8.19 higher)	-	77 (2 RCT)	⊕⊕⊖⊖ LOW [3 5]	
Depressive symptoms: Beck Depression Inventory	The mean depressive symptoms: Beck Depression Inventory ranged from 5.5 to 16 points	MD 0.51 points higher (4.90 lower to 5.91 higher)	-	86 (3 RCT)	⊕⊕⊖⊖ LOW [3 5]	
Apathy (Apathy Scale)	The mean apathy (Apathy Scale) was 29.4 points	MD 1.9 points higher	-	33 (1 RCT)	⊕⊕⊖⊖ LOW [1 2]	

		(1.74 lower to 5.54 higher)				
Fatigue: Krupp Fatigue Severity Scale	The mean fatigue: Krupp Fatigue Severity Scale was 35.9 points	MD 5.1 points lower (14.39 lower to 4.19 higher)	-	33 (1 RCT)	⊕⊕⊖⊖ LOW [1,2]	
Activity participation: total current activity participation (Activity Card Sort score)	The mean activity participation: total current activity participation (Activity Card Sort score) was 46 points	MD 8 points higher (4.4 lower to 20.4 higher)	-	52 (1 RCT)	⊕⊕⊖⊖ LOW [1,2]	
*__The risk in the intervention group__ (and its 95% confidence interval) is based on the assumed risk in the comparison group and the **relative effect** of the intervention (and its 95% CI).<br><br>**CI:** Confidence interval; **RR:** Risk ratio; **OR:** Odds ratio;						
**GRADE Working Group grades of evidence**<br>**High certainty:** We are very confident that the true effect lies close to that of the estimate of the effect<br>**Moderate certainty:** We are moderately confident in the effect estimate: The true effect is likely to be close to the estimate of the effect, but there is a possibility that it is substantially different<br>**Low certainty:** Our confidence in the effect estimate is limited: The true effect may be substantially different from the estimate of the effect<br>**Very low certainty:** We have very little confidence in the effect estimate: The true effect is likely to be substantially different from the estimate of effect						

Footnotes

[1] The single included study has high risk of bias in blinding of participants and personnel for this outcome which required subjective assessment, and unclear risk of selection bias.

[2] A single small included study with a wide 95% confidence interval.

[3] The included study/studies had high risk in blinding of participants and personnel for this outcome, which required an assessment from the personnel.

[4] Small number of included studies and cumulative sample, with wide 95% confidence interval.

[5] Wide 95% confidence interval, ranging from moderately lower to slightly higher score

[6] There was high risk of bias in blinding of participants and personnel in the included study.

[7] There was substantial heterogeneity, as indicated by a high I square statistics.

[8] The 95% confidence interval was wide, ranging from moderately lower to moderately higher distance

[9] The single included study had unclear risk of bias in blinding of participants and personnel and high risk of selection bias.

[10] The 95% confidence interval included a modest reduction to a moderate increase in score.

2 Two different forms of dance interventions

Table 2 Dance for Parkinson's disease (D4PD) compared to Tango for people with Parkinson's Disease

**Dance for Parkinson's disease (D4PD) compared to Tango for people with Parkinson's Disease**						
**Patient or population:** people with Parkinson's Disease<br>**Setting:**<br>**Intervention:** Dance for Parkinson's disease (D4PD)<br>**Comparison:** Tango						
**Outcomes**	**Anticipated absolute effects[*] (95% CI)**		**Relative effect (95% CI)**	**№ of participants (studies)**	**Certainty of the evidence (GRADE)**	**Comments**
	**Risk with Tango**	**Risk with Dance for Parkinson's disease (D4PD)**				
Disease severity (measured using MDS-UPDRS III- motor examination)	The mean disease severity (measured using MDS-UPDRS III- motor examination) was 23.3 points	MD 6.1 points higher (7.42 lower to 19.62 higher)	-	16 (1 RCT)	⊕⊕⊖⊖ LOW [1,2]	
Time Up and Go Test (TUG) (seconds)	The mean time Up and Go Test (TUG) (seconds) was 9.6 seconds	MD 0.3 seconds lower (1.63 lower to 1.03 higher)	-	16 (1 RCT)	⊕⊕⊖⊖ LOW [1,2]	
Six-minute walk test (meters)	The mean six-minute walk test (meters) was 453.5 meters	MD 36.3 meters higher	-	16 (1 RCT)	⊕⊕⊖⊖ LOW [1,2]	

		(49.15 lower to 121.75 higher)				
Gait velocity (meter/second)	The mean gait velocity (meter/second) was 1.4 meters/second	MD 0.15 meters/second higher (0.1 lower to 0.4 higher)	-	16 (1 RCT)	⊕⊕⊝⊝ LOW [1 2]	
Quality of life (PDQ 39)	The mean quality of life (PDQ 39) was 107.6 points	MD 23.5 points higher (64.83 lower to 111.83 higher)	-	16 (1 RCT)	⊕⊕⊝⊝ LOW [1 2]	
***The risk in the intervention group** (and its 95% confidence interval) is based on the assumed risk in the comparison group and the **relative effect** of the intervention (and its 95% CI).<br><br>**CI:** Confidence interval; **RR:** Risk ratio; **OR:** Odds ratio;						
**GRADE Working Group grades of evidence**<br>**High certainty:** We are very confident that the true effect lies close to that of the estimate of the effect<br>**Moderate certainty:** We are moderately confident in the effect estimate: The true effect is likely to be close to the estimate of the effect, but there is a possibility that it is substantially different<br>**Low certainty:** Our confidence in the effect estimate is limited: The true effect may be substantially different from the estimate of the effect<br>**Very low certainty:** We have very little confidence in the effect estimate: The true effect is likely to be substantially different from the estimate of effect						

Footnotes

[1] The included study had high risk of bias in blinding of participants and personnel, and unclear risk of selection bias.

[2] A single small included study with wide 95% confidence interval ranges from substantially lower to substantially higher estimate.

Table 3 Tango compared to mixed dances for people with Parkinson's Disease

**Tango compared with mixed dances for people with Parkinson's Disease**						
**Patient or population: People with Parkinson's Disease**<br>**Settings:**<br>**Intervention: Tango**<br>**Comparison: Mixed dances**						
**Outcomes**	**Anticipated absolute effects* (95% CI)**		**Relative effect (95% CI)**	**No of Participants (studies)**	**Quality of the evidence (GRADE)**	**Comments**
	**Risk with mixed dances**	**Risk with Tango**				
**Time Up and Go Test (TUG) seconds**	The mean TIme Up and Go test was 9.6 seconds	MD 1.60 seconds lower (3.75 lower to 0.55 higher)		18 (1 RCT)	⊕⊕⊝⊝ **low**[1,2]	
**Functional Gait Assessment (FGA)**	The meanfunctional gait assessment was 23.2 points	MD 1.60 points higher (3.39 lower to 6.59 higher)		18 (1 RCT)	⊕⊕⊝⊝ **low**[1,2]	
**Freezing gait questionnaire** Scale from 0 to 24 (lower better)	The mean freezing gait questionnaire was 5.3 points	MD 1.60 points higher (3.95 lower to 7.15 higher)		18 (1 RCT)	⊕⊕⊝⊝ **low**[1,2]	
**Berg Balance Scale (BBS)**	The mean Berg Balance Scale was 50 points	MD 2.30 points higher (2.21 lower to 6.81 higher)		18 (1 RCT)	⊕⊕⊝⊝ **low**[1,2]	
**Disease severity (measured using MDS-UPDRS 2, motor experiences of daily living)**	The mean disease severity measured using MDS-UPDRS2 was 10.8 points	MD 1.20 points higher (6.21 lower to 8.61 higher)		18 (1 RCT)	⊕⊕⊝⊝ **low**[1,2]	
**Quality of life (PDQ-39)**	The mean quality of life was 28.5 points	MD 8.10 points lower (18.08 lower to 1.88 higher)		18 (1 RCT)	⊕⊕⊝⊝ **low**[1,2]	
*The basis for the **assumed risk** (e.g. the median control group risk across studies) is provided in footnotes. The **corresponding risk** (and its 95% confidence interval) is based on the assumed risk in the comparison group and the **relative effect** of the intervention (and its 95% CI).<br>**CI:** Confidence interval; **RR:** Risk Ratio;						

GRADE Working Group grades of evidence<br>**High quality:** Further research is very unlikely to change our confidence in the estimate of effect.<br>**Moderate quality:** Further research is likely to have an important impact on our confidence in the estimate of effect and may change the estimate.<br>**Low quality:** Further research is very likely to have an important impact on our confidence in the estimate of effect and is likely to change the estimate.<br>**Very low quality:** We are very uncertain about the estimate.

Footnotes

1 A small single included study with wide 95% confidence interval, ranging from substantially lower to substantially higher estimates on either end. Downgraded one level

2 The single study had high attrition rate thereby contributing to the high risk of attrition bias. Downgraded one level

Table 4 Partnered compared to non-partnered dance for people with Parkinson's Disease

**Partnered compared to non-partnered dance for people with Parkinson's Disease**						
**Patient or population:** people with Parkinson's Disease<br>**Setting:**<br>**Intervention:** Partnered<br>**Comparison:** non-partnered dance						
**Outcomes**	**Anticipated absolute effects* (95% CI)**		**Relative effect (95% CI)**	**№ of participants (studies)**	**Certainty of the evidence (GRADE)**	**Comments**
	**Risk with non-partnered dance**	**Risk with Partnered**				
Time Up and Go Test (in seconds)	The mean time Up and Go Test (in seconds) was 9.6 seconds	MD 3.8 seconds higher (1.45 lower to 9.05 higher)	-	39 (1 RCT)	⊕⊖⊖⊖ VERY LOW [1 2]	
Balance: Berg Balance Scale assessed with: Berg Balance Scale follow up: mean 6 months	The mean balance: Berg Balance Scale was 49 points	MD 0.8 points lower (5.32 lower to 3.72 higher)	-	39 (1 RCT)	⊕⊖⊖⊖ VERY LOW [1 2]	
***The risk in the intervention group** (and its 95% confidence interval) is based on the assumed risk in the comparison group and the **relative effect** of the intervention (and its 95% CI).<br><br>**CI:** Confidence interval; **RR:** Risk ratio; **OR:** Odds ratio;						
**GRADE Working Group grades of evidence**<br>**High certainty:** We are very confident that the true effect lies close to that of the estimate of the effect<br>**Moderate certainty:** We are moderately confident in the effect estimate: The true effect is likely to be close to the estimate of the effect, but there is a possibility that it is substantially different<br>**Low certainty:** Our confidence in the effect estimate is limited: The true effect may be substantially different from the estimate of the effect<br>**Very low certainty:** We have very little confidence in the effect estimate: The true effect is likely to be substantially different from the estimate of effect						

Footnotes

1 The single included study had high risks of selection bias and high risk of bias in blinding of participants and personnel. Certainty of evidence downgraded two levels.

2 A small single included study with wide 95% confidence interval, ranging from substantially lower to substantially higher estimates on either end.

3 Dancing versus different exercises

Table 5 Tango compared to treadmill for people with Parkinson's Disease

**Tango compared to treadmill for people with Parkinson's Disease**					
**Patient or population:** people with Parkinson's Disease<br>**Setting:**<br>**Intervention:** Tango<br>**Comparison:** treadmill					
**Outcomes**	**Anticipated absolute effects* (95% CI)**				**Comments**

	Risk with treadmill	Risk with Tango	Relative effect (95% CI)	№ of participants (studies)	Certainty of the evidence (GRADE)	
Disease severity (MDS-UPDRS III- motor examination) - 3 months	The mean disease severity (MDS-UPDRS III- motor examination) - 3 months was 32.8 points	MD 2.4 points higher (3.41 lower to 8.21 higher)	-	65 (1 RCT)	-	
Disease severity (MDS-UPDRS III- motor examination) - 6 months	The mean disease severity (MDS-UPDRS III- motor examination) - 6 months was 33 points	MD 2 points higher (3.87 lower to 7.87 higher)	-	65 (1 RCT)	⨁⊝⊝⊝ VERY LOW [1,2]	
Balance: Mini-BEST test - 3 months	The mean balance: Mini-BEST test - 3 months was 19 points	MD 0 points (1.82 lower to 1.82 higher)	-	65 (1 RCT)	⨁⊝⊝⊝ VERY LOW [1,2]	
Balance: Mini-BEST test - 6 months	The mean balance: Mini-BEST test - 6 months was 18.8 points	MD 0.7 points higher (1.52 lower to 2.92 higher)	-	65 (1 RCT)	⨁⊝⊝⊝ VERY LOW [1,2]	
Walking distance: six-minute walk test - 3 months	The mean walking distance: six-minute walk test - 3 months was 420 meters	MD 6 meters higher (55.23 lower to 67.23 higher)	-	65 (1 RCT)	⨁⊝⊝⊝ VERY LOW [1,2]	
Walking distance: six-minute walk test - 6 months	The mean walking distance: six-minute walk test - 6 months was 424 meters	MD 2 meters lower (62.99 lower to 58.99 higher)	-	65 (1 RCT)	⨁⊝⊝⊝ VERY LOW [1,2]	
Forward velocity (meter/second) - 3 months	The mean forward velocity (meter/second) - 3 months was 109.5 meters/second	MD 6.5 meters/second lower (19.68 lower to 6.68 higher)	-	65 (1 RCT)	⨁⊝⊝⊝ VERY LOW [1,2]	
Forward velocity (meter/second) - 6 months	The mean forward velocity (meter/second) - 6 months was 110 meters/second	MD 5 meters/second lower (16.79 lower to 6.79 higher)	-	65 (1 RCT)	⨁⊝⊝⊝ VERY LOW [1,2]	
Quality of life (PDQ 39) - 3 months	The mean quality of life (PDQ 39) - 3 months was 18 points	MD 1 points higher (5.23 lower to 7.23 higher)	-	65 (1 RCT)	⨁⊝⊝⊝ VERY LOW [1,2]	
Quality of life (PDQ 39) - 6 months	The mean quality of life (PDQ 39) - 6 months was 17 points	MD 2.4 points higher (3.5 lower to 8.3 higher)	-	65 (1 RCT)	⨁⊝⊝⊝ VERY LOW [1,2]	

***The risk in the intervention group** (and its 95% confidence interval) is based on the assumed risk in the comparison group and the **relative effect** of the intervention (and its 95% CI).

CI: Confidence interval; **RR:** Risk ratio; **OR:** Odds ratio;

GRADE Working Group grades of evidence
High certainty: We are very confident that the true effect lies close to that of the estimate of the effect
Moderate certainty: We are moderately confident in the effect estimate: The true effect is likely to be close to the estimate of the effect, but there is a possibility that it is substantially different
Low certainty: Our confidence in the effect estimate is limited: The true effect may be substantially different from the estimate of the effect
Very low certainty: We have very little confidence in the effect estimate: The true effect is likely to be substantially different from the estimate of effect

Footnotes

[1] The included study had high risk of bias in blinding of participants and personnel, as well as high risk of selection bias.

[2] A small included study with wide 95% confidence interval that ranged from a substantial reduction to a substantial increase in the estimate.

Table 6 Tango compared to stretching and flexibility exercises for people with Parkinson's Disease

Tango compared to stretching/flexibility exercises for people with Parkinson's Disease						
**Patient or population:** people with Parkinson's Disease<br>**Setting:**<br>**Intervention:** Tango<br>**Comparison:** stretching/flexibility exercises						
**Outcomes**	**Anticipated absolute effects[*] (95% CI)**		**Relative effect (95% CI)**	**№ of participants (studies)**	**Certainty of the evidence (GRADE)**	**Comments**
	**Risk with stretching/flexibility exercises**	**Risk with Tango**				
Disease severity (MDS-UPDRS III- motor examination) - 3 months	The mean disease severity (MDS-UPDRS III- motor examination) - 3 months was 33.2 points	MD 2 points higher (3.41 lower to 7.41 higher)	-	59 (1 RCT)	⨁⊝⊝⊝ VERY LOW [1,2]	
Disease severity (MDS-UPDRS III- motor examination) - 6 months	The mean disease severity (MDS-UPDRS III- motor examination) - 6 months ranged from 20.6-36.7 points	MD 1.7 points lower (6.98 lower to 3.58 higher)	-	78 (2 RCTs)	⨁⨁⊝⊝ LOW [1,3]	
Balance: Mini-BEST test - 3 months	The mean balance: Mini-BEST test - 3 months was 19.9 points	MD 0.9 points lower (3.01 lower to 1.21 higher)	-	59 (1 RCT)	⨁⊝⊝⊝ VERY LOW [1,2]	
Balance: Mini-BEST test - 6 months	The mean balance: Mini-BEST test - 6 months was 19.1 points	MD 0.4 points higher (2.27 lower to 3.07 higher)	-	59 (1 RCT)	⨁⊝⊝⊝ VERY LOW [1,2]	
Walking distance: six-minute walk test - 3 months	The mean walking distance: six-minute walk test - 3 months was 440 meters	MD 14 meters lower (83.35 lower to 55.35 higher)	-	59 (1 RCT)	⨁⊝⊝⊝ VERY LOW [1,2]	
Walking distance: six-minute walk test - 6 months	The mean walking distance: six-minute walk test - 6 months was 438 meters	MD 16 meters lower (76.98 lower to 44.98 higher)	-	59 (1 RCT)	⨁⊝⊝⊝ VERY LOW [1,2]	
Forward velocity (meter/second) - 3 months	The mean forward velocity (meter/second) - 3 months was 1.1 meters/second	MD 0.07 meters/second lower (0.2 lower to 0.08 higher)	-	59 (1 RCT)	⨁⊝⊝⊝ VERY LOW [1,2]	
Forward velocity (meter/second) - 6 months	The mean forward velocity (meter/second) - 6 months ranged from 0.9-1.1 meters/second	MD 0.02 meters/second lower (0.08 lower to 0.04 higher)	-	78 (2 RCTs)	⨁⊝⊝⊝ VERY LOW [1,3]	
Quality of life (PDQ 39) - 3 months	The mean quality of life (PDQ 39) - 3 months was 16.2 points	MD 2.8 points higher (3.04 lower to 8.64 higher)	-	59 (1 RCT)	⨁⊝⊝⊝ VERY LOW [1,2]	
Quality of life (PDQ 39) - 6 months	The mean quality of life (PDQ 39) - 6 months was 9.2 points	MD 10.2 points higher (7 higher to 13.4 higher)	-	59 (1 RCT)	⨁⊝⊝⊝ VERY LOW [1,2]	
Balance: Berg Balance Scale<br>Scale from: 0 to 56 (higher better)	The mean balance: Berg Balance Scale was 47.1 points	MD 3.5 points higher (0.88 higher to 6.12 higher)	-	19 (1 RCT)	⨁⨁⊝⊝ LOW [1,2]	
Freezing of Gait Questionnaire<br>Scale from: 0 to 24 (lower better)	The mean freezing of Gait Questionnaire was 6.5 points	MD 0.9 points higher (0.64 lower to 2.44 higher)	-	19 (1 RCT)	⨁⨁⊝⊝ LOW [1,2]	
Timed Up and Go Test	The mean timed Up and Go Test was 11.8 seconds	MD 2 seconds lower (3.12 lower to 0.88 lower)	-	19 (1 RCT)	⨁⨁⊝⊝ LOW [1,2]	

Dual-task walking velocity (meter/second)	The mean dual-task walking velocity (meter/second) was 0.64 meter/second	MD 0.08 meter/second lower (0.33 lower to 0.17 higher)	-	19 (1 RCT)	⊕⊕⊖⊖ LOW [1,2]	
***The risk in the intervention group** (and its 95% confidence interval) is based on the assumed risk in the comparison group and the **relative effect** of the intervention (and its 95% CI).<br><br>**CI:** Confidence interval; **RR:** Risk ratio; **OR:** Odds ratio;						
**GRADE Working Group grades of evidence**<br>**High certainty:** We are very confident that the true effect lies close to that of the estimate of the effect<br>**Moderate certainty:** We are moderately confident in the effect estimate: The true effect is likely to be close to the estimate of the effect, but there is a possibility that it is substantially different<br>**Low certainty:** Our confidence in the effect estimate is limited: The true effect may be substantially different from the estimate of the effect<br>**Very low certainty:** We have very little confidence in the effect estimate: The true effect is likely to be substantially different from the estimate of effect						

Footnotes

[1] The included study/studies had high risks of bias in blinding of participants and personnel, as well as high or unclear risk of selection bias.

[2] A single included study with wide 95% confidence interval

[3] Small number of included studies with wide 95% confidence interval

Table 7 Tango compared to Tai-Chi for people with Parkinson's Disease

**Tango compared to Tai-Chi for people with Parkinson's Disease**						
**Patient or population:** people with Parkinson's Disease<br>**Setting:**<br>**Intervention:** Tango<br>**Comparison:** Tai-Chi						
**Outcomes**	**Anticipated absolute effects* (95% CI)**		**Relative effect (95% CI)**	**№ of participants (studies)**	**Certainty of the evidence (GRADE)**	**Comments**
	**Risk with physiotherapy**	**Risk with Irish dance**				
Quality of life: PDQ 39	The mean quality of life as measured by PDQ 39 was 28.9 points	MD 1.6 points lower (9.65 lower to 6.45 higher)	-	29 (1 RCT)	⊕⊕⊖⊖ LOW [1,2]	
Brief Multidimensional Life Satisfaction Scale (BMLSS)	The mean BMLSS score was 62.3%	MD 3.3 percent higher (9.71 percent lower to 16.31 percent higher)	-	29 (1 RCT)	⊕⊕⊖⊖ LOW [1,2]	
***The risk in the intervention group** (and its 95% confidence interval) is based on the assumed risk in the comparison group and the **relative effect** of the intervention (and its 95% CI).<br><br>**CI:** Confidence interval; **RR:** Risk ratio; **OR:** Odds ratio;						
**GRADE Working Group grades of evidence**<br>**High certainty:** We are very confident that the true effect lies close to that of the estimate of the effect<br>**Moderate certainty:** We are moderately confident in the effect estimate: The true effect is likely to be close to the estimate of the effect, but there is a possibility that it is substantially different<br>**Low certainty:** Our confidence in the effect estimate is limited: The true effect may be substantially different from the estimate of the effect<br>**Very low certainty:** We have very little confidence in the effect estimate: The true effect is likely to be substantially different from the estimate of effect						

Footnotes

[1] The included study had high risk of bias in blinding of participants and personnel, which might have affected the outcome that required subjective evaluation.

[2] A small included study with wide 95% confidence interval ranging from either an important reduction to an important increase in the effect, or substantially different magnitude of effects if they were in the same direction, which was most likely to lead to different clinical decisions based on either side of the confidence interval.

4 Dance versus physiotherapy

Table 8 Irish dance compared to physiotherapy for people with Parkinson's Disease

Outcomes	Anticipated absolute effects* (95% CI)		Relative effect (95% CI)	№ of participants (studies)	Certainty of the evidence (GRADE)	Comments
**Irish dance compared to physiotherapy for people with Parkinson's Disease**						
**Patient or population:** people with Parkinson's Disease<br>**Setting:**<br>**Intervention:** Irish dance<br>**Comparison:** physiotherapy						
	Risk with physiotherapy	Risk with Irish dance				
Disease severity (MDS-UPDRS 3- Motor examination)	The mean disease severity (MDS-UPDRS 3- Motor examination) was 21 points	MD 3.6 points lower (6.42 lower to 0.78 lower)	-	24 (1 RCT)	⊕⊕⊖⊖ LOW 1 2	
Balance: Berg Balance Scale	The mean balance: Berg Balance Scale was 38.9 points	MD 7.2 points higher (0.36 higher to 14.04 higher)	-	24 (1 RCT)	⊕⊕⊖⊖ LOW 1 2	
Feezing of Gait Questionnaire	The mean feezing of Gait Questionnaire was 10.2 points	MD 5.3 points lower (8.11 lower to 2.49 lower)	-	24 (1 RCT)	⊕⊕⊖⊖ LOW 1 2	
Adverse events: fall (any cause) during study period	Study population		RR 0.90 (0.60 to 1.36)	24 (1 RCT)	⊕⊕⊖⊖ LOW 1 2	
	833 per 1,000	750 per 1,000 (500 to 1,000)				
Adverse events: fall during the time of intervention	Study population		RR 3.00 (0.13 to 67.06)	24 (1 RCT)	⊕⊕⊖⊖ LOW 1 2	
	0 per 1,000	0 per 1,000 (0 to 0)				
Quality of life: PDQ 39	The mean quality of life: PDQ 39 was 27.6 points	MD 5.4 points lower (12.63 lower to 1.83 higher)	-	24 (1 RCT)	⊕⊕⊖⊖ LOW 1 2	
***The risk in the intervention group** (and its 95% confidence interval) is based on the assumed risk in the comparison group and the **relative effect** of the intervention (and its 95% CI).<br><br>**CI:** Confidence interval; **RR:** Risk ratio; **OR:** Odds ratio;						
**GRADE Working Group grades of evidence**<br>**High certainty:** We are very confident that the true effect lies close to that of the estimate of the effect<br>**Moderate certainty:** We are moderately confident in the effect estimate: The true effect is likely to be close to the estimate of the effect, but there is a possibility that it is substantially different<br>**Low certainty:** Our confidence in the effect estimate is limited: The true effect may be substantially different from the estimate of the effect<br>**Very low certainty:** We have very little confidence in the effect estimate: The true effect is likely to be substantially different from the estimate of effect						

Footnotes

[1] The included study had high risk of bias in blinding of participants and personnel, which might have affected the outcome that required subjective evaluation.

[2] A small included study with wide 95% confidence interval ranging from either an important reduction to an important increase in the effect, or substantially different magnitude of effects if they were in the same direction, which was most likely to lead to different clinical decisions based on either side of the confidence interval.
