# Supplementary material for: Evidence of disease severity, cognitive and physical outcomes of dance interventions for persons with Parkinson’s Disease: a systematic review and meta-analysis
Source: BMC Geriatr. 2021 Sep 22;21:503. doi: 10.1186/s12877-021-02446-w (PMC8456607; doi:10.1186/s12877-021-02446-w)
Supplement: Supplementary file 6 — Additional file 6. Outcome data for the study with skewed data. [file 12877_2021_2446_MOESM6_ESM.pdf]

Evidence of disease severity, cognitive and physical outcomes of dance interventions for persons with Parkinson’s Disease: a systematic review and meta-analysis

Sophia Rasheeqa Ismail[1*], Shaun Wen Huey Lee[2], Dafna Merom[3], Puteri Sofia Nadira Megat Kamaruddin[1], Min San Chong[4], Terence Ong[4], Nai Ming Lai[2,5]

[1] Institute for Medical Research, National Institutes of Health, Ministry of Health, Malaysia

[2] School of Pharmacy, Monash University Malaysia, Malaysia

[3] University of Western Sydney, Australia

[4] University of Malaya Medical Centre, Malaysia.

[5] School of Medicine, Faculty of Health and Medical Sciences, Taylor's University Malaysia

Additional File 6

Outcome data for the study with skewed data

Study information from Shanahan 2017

Outcome	Dance median	Dance IQR	Dance no	Control median	Control IQR	Control no	p value
Disease severity: MDS-UPDSR 3	12	7-23	20	18	8.5-24	21	0.07
Quality of life (PDQ 39)	11.5	8-22	20	12	10-29.5	21	0.43
Walking distance: six-minute walk test (meters)	385	315-515	20	350	300-480	21	0.33
Balance: Mini-BEST Test	24	22.5-26	20	22.5	18-25	21	0.28
