# Supplementary material for: Evidence of disease severity, cognitive and physical outcomes of dance interventions for persons with Parkinson’s Disease: a systematic review and meta-analysis
Source: BMC Geriatr. 2021 Sep 22;21:503. doi: 10.1186/s12877-021-02446-w (PMC8456607; doi:10.1186/s12877-021-02446-w)
Supplement: Supplementary file 7 — Additional file 7. Summary of all outcome estimates. [file 12877_2021_2446_MOESM7_ESM.pdf]

Evidence of disease severity, cognitive and physical outcomes of dance interventions for persons with Parkinson's Disease: a systematic review and meta-analysis

Sophia Rasheeqa Ismail[1*], Shaun Wen Huey Lee[2], Dafna Merom[3], Puteri Sofia Nadira Megat Kamaruddin[1], Min San Chong[4], Terence Ong[4], Nai Ming Lai[2,5]

[1] Institute for Medical Research, National Institutes of Health, Ministry of Health, Malaysia

[2] School of Pharmacy, Monash University Malaysia, Malaysia

[3] University of Western Sydney, Australia

[4] University of Malaya Medical Centre, Malaysia.

[5] School of Medicine, Faculty of Health and Medical Sciences, Taylor's University Malaysia

Additional File 7

Summary of all outcome estimates

1 Dance versus control

Outcome or Subgroup	Studies	Participants	Statistical Method	Effect Estimate
1.1 Disease severity : MDS-UPDRS I	2	23	Mean Difference (IV, Random, 95% CI)	-3.50 [-18.68, 11.67]
1.2 Disease severity : MDS-UPDRS II	2	23	Mean Difference (IV, Random, 95% CI)	-2.09 [-7.57, 3.40]
1.3 Disease severity : MDS-SPDRS III	7		Mean Difference (IV, Random, 95% CI)	Subtotals only
1.3.1 At 3 months	5	148	Mean Difference (IV, Random, 95% CI)	-691 [-9.97, -3.84]
1.3.2 At 6 months	3	131	Mean Difference (IV, Random, 95% CI)	-7.26 [-11.68, -2.85]
1.3.3 At 12 months	2	62	Mean Difference (IV, Random, 95% CI)	-14.91 [-19.77, -10.05]
1.4 Disease severity : MDS-UPDRS IV-dyskinesia	1	33	Mean Difference (IV, Random, 95% CI)	-0.10 [-0.79, 0.59]
1.5 Disease severity : MDS-UPDRS IV-fluctuation	1	33	Mean Difference (IV, Random, 95% CI)	0.60 [-0.92, 2.12]
1.6 Balance : Mini-Best Test	3	95	Mean Difference (IV, Random, 95% CI)	4.47 [2.29, 6.66]
1.7 Balance : Berg Balance Scale	5		Mean Difference (IV, Random, 95% CI)	Subtotals only
1.7.1 At 2 months	1	41	Mean Difference (IV, Random, 95% CI)	1.10 [-0.81, 3.01]
1.7.2 At 3 months	2	32	Mean Difference (IV, Random, 95% CI)	8.42 [3.68, 13.17]
1.7.3 At 4 months	1	48	Mean Difference (IV, Random, 95% CI)	5.10 [-0.00, 10.20]
1.7.4 At 6 months	1	46	Mean Difference (IV, Random, 95% CI)	-1.60 [-4.54, 1.34]
1.8 Activity specific balance confidence	1	46	Mean Difference (IV, Random, 95% CI)	0.20 [-12.72, 13.12]
1.9 Freezing of gait questionnaire	3	89	Mean Difference (IV, Random, 95% CI)	-0.38 [-2.99; 2.24]
1.10 Timed Up and Go Test (seconds)	7	200	Mean Difference (IV, Random, 95% CI)	--1.16 [-2.17 , -0.15]
1.11 Five Times Sit-to-Stand Test (seconds)	1	19	Mean Difference (IV, Random, 95% CI)	-4.90 [-6.51, -3.29]
1.12 Sit-and-Reach Test (cm)	1	19	Mean Difference (IV, Random, 95% CI)	4.60 [-2.78, 11.98]
1.13 Walking distance : Six-minute-walk test (meters)	4	123	Mean Difference (IV, Random, 95% CI)	57.08 [-54.63, 168.78]
1.13.1 Tango with other dance types	3	104	Mean Difference (IV, Random, 95% CI)	-1.34 [-53.91, 51.24]
1.13.2 Sardinian folk dance	1	19	Mean Difference (IV, Random, 95% CI)	238.80 [157.99, 319.61]
1.14 Forward velocity (meter/second)	1	48	Mean Difference (IV, Random, 95% CI)	0.10 [-0.10, 0.30]
1.15 Standing-start 180 degree turn test (number of steps)	1	46	Mean Difference (IV, Random, 95% CI)	1.30 [-0.38, 2.98]
1.16 Standing-start 180 degree turn test (second)	1	46	Mean Difference (IV, Random, 95% CI)	0.40 [-0.18, 0.98]

1.17 Back Scratch Test (cm)	1	19	Mean Difference (IV, Random, 95% CI)	5.30 [-2.94, 13.54]
1.18 Spinal mouse: inclination score	1	46	Mean Difference (IV, Random, 95% CI)	0.80 [-3.61, 5.21]
1.19 Adverse events: falls during study	1	33	Risk Ratio (M-H, Random, 95% CI)	0.56 [0.11, 2.90]
1.20 Cognitive function: Montreal Cognitive Assessment Scale	2	64	Mean Difference (IV, Random, 95% CI)	0.62 [-0.84, 2.08]
1.21 Activity participation: total current activity participation (Activity Card Sort score)	1	52	Mean Difference (IV, Random, 95% CI)	8.00 [-4.40, 20.40]
1.22 Depressive symptoms: Beck Depression Inventory	3	86	Mean Difference (IV, Random, 95% CI)	0.51 [-4.90, 5.91]
1.23 Apathy : Apathy Scale	1	33	Mean Difference (IV, Random, 95% CI)	1.90 [-1.74, 5.54]
1.24 Fatigue: Krupp Fatigue Severity Scale	2	46	Mean Difference (IV, Random, 95% CI)	-1.46 [-12.14, 9.22]
1.25 Quality of life: PDQ 39	2	77	Mean Difference (IV, Random, 95% CI)	2.25 [-3.69’ 8.19]

2 Different forms of dance interventions

2.1 Dance for Parkinson's disease (D4PD) vs Tango

Outcome or Subgroup	Studies	Participants	Statistical Method	Effect Estimate
2.1 Disease severity : MDS-UPDRS III	1	16	Mean Difference (IV, Random, 95% CI)	6.10 [-7.42, 19.62]
2.2 Agility: Time Up and Go Test (seconds)	1	16	Mean Difference (IV, Random, 95% CI)	-0.30 [-1.63, 1.03]
2.3 Walking distance : Six-minute walk test (meters)	1	16	Mean Difference (IV, Random, 95% CI)	36.30 [-49.15, 121.75]
2.4 Forward velocity (meter/second)	1	16	Mean Difference (IV, Random, 95% CI)	0.10 [-0.15, 0.35]
2.5 Quality of life : PDQ 39	1	16	Mean Difference (IV, Random, 95% CI)	23.50 [-64.83, 111.83]

2.2 Tango versus Mixed dance

Outcome or Subgroup	Studies	Participants	Statistical Method	Effect Estimate
2.2.1 Time Up and Go Test (TUG) seconds	1	18	Mean Difference (IV, Random, 95% CI)	-1.60 [-3.75, 0.55]
2.2.2 Functional Gait Assessment (FGA)	1	18	Mean Difference (IV, Random, 95% CI)	1.60 [-3.39, 6.59]
2.2.3 Freezing gait questionnaire	1	18	Mean Difference (IV, Random, 95% CI)	1.60 [-3.95, 7.15]
2.2.4 Berg Balance Scale (BBS)	1	18	Mean Difference (IV, Fixed, 95% CI)	2.30 [-2.21, 6.81]
2.2.5 Disease severity (measured using MDS-UPDRS 2, motor experiences of daily living)	1	18	Mean Difference (IV, Random, 95% CI)	1.20 [-6.21, 8.61]
2.2.6 Quality of life (PDQ-39)	1	18	Mean Difference (IV, Random, 95% CI)	-8.10 [-18.08, 1.88]

2.3 Partnered versus non-partnered dance

Outcome or Subgroup	Studies	Participants	Statistical Method	Effect Estimate
2.3.1 Balance : Berg Balance Scale	1	39	Mean Difference (IV, Random, 95% CI)	-0.80 [-5.32, 3.72]

2.3.2 Time Up and Go Test (seconds)	1	39	Mean Difference (IV, Random, 95% CI)	3.80 [-1.45, 9.05]

3 Dance versus different exercises

3.1 Tango versus treadmill

Outcome or Subgroup	Studies	Participants	Statistical Method	Effect Estimate
3.1.1 Disease severity : MDS-UPDRS III	1		Mean Difference (IV, Random, 95% CI)	Subtotals only
3.1.1.1 3 months	1	65	Mean Difference (IV, Random, 95% CI)	2.40 [-3.41, 8.21]
3.1.1.2 6 months	1	65	Mean Difference (IV, Random, 95% CI)	2.00 [-3.87, 7.87]
3.1.2 Balance: Mini-BEST test	1		Mean Difference (IV, Random, 95% CI)	Subtotals only
3.2.2.1 3 months	1	65	Mean Difference (IV, Random, 95% CI)	0.00 [-1.82, 1.82]
3.2.2.2 6 months	1	65	Mean Difference (IV, Random, 95% CI)	0.70 [-1.52, 2.92]
3.1.3 Walking distance : Six-minute walk test (meters)	1		Mean Difference (IV, Random, 95% CI)	Subtotals only
3.1.3.1 3 months	1	65	Mean Difference (IV, Random, 95% CI)	6.00 [-55.23, 67.23]
3.1.3.2 6 months	1	65	Mean Difference (IV, Random, 95% CI)	-2.00 [-62.99, 58.99]
3.1.4 Forward velocity (meter/second)	1		Mean Difference (IV, Random, 95% CI)	Subtotals only
3.1.4.1 3 months	1	65	Mean Difference (IV, Random, 95% CI)	-6.50 [-19.68, 6.68]
3.1.4.2 6 months	1	65	Mean Difference (IV, Random, 95% CI)	-5.00 [-16.79, 6.79]
3.1.5 Quality of life : PDQ 39	1		Mean Difference (IV, Random, 95% CI)	Subtotals only
3.1.5.1 3 months	1	65	Mean Difference (IV, Random, 95% CI)	1.00 [-5.23, 7.23]
3.1.5.2 6 months	1	65	Mean Difference (IV, Random, 95% CI)	2.40 [-3.50, 8.30]

3.2 Tango versus stretching or flexibility exercises

Outcome or Subgroup	Studies	Participants	Statistical Method	Effect Estimate
3.2.1 Disease severity : MDS-UPDRS III	2		Mean Difference (IV, Random, 95% CI)	Subtotals only
3.2.1.1 1 3 months	1	59	Mean Difference (IV, Random, 95% CI)	2.00 [-3.41, 7.41]
2.2.1.1.2 6 months	2	78	Mean Difference (IV, Random, 95% CI)	0.71 [-2.75, 4.16]
3.2.2 Balance: Mini-BEST test	1		Mean Difference (IV, Random, 95% CI)	Subtotals only
3.2.2.1 3 months	1	59	Mean Difference (IV, Random, 95% CI)	-0.90 [-3.01, 1.21]
3.2.2.2 6 months	1	59	Mean Difference (IV, Random, 95% CI)	0.40 [-2.27, 3.07]
3.2.2 Balance : Berg Balance Scale	1	19	Mean Difference (IV, Random, 95% CI)	3.50 [0.88, 6.12]
3.2.3 Freezing of gait questionnaire	1	19	Mean Difference (IV, Random, 95% CI)	0.90 [-0.64, 2.44]

3.2.4 Walking distance: Six-minute walk test (meters)	1		Mean Difference (IV, Random, 95% CI)	Subtotals only
3.2.4.1 3 months	1	59	Mean Difference (IV, Random, 95% CI)	-14.00 [-83.35, 55.35]
3.2.4.2 6 months	1	59	Mean Difference (IV, Random, 95% CI)	-16.00 [-76.98, 44.98]
3.2.5 Timed Up and Go Test (seconds)	1	19	Mean Difference (IV, Random, 95% CI)	-2.00 [-3.12, -0.88]
3.2.6 Forward velocity (meter/second)	2		Mean Difference (IV, Random, 95% CI)	Subtotals only
3.2.6.1 3 months	1	59	Mean Difference (IV, Random, 95% CI)	-0.07 [-0.21, 0.07]
3.2.6.2 6 months	2	78	Mean Difference (IV, Random, 95% CI)	-0.02 [-0.08, 0.04]
3.2.7 Dual-task walking velocity (meter/second)	1	19	Mean Difference (IV, Random, 95% CI)	-0.08 [-0.33, 0.17]
3.2.8 Quality of life (PDQ 39)	1		Mean Difference (IV, Random, 95% CI)	Subtotals only
3.2.8.1 3 months	1	59	Mean Difference (IV, Random, 95% CI)	2.80 [-3.04, 8.64]
3.2.8.2 6 months	1	59	Mean Difference (IV, Random, 95% CI)	10.20 [7.00, 13.40]

3.3 Tango versus Tai-Chi

Outcome or Subgroup	Studies	Participants	Statistical Method	Effect Estimate
3.3.1 Quality of life (PDQ-39)	1	29	Mean Difference (IV, Random, 95% CI)	-1.60 [-9.65, 6.45]
3.3.2 Brief Multidimensional Life Satisfaction Scale (BMLSS)	1	29	Mean Difference (IV, Random, 95% CI)	3.30 [-9.71, 16.31]

4 Dance versus physiotherapy

4.1 Irish dance versus physiotherapy

Outcome or Subgroup	Studies	Participants	Statistical Method	Effect Estimate
4.1 Disease severity : MDS-UPDRS III	1	24	Mean Difference (IV, Random, 95% CI)	-3.60 [-6.42, -0.78]
4.2 Balance: Berg Balance Scale	1	24	Mean Difference (IV, Random, 95% CI)	7.20 [0.36, 14.04]
4.3 Freezing of Gait Questionnaire	1	24	Mean Difference (IV, Random, 95% CI)	-5.30 [-8.11, -2.49]
4.4 Adverse events: fall (any cause) during study period	1	24	Risk Ratio (M-H, Random, 95% CI)	0.90 [0.60, 1.36]
4.5 Adverse events: fall during the time of intervention	1	24	Risk Ratio (M-H, Random, 95% CI)	3.00 [0.13, 67.06]
4.6 Quality of life: PDQ 39	1	24	Mean Difference (IV, Random, 95% CI)	-5.40 [-12.63, 1.83]
