# Supplementary material for: Evidence of disease severity, cognitive and physical outcomes of dance interventions for persons with Parkinson’s Disease: a systematic review and meta-analysis
Source: BMC Geriatr. 2021 Sep 22;21:503. doi: 10.1186/s12877-021-02446-w (PMC8456607; doi:10.1186/s12877-021-02446-w)
Supplement: Supplementary file 8 — Additional file 8. Forest plots. [file 12877_2021_2446_MOESM8_ESM.pdf]

Evidence of disease severity, cognitive and physical outcomes of dance interventions for persons with Parkinson's Disease: a systematic review and meta-analysis

Sophia Rasheeqa Ismail[1*], Shaun Wen Huey Lee[2], Dafna Merom[3], Puteri Sofia Nadira Megat Kamaruddin[1], Min San Chong[4], Terence Ong[4], Nai Ming Lai[2,5]

[1] Institute for Medical Research, National Institutes of Health, Ministry of Health, Malaysia

[2] School of Pharmacy, Monash University Malaysia, Malaysia

[3] University of Western Sydney, Australia

[4] University of Malaya Medical Centre, Malaysia.

[5] School of Medicine, Faculty of Health and Medical Sciences, Taylor's University Malaysia

Additional File 8

Forest plots

1. Comparison 1: Dance versus control

Study or Subgroup	Dance Mean	Dance SD	Dance Total	Control Mean	Control SD	Control Total	Weight	Mean Difference IV, Random, 95% CI
Duncan 2014	5.8	4	5	14.8	5.8	5	66.5%	−9.00 [−15.18, −2.82]
Michels 2018	37.7	15.9	9	30.3	16.8	4	33.5%	7.40 [−12.07, 26.87]
**Total (95% CI)**			**14**			**9**	**100.0%**	**−3.50 [−18.68, 11.67]**

Heterogeneity: Tau² = 80.19; Chi² = 2.48, df = 1 (P = 0.12); I² = 60%
Test for overall effect: Z = 0.45 (P = 0.65)

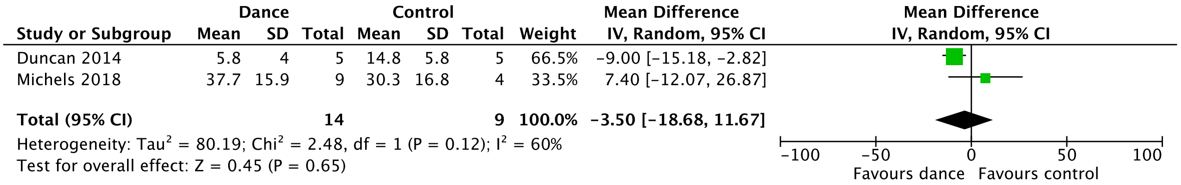

1.1 Disease severity: MDS-UPDRS 1 (non-motor experiences of daily living) (0-16, higher score worse)

Study or Subgroup	Dance Mean	Dance SD	Dance Total	Control Mean	Control SD	Control Total	Weight	Mean Difference IV, Random, 95% CI
Duncan 2014	11.5	7.8	5	17.2	7.6	5	33.1%	−5.70 [−15.25, 3.85]
Michels 2018	10.7	6.5	9	11	5.3	4	66.9%	−0.30 [−7.01, 6.41]
**Total (95% CI)**			**14**			**9**	**100.0%**	**−2.09 [−7.57, 3.40]**

Heterogeneity: Tau² = 0.00; Chi² = 0.82, df = 1 (P = 0.36); I² = 0%
Test for overall effect: Z = 0.74 (P = 0.46)

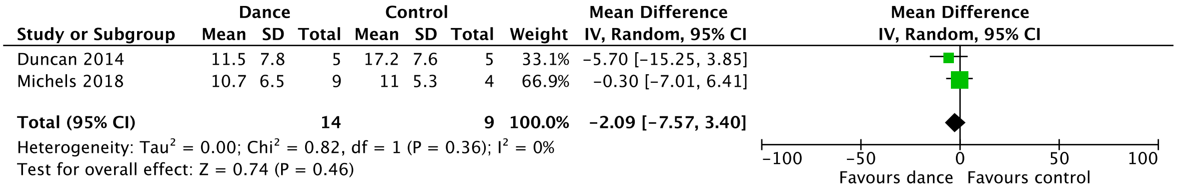

1.2 Disease severity: MDS-UPDRS 2 (motor experiences of daily living) (0-56, higher score worse)

Study or Subgroup	Dance Mean	Dance SD	Dance Total	Control Mean	Control SD	Control Total	Weight	Mean Difference IV, Random, 95% CI
**1.3.1 At 3 months**								
Duncan 2012	39.9	11.7	26	45.6	9.2	26	28.7%	−5.70 [−11.42, 0.02]
McKee 2013	24	7.9	23	29.3	7.5	8	25.1%	−5.30 [−11.42, 0.82]
Michels 2018	23.4	10.6	9	39	12	4	5.0%	−15.60 [−29.25, −1.95]
Rios 2015	19.1	10.2	18	26.3	13.5	15	13.6%	−7.20 [−15.50, 1.10]
Solla 2019	7.7	6.7	10	15.6	6.3	9	27.5%	−7.90 [−13.75, −2.05]
**Subtotal (95% CI)**			**86**			**62**	**100.0%**	**−6.91 [−9.97, −3.84]**
Heterogeneity: Tau² = 0.00; Chi² = 2.11, df = 4 (P = 0.72); I² = 0%								
Test for overall effect: Z = 4.42 (P < 0.0001)								
**1.3.2 At 6 months**								
Duncan 2012	34.2	11.2	26	45.2	9.7	26	35.0%	−11.00 [−16.70, −5.30]
Hackney 2009	25.1	12	31	32.4	10.7	17	29.1%	−7.30 [−13.91, −0.69]
McKee 2013	25.9	7.5	23	29.5	6.7	8	35.9%	−3.60 [−9.16, 1.96]
**Subtotal (95% CI)**			**80**			**51**	**100.0%**	**−7.26 [−11.68, −2.85]**
Heterogeneity: Tau² = 6.05; Chi² = 3.32, df = 2 (P = 0.19); I² = 40%								
Test for overall effect: Z = 3.23 (P = 0.001)								
**1.3.3 At 12 months**								
Duncan 2012	31.7	12.2	26	45	9.7	26	65.8%	−13.30 [−19.29, −7.31]
Duncan 2014	28	6.7	5	46	6.7	5	34.2%	−18.00 [−26.31, −9.69]
**Subtotal (95% CI)**			**31**			**31**	**100.0%**	**−14.91 [−19.77, −10.05]**
Heterogeneity: Tau² = 0.00; Chi² = 0.81, df = 1 (P = 0.37); I² = 0%								
Test for overall effect: Z = 6.01 (P < 0.00001)								

Test for subgroup differences: Chi² = 7.99, df = 2 (P = 0.02), I² = 75.0%

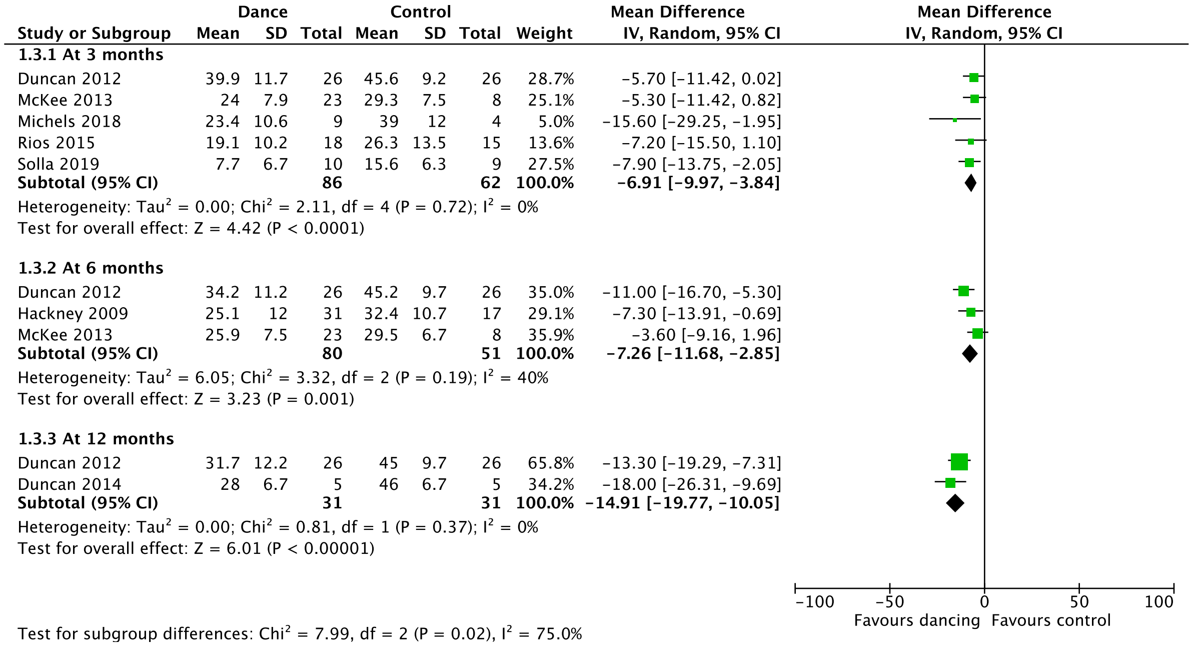

1.3 Disease severity: MDS-UPDRS 3 (Motor examination) (0-108, higher score worse)

Study or Subgroup	Tango Mean	Tango SD	Tango Total	Self-directed exercise Mean	Self-directed exercise SD	Self-directed exercise Total	Weight	Mean Difference IV, Random, 95% CI
Rios 2015	0.6	0.9	18	0.7	1.1	15	100.0%	-0.10 [-0.79, 0.59]
**Total (95% CI)**			**18**			**15**	**100.0%**	**-0.10 [-0.79, 0.59]**

Heterogeneity: Not applicable
Test for overall effect: Z = 0.28 (P = 0.78)

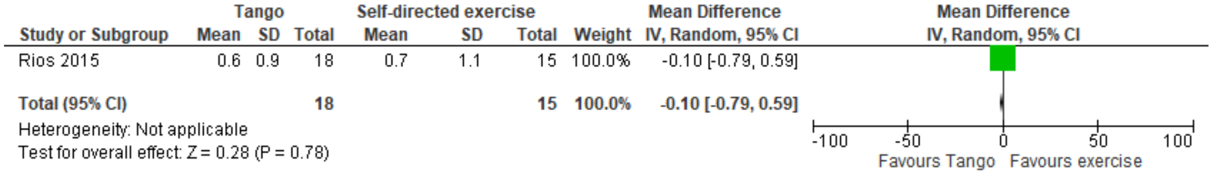

1.4 Disease severity: MDS-UPDRS 4 (dyskinesia) (0-13, higher score worse)

Study or Subgroup	Tango Mean	Tango SD	Tango Total	Self-directed exercise Mean	Self-directed exercise SD	Self-directed exercise Total	Weight	Mean Difference IV, Random, 95% CI
Rios 2015	1.7	2.7	18	1.1	1.7	15	100.0%	0.60 [-0.92, 2.12]
**Total (95% CI)**			**18**			**15**	**100.0%**	**0.60 [-0.92, 2.12]**

Heterogeneity: Not applicable
Test for overall effect: Z = 0.78 (P = 0.44)

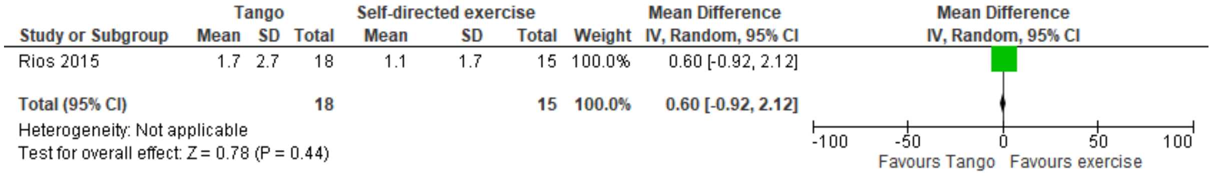

1.5 Disease severity: MDS-UPDRS 4 (fluctuation) (0-10, higher score worse)

Study or Subgroup	Dance Mean	Dance SD	Dance Total	Control Mean	Control SD	Control Total	Weight	Mean Difference IV, Random, 95% CI
Duncan 2012	21.3	5.1	26	17.2	5.6	26	56.2%	4.10 [1.19, 7.01]
Duncan 2014	21.5	2	5	16.7	7.6	5	10.0%	4.80 [-2.09, 11.69]
Rios 2015	36.3	3	18	31.3	6.9	15	33.8%	5.00 [1.24, 8.76]
**Total (95% CI)**			**49**			**46**	**100.0%**	**4.47 [2.29, 6.66]**

Heterogeneity: Tau² = 0.00; Chi² = 0.15, df = 2 (P = 0.93); I² = 0%
Test for overall effect: Z = 4.02 (P < 0.0001)

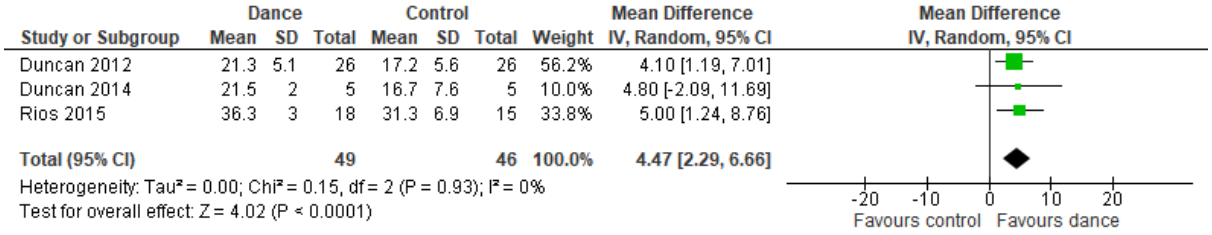

1.6 Balance: Mini-BEST Test (0-28, higher score indicates better balance)

Study or Subgroup	Dance Mean	Dance SD	Dance Total	Control Mean	Control SD	Control Total	Weight	Mean Difference IV, Random, 95% CI
**1.7.1 At 2 months**								
Lee 2018	53.9	2.4	25	52.8	3.4	16	100.0%	1.10 [−0.81, 3.01]
**Subtotal (95% CI)**			**25**			**16**	**100.0%**	**1.10 [−0.81, 3.01]**
Heterogeneity: Not applicable								
Test for overall effect: Z = 1.13 (P = 0.26)								
**1.7.2 At 3 months**								
Michels 2018	54.3	1.7	9	49	6.7	4	37.5%	5.30 [−1.36, 11.96]
Solla 2019	46.9	3.6	10	36.6	6	9	62.5%	10.30 [5.79, 14.81]
**Subtotal (95% CI)**			**19**			**13**	**100.0%**	**8.42 [3.68, 13.17]**
Heterogeneity: Tau² = 4.08; Chi² = 1.48, df = 1 (P = 0.22); I² = 33%								
Test for overall effect: Z = 3.48 (P = 0.0005)								
**1.7.3 At 4 months**								
Hackney 2009	52.1	4.1	31	47	10.3	17	100.0%	5.10 [−0.00, 10.20]
**Subtotal (95% CI)**			**31**			**17**	**100.0%**	**5.10 [−0.00, 10.20]**
Heterogeneity: Not applicable								
Test for overall effect: Z = 1.96 (P = 0.05)								
**1.7.4 At 6 months**								
Kunkel 2017	50.4	5.3	31	52	4.5	15	100.0%	−1.60 [−4.54, 1.34]
**Subtotal (95% CI)**			**31**			**15**	**100.0%**	**−1.60 [−4.54, 1.34]**
Heterogeneity: Not applicable								
Test for overall effect: Z = 1.07 (P = 0.29)								

Test for subgroup differences: Chi² = 14.52, df = 3 (P = 0.002), I² = 79.3%

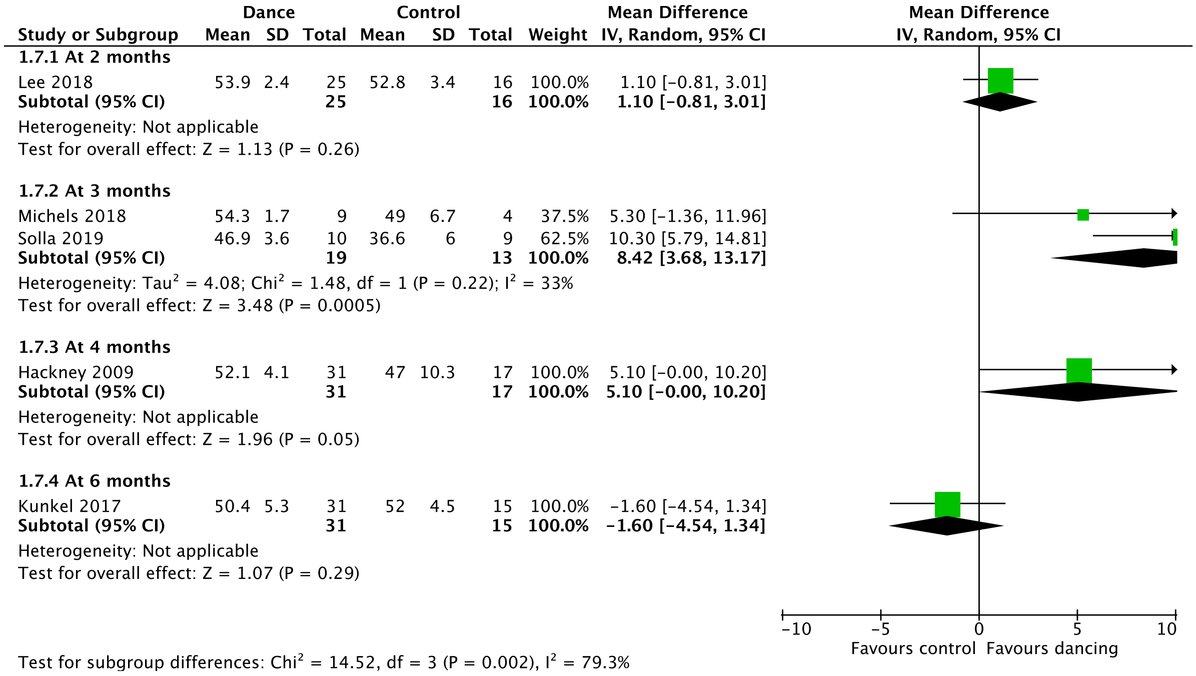

1.7 Balance: Berg Balance Scale (0-56, higher score indicates better balance)

Study or Subgroup	Dance Mean	Dance SD	Dance Total	Control Mean	Control SD	Control Total	Weight	Mean Difference IV, Random, 95% CI
Kunkel 2017	75.6	17.6	31	75.4	22.4	15	100.0%	0.20 [-12.72, 13.12]
**Total (95% CI)**			**31**			**15**	**100.0%**	**0.20 [-12.72, 13.12]**

Heterogeneity: Not applicable
Test for overall effect: Z = 0.03 (P = 0.98)

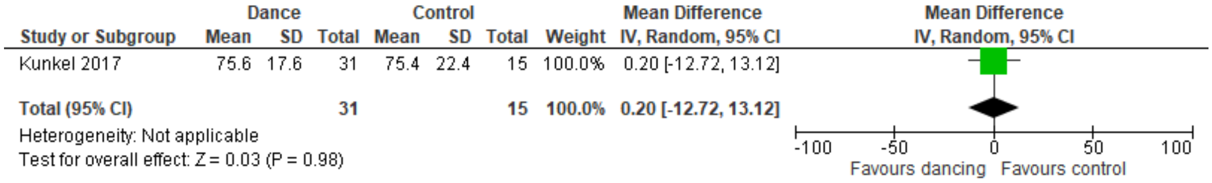

1.8 Balance: Activity-specific balance confidence (0-100, higher score indicates better balance confidence)

Study or Subgroup	Dance Mean	Dance SD	Dance Total	Control Mean	Control SD	Control Total	Weight	Mean Difference IV, Random, 95% CI
Duncan 2014	6.5	5.1	5	8	6.7	5	12.5%	−1.50 [−8.88, 5.88]
Hackney 2009	7.6	4.8	31	5.9	10.3	17	25.5%	1.70 [−3.48, 6.88]
McKee 2013	4.7	4.2	23	5.7	4.1	8	62.0%	−1.00 [−4.32, 2.32]
**Total (95% CI)**			**59**			**30**	**100.0%**	**−0.38 [−2.99, 2.24]**

Heterogeneity: $Tau^2 = 0.00$; $Chi^2 = 0.84$, df = 2 (P = 0.66); $I^2 = 0\%$
Test for overall effect: Z = 0.28 (P = 0.78)

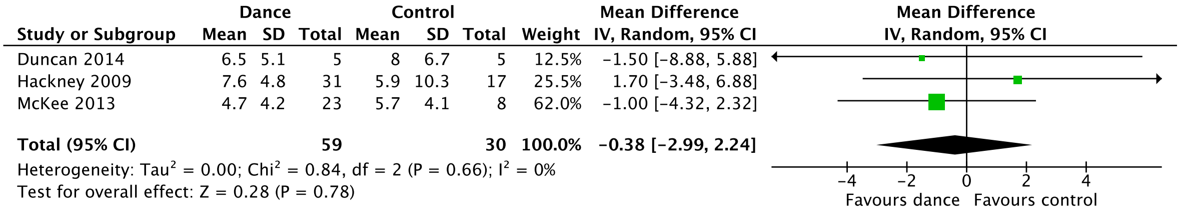

1.9 Freezing of gait questionnaire (0-24, higher score indicates more severe condition)

Study or Subgroup	Dance Mean	Dance SD	Dance Total	Control Mean	Control SD	Control Total	Weight	Mean Difference IV, Random, 95% CI
Duncan 2014	10.9	0.9	5	11.9	1.8	5	17.0%	−1.00 [−2.76, 0.76]
Hackney 2009	10.4	4.1	31	14.4	10.7	17	3.3%	−4.00 [−9.29, 1.29]
Kunkel 2017	13.8	4.4	31	12.3	3.5	15	12.1%	1.50 [−0.85, 3.85]
McKee 2013	10.3	3.2	23	10.5	1.7	8	17.1%	−0.20 [−1.96, 1.56]
Michels 2018	7.8	1.6	9	14.1	10.1	4	1.0%	−6.30 [−16.25, 3.65]
Rios 2015	6.1	1.5	18	8	2.2	15	22.2%	−1.90 [−3.21, −0.59]
Solla 2019	5.1	0.8	10	7	1.2	9	27.3%	−1.90 [−2.83, −0.97]
**Total (95% CI)**			**127**			**73**	**100.0%**	**−1.16 [−2.17, −0.15]**

Heterogeneity: $Tau^2 = 0.74$; $Chi^2 = 11.35$, df = 6 (P = 0.08); $I^2 = 47\%$
Test for overall effect: Z = 2.25 (P = 0.02)

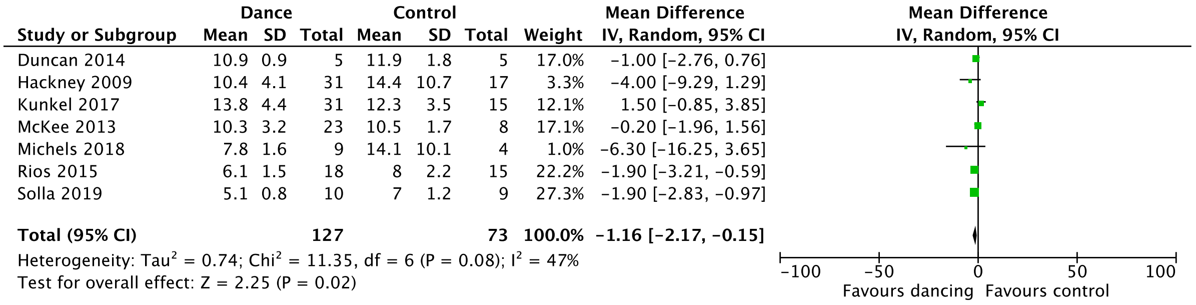

1.10 Timed Up and Go Test (TUG) (seconds)

Study or Subgroup	Dance Mean	Dance SD	Dance Total	Control Mean	Control SD	Control Total	Weight	Mean Difference IV, Random, 95% CI
Solla 2019	6.6	0.6	10	11.5	2.4	9	100.0%	-4.90 [-6.51, -3.29]
**Total (95% CI)**			**10**			**9**	**100.0%**	**-4.90 [-6.51, -3.29]**

Heterogeneity: Not applicable
Test for overall effect: Z = 5.96 (P < 0.00001)

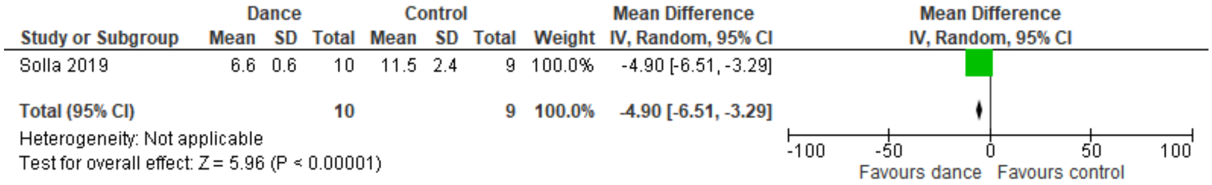

1.11 Five Times Sit-to-Stand Test (seconds) (lower better)

Study or Subgroup	Dance Mean	Dance SD	Dance Total	Control Mean	Control SD	Control Total	Weight	Mean Difference IV, Random, 95% CI
Solla 2019	-1.3	11.1	10	-5.9	4.1	9	100.0%	4.60 [-2.78, 11.98]
**Total (95% CI)**			**10**			**9**	**100.0%**	**4.60 [-2.78, 11.98]**

Heterogeneity: Not applicable
Test for overall effect: Z = 1.22 (P = 0.22)

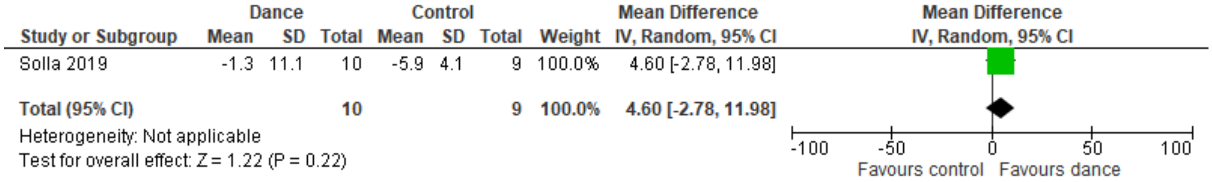

1.12 Sit-and-Reach Test (cm) (longer better)

Study or Subgroup	Dance Mean	Dance SD	Dance Total	Control Mean	Control SD	Control Total	Weight	Mean Difference IV, Random, 95% CI
**1.13.1 Tango with other dance types**								
Duncan 2014	440	67.1	5	470	44.7	5	24.9%	-30.00 [-100.67, 40.67]
Hackney 2009	414.6	36.3	31	360.9	137.3	17	25.2%	53.70 [-12.81, 120.21]
Kunkel 2017	367.3	100.5	31	393.2	90.1	15	25.8%	-25.90 [-83.61, 31.81]
**Subtotal (95% CI)**			**67**			**37**	**75.8%**	**-1.34 [-53.91, 51.24]**
Heterogeneity: $Tau^2 = 1067.67$; $Chi^2 = 3.96$, df = 2 (P = 0.14); $I^2 = 49\%$								
Test for overall effect: Z = 0.05 (P = 0.96)								
**1.13.2 Sardinian folk dance**								
Solla 2019	570.2	76.6	10	331.4	100.1	9	24.2%	238.80 [157.99, 319.61]
**Subtotal (95% CI)**			**10**			**9**	**24.2%**	**238.80 [157.99, 319.61]**
Heterogeneity: Not applicable								
Test for overall effect: Z = 5.79 (P < 0.00001)								
**Total (95% CI)**			**77**			**46**	**100.0%**	**57.08 [-54.63, 168.78]**

Heterogeneity: $Tau^2 = 11745.72$; $Chi^2 = 32.19$, df = 3 (P < 0.00001); $I^2 = 91\%$
Test for overall effect: Z = 1.00 (P = 0.32)
Test for subgroup differences: $Chi^2 = 23.83$, df = 1 (P < 0.00001), $I^2 = 95.8\%$

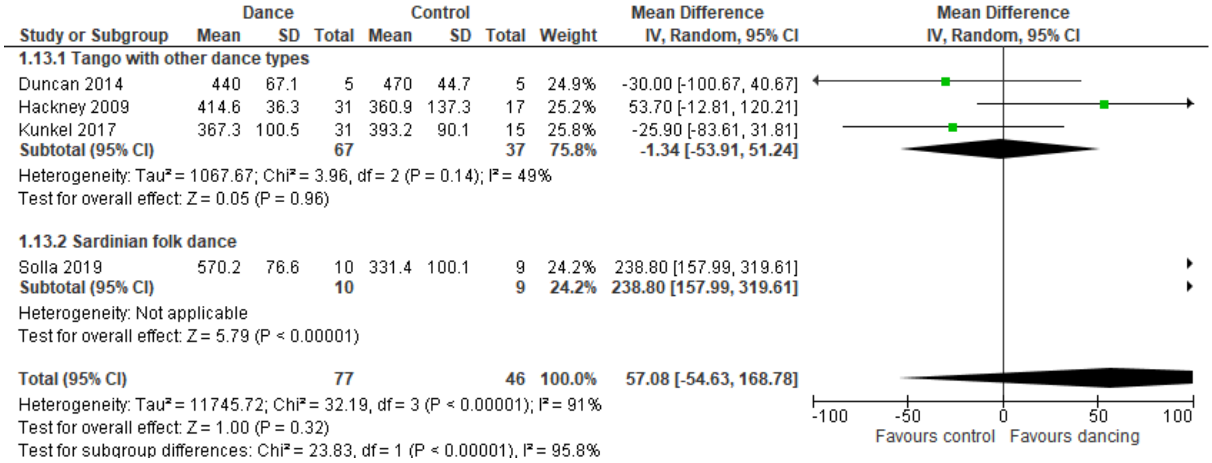

1.13 Walking distance: six-minute walk test (meters) (longer better)

Study or Subgroup	Dance Mean	Dance SD	Dance Total	Control Mean	Control SD	Control Total	Weight	Mean Difference IV, Random, 95% CI
Hackney 2009	1.2	0.2	31	1.1	0.4	17	100.0%	0.10 [-0.10, 0.30]
**Total (95% CI)**			**31**			**17**	**100.0%**	**0.10 [-0.10, 0.30]**

Heterogeneity: Not applicable
Test for overall effect: Z = 0.97 (P = 0.33)

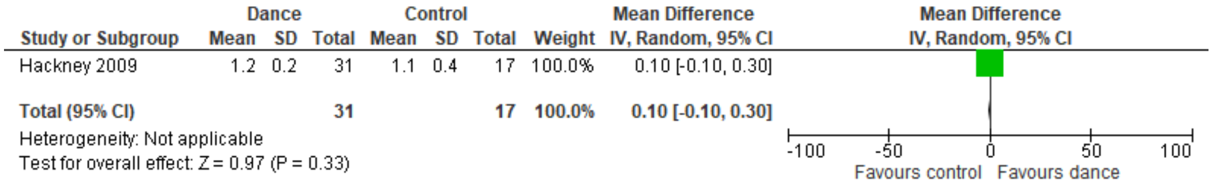

1.14 Forward velocity (meter/second)

Study or Subgroup	Dance Mean	Dance SD	Dance Total	Control Mean	Control SD	Control Total	Weight	Mean Difference IV, Random, 95% CI
Kunkel 2017	5.3	4.6	31	4	0.9	15	100.0%	1.30 [-0.38, 2.98]
**Total (95% CI)**			**31**			**15**	**100.0%**	**1.30 [-0.38, 2.98]**

Heterogeneity: Not applicable
Test for overall effect: Z = 1.51 (P = 0.13)

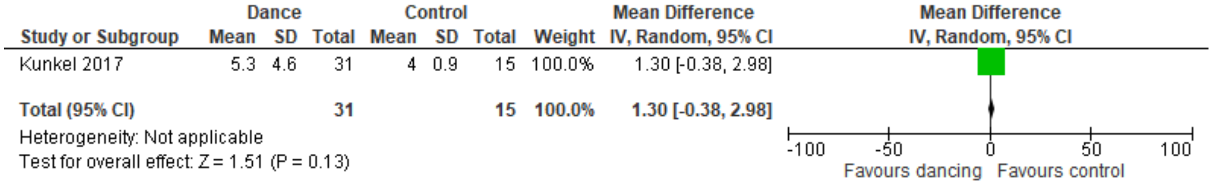

1.15 Standing start 180 degree turn test (number of steps)

Study or Subgroup	Dance Mean	Dance SD	Dance Total	Control Mean	Control SD	Control Total	Weight	Mean Difference IV, Random, 95% CI
Kunkel 2017	2.3	1.3	31	1.9	0.7	15	100.0%	0.40 [-0.18, 0.98]
**Total (95% CI)**			**31**			**15**	**100.0%**	**0.40 [-0.18, 0.98]**

Heterogeneity: Not applicable
Test for overall effect: Z = 1.35 (P = 0.18)

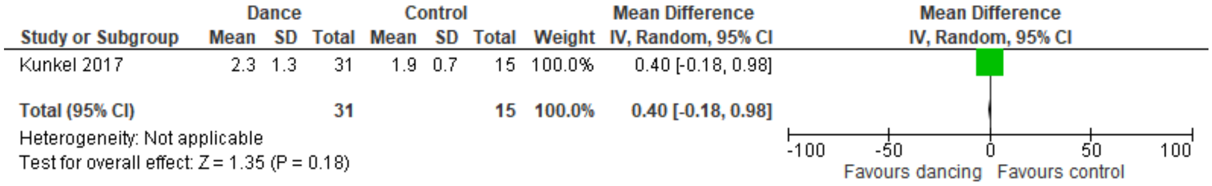

1.16 Standing start 180 degree turn test (time taken in seconds)

Study or Subgroup	Dance Mean	Dance SD	Dance Total	Control Mean	Control SD	Control Total	Weight	Mean Difference IV, Random, 95% CI
Solla 2019	-8.6	8.5	10	-13.9	9.7	9	100.0%	5.30 [-2.94, 13.54]
**Total (95% CI)**			**10**			**9**	**100.0%**	**5.30 [-2.94, 13.54]**

Heterogeneity: Not applicable
Test for overall effect: Z = 1.26 (P = 0.21)

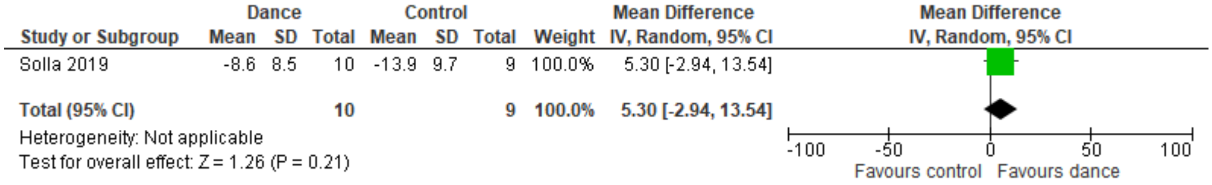

1.17 Back-Scratch Test (cm) (longer better)

Study or Subgroup	Dance Mean	Dance SD	Dance Total	Control Mean	Control SD	Control Total	Weight	Mean Difference IV, Random, 95% CI
Kunkel 2017	8.7	8	31	7.9	6.7	15	100.0%	0.80 [-3.61, 5.21]
**Total (95% CI)**			**31**			**15**	**100.0%**	**0.80 [-3.61, 5.21]**

Heterogeneity: Not applicable
Test for overall effect: Z = 0.36 (P = 0.72)

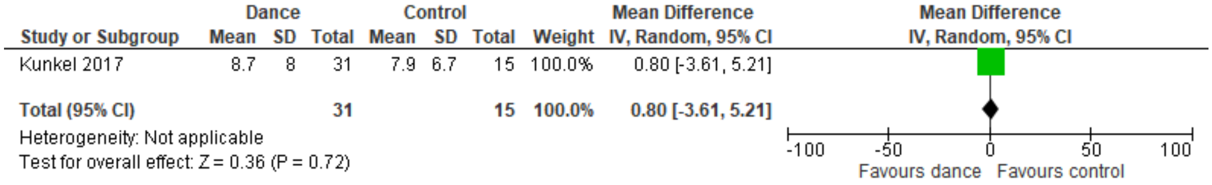

1.18 Spinal mouse inclination degree (higher worse)

Study or Subgroup	Tango Events	Tango Total	Control Events	Control Total	Weight	Risk Ratio M-H, Random, 95% CI
Rios 2015	2	18	3	15	100.0%	0.56 [0.11, 2.90]
**Total (95% CI)**		**18**		**15**	**100.0%**	**0.56 [0.11, 2.90]**
Total events	2		3			

Heterogeneity: Not applicable
Test for overall effect: Z = 0.70 (P = 0.49)

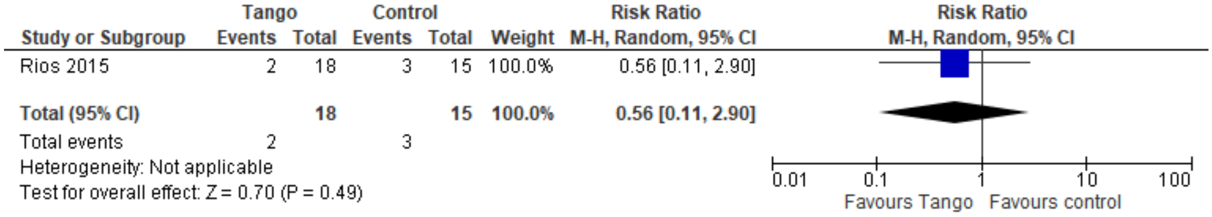

1.19 Adverse events: falls during study

Study or Subgroup	Tango Mean	Tango SD	Tango Total	Control Mean	Control SD	Control Total	Weight	Mean Difference IV, Random, 95% CI
McKee 2013	26.1	3.3	23	26.3	2.3	8	45.4%	−0.20 [−2.29, 1.89]
Rios 2015	27.4	2.1	18	26.1	3.2	15	54.6%	1.30 [−0.59, 3.19]
**Total (95% CI)**			**41**			**23**	**100.0%**	**0.62 [−0.84, 2.08]**

Heterogeneity: Tau² = 0.09; Chi² = 1.09, df = 1 (P = 0.30); I² = 8%
Test for overall effect: Z = 0.83 (P = 0.41)

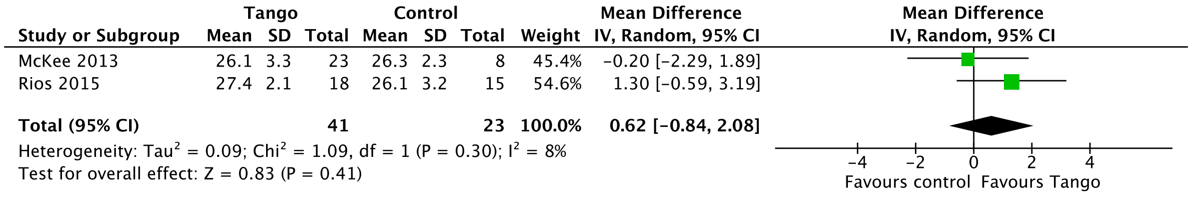

1.20 Cognitive function: Montreal Cognitive Assessment Scale (0-30, higher score better)

Study or Subgroup	Dance Mean	Dance SD	Dance Total	Control Mean	Control SD	Control Total	Weight	Mean Difference IV, Random, 95% CI
Foster 2013	54	30.6	26	46	10.2	26	100.0%	8.00 [-4.40, 20.40]
**Total (95% CI)**			**26**			**26**	**100.0%**	**8.00 [-4.40, 20.40]**

Heterogeneity: Not applicable
Test for overall effect: Z = 1.26 (P = 0.21)

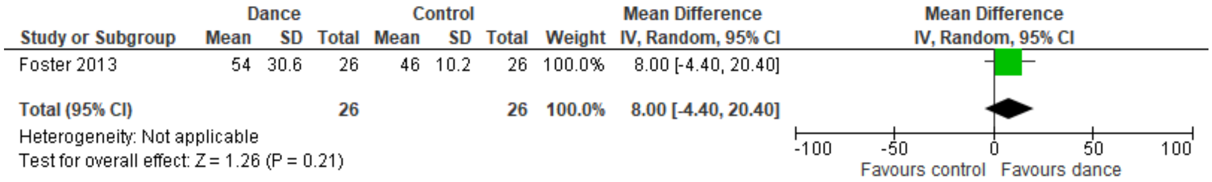

1.21 Activity participation: Activity Card Sort score (0-100, higher score better)

Study or Subgroup	Tango Mean	Tango SD	Tango Total	Control Mean	Control SD	Control Total	Weight	Mean Difference IV, Random, 95% CI
Lee 2018	11.4	7.6	24	16	7.9	16	31.6%	−4.60 [−9.52, 0.32]
Michels 2018	10.9	5.5	9	5.5	2.4	4	33.7%	5.40 [1.11, 9.69]
Rios 2015	7.7	7.2	18	7.3	4.4	15	34.7%	0.40 [−3.60, 4.40]
**Total (95% CI)**			**51**			**35**	**100.0%**	**0.51 [−4.90, 5.91]**

Heterogeneity: Tau² = 17.72; Chi² = 9.07, df = 2 (P = 0.01); I² = 78%
Test for overall effect: Z = 0.18 (P = 0.85)

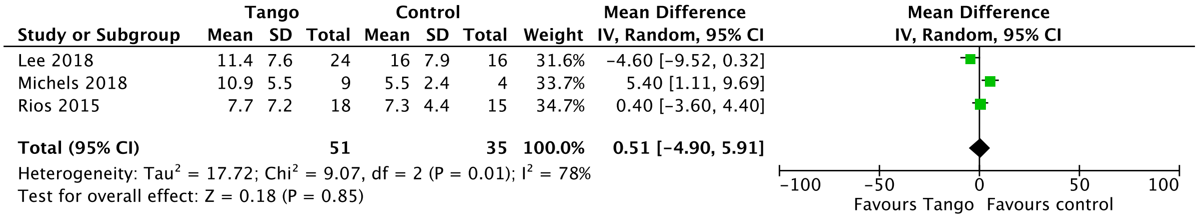

1.22 Depressive symptoms: Beck Depression Inventory score (0-60, higher score worse)

Study or Subgroup	Tango Mean	Tango SD	Tango Total	Control Mean	Control SD	Control Total	Weight	Mean Difference IV, Random, 95% CI
Rios 2015	31.3	4.5	18	29.4	5.9	15	100.0%	1.90 [-1.74, 5.54]
**Total (95% CI)**			**18**			**15**	**100.0%**	**1.90 [-1.74, 5.54]**

Heterogeneity: Not applicable
Test for overall effect: Z = 1.02 (P = 0.31)

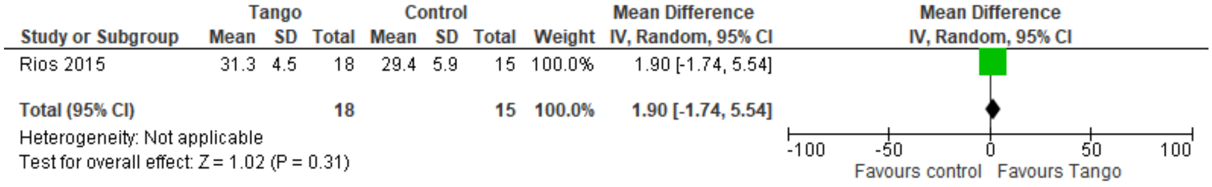

1.23 Apathy: Apathy Scale score (18-72, higher score better, less apathy)

Study or Subgroup	Tango Mean	Tango SD	Tango Total	Control Mean	Control SD	Control Total	Weight	Mean Difference IV, Random, 95% CI
Michels 2018	34.2	14	9	27.5	14.7	4	30.8%	6.70 [−10.36, 23.76]
Rios 2015	30.8	15.3	18	35.9	11.9	15	69.2%	−5.10 [−14.39, 4.19]
**Total (95% CI)**			**27**			**19**	**100.0%**	**−1.46 [−12.14, 9.22]**

Heterogeneity: Tau² = 20.50; Chi² = 1.42, df = 1 (P = 0.23); I² = 29%
Test for overall effect: Z = 0.27 (P = 0.79)

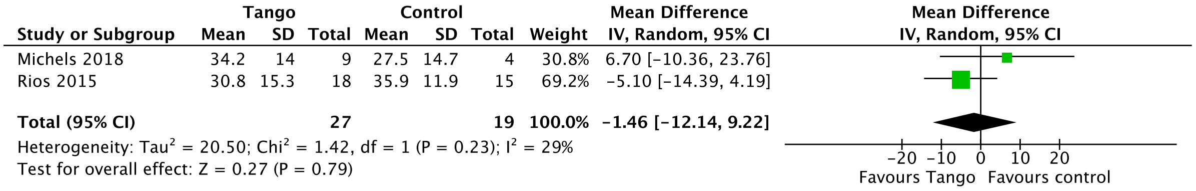

1.24 Fatigue: Krupp Fatigue Severity Scale score (9-63, higher score worse)

Study or Subgroup	Dance Mean	Dance SD	Dance Total	Control Mean	Control SD	Control Total	Weight	Mean Difference IV, Random, 95% CI
Kunkel 2017	21.2	12.1	31	18.5	14.3	15	50.0%	2.70 [−5.70, 11.10]
McKee 2013	19.5	11.1	23	17.7	10.2	8	50.0%	1.80 [−6.60, 10.20]
**Total (95% CI)**			**54**			**23**	**100.0%**	**2.25 [−3.69, 8.19]**

Heterogeneity: Tau² = 0.00; Chi² = 0.02, df = 1 (P = 0.88); I² = 0%
Test for overall effect: Z = 0.74 (P = 0.46)

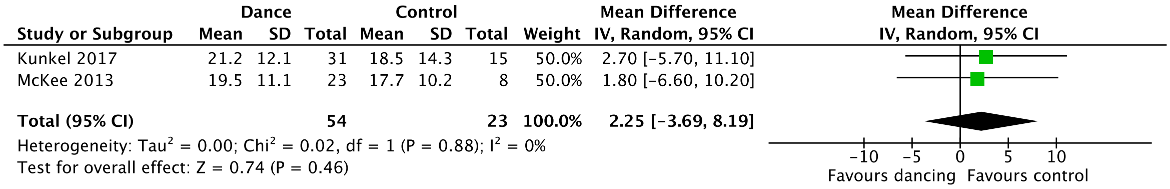

1.25 Quality of life: PDQ 39 score (0-100, higher score worse)

2. Two different forms of dance interventions

2.1. Dance for Parkinson's Disease (D4PD) versus Tango

Study or Subgroup	D4PD Mean	D4PD SD	D4PD Total	Tango Mean	Tango SD	Tango Total	Weight	Mean Difference IV, Random, 95% CI
McNeely 2015	29.4	18.6	8	23.3	5.9	8	100.0%	6.10 [-7.42, 19.62]
**Total (95% CI)**			**8**			**8**	**100.0%**	**6.10 [-7.42, 19.62]**

Heterogeneity: Not applicable
Test for overall effect: Z = 0.88 (P = 0.38)

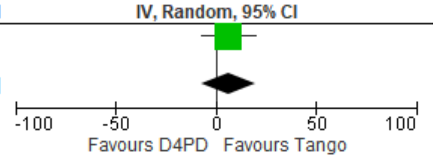

2.1.1. Disease severity: MDS-UPDRS 3 (motor examination) (0-108, higher score worse)

Study or Subgroup	D4PD Mean	D4PD SD	D4PD Total	Tango Mean	Tango SD	Tango Total	Weight	Mean Difference IV, Random, 95% CI
McNeely 2015	9.3	1.2	8	9.6	1.5	8	100.0%	-0.30 [-1.63, 1.03]
**Total (95% CI)**			**8**			**8**	**100.0%**	**-0.30 [-1.63, 1.03]**

Heterogeneity: Not applicable
Test for overall effect: Z = 0.44 (P = 0.66)

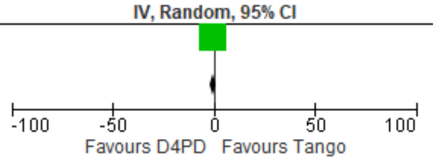

2.1.2. Agility: Timed Up and Go Test (seconds)

Study or Subgroup	D4PD Mean	D4PD SD	D4PD Total	Tango Mean	Tango SD	Tango Total	Weight	Mean Difference IV, Random, 95% CI
McNeely 2015	489.8	58.6	8	453.5	108.5	8	100.0%	36.30 [-49.15, 121.75]
**Total (95% CI)**			**8**			**8**	**100.0%**	**36.30 [-49.15, 121.75]**

Heterogeneity: Not applicable
Test for overall effect: Z = 0.83 (P = 0.41)

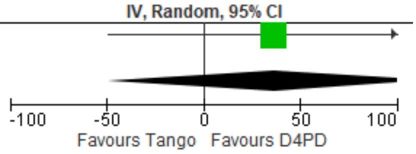

2.1.3. Walking distance: six-minute walk test (meters)

Study or Subgroup	D4PD Mean	D4PD SD	D4PD Total	Tango Mean	Tango SD	Tango Total	Weight	Mean Difference IV, Random, 95% CI
McNeely 2015	1.5	0.27	8	1.4	0.25	8	100.0%	0.10 [-0.15, 0.35]
**Total (95% CI)**			**8**			**8**	**100.0%**	**0.10 [-0.15, 0.35]**

Heterogeneity: Not applicable
Test for overall effect: Z = 0.77 (P = 0.44)

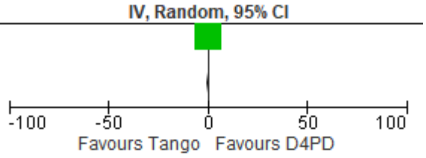

2.1.4. Forward velocity (meter/second)

Study or Subgroup	D4PD Mean	D4PD SD	D4PD Total	Tango Mean	Tango SD	Tango Total	Weight	Mean Difference IV, Random, 95% CI
McNeely 2015	131.1	115	8	107.6	55	8	100.0%	23.50 [-64.83, 111.83]
**Total (95% CI)**			**8**			**8**	**100.0%**	**23.50 [-64.83, 111.83]**

Heterogeneity: Not applicable
Test for overall effect: Z = 0.52 (P = 0.60)

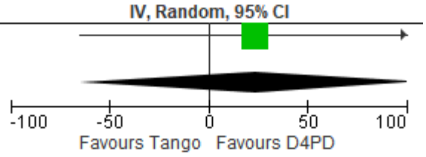

2.1.5. Quality of life: PDQ 39 score (0-100, higher score worse)

2.2. Tango versus Mixed dances

Study or Subgroup	Tango Mean	Tango SD	Tango Total	Mixed dance Mean	Mixed dance SD	Mixed dance Total	Weight	Mean Difference IV, Random, 95% CI
Rocha 2018	8	1.4	8	9.6	3.1	10	100.0%	-1.60 [-3.75, 0.55]
**Total (95% CI)**			**8**			**10**	**100.0%**	**-1.60 [-3.75, 0.55]**
Heterogeneity: Not applicable								
Test for overall effect: Z = 1.46 (P = 0.15)								

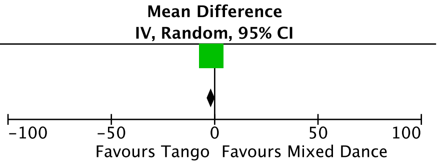

2.2.1. Time Up and Go test (TUG)

Study or Subgroup	Tango Mean	Tango SD	Tango Total	Mixed dance Mean	Mixed dance SD	Mixed dance Total	Weight	Mean Difference IV, Random, 95% CI
Rocha 2018	24.8	4	8	23.2	6.7	10	100.0%	1.60 [-3.39, 6.59]
**Total (95% CI)**			**8**			**10**	**100.0%**	**1.60 [-3.39, 6.59]**
Heterogeneity: Not applicable								
Test for overall effect: Z = 0.63 (P = 0.53)								

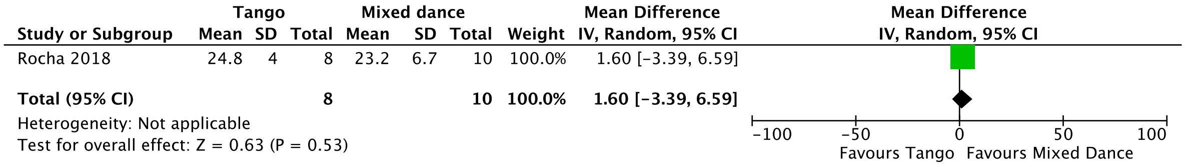

2.2.2. Functional Gait Assessment

Study or Subgroup	Tango Mean	Tango SD	Tango Total	Mixed dance Mean	Mixed dance SD	Mixed dance Total	Weight	Mean Difference IV, Random, 95% CI
Rocha 2018	6.9	6.7	8	5.3	4.9	10	100.0%	1.60 [-3.95, 7.15]
**Total (95% CI)**			**8**			**10**	**100.0%**	**1.60 [-3.95, 7.15]**
Heterogeneity: Not applicable								
Test for overall effect: Z = 0.57 (P = 0.57)								

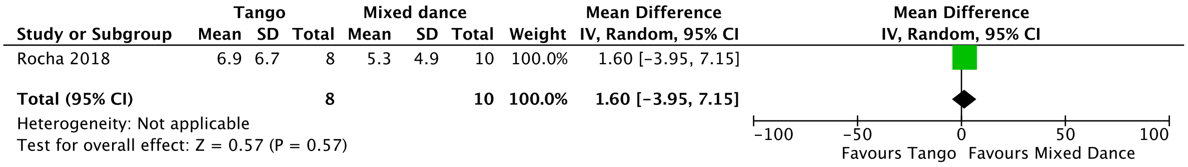

2.2.3. Freezing gait questionnaire (0- 24, lower score better)

Study or Subgroup	Tango Mean	Tango SD	Tango Total	Mixed dance Mean	Mixed dance SD	Mixed dance Total	Weight	Mean Difference IV, Random, 95% CI
Rocha 2018	52.3	3.8	8	50	5.9	10	100.0%	2.30 [-2.21, 6.81]
**Total (95% CI)**			**8**			**10**	**100.0%**	**2.30 [-2.21, 6.81]**
Heterogeneity: Not applicable								
Test for overall effect: Z = 1.00 (P = 0.32)								

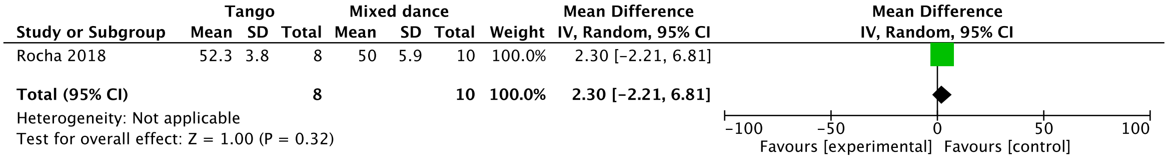

2.2.4. Berg Balance Scale

Study or Subgroup	Tango Mean	Tango SD	Tango Total	Mixed dance Mean	Mixed dance SD	Mixed dance Total	Weight	Mean Difference IV, Random, 95% CI
Rocha 2018	12	9.8	8	10.8	4.8	10	100.0%	1.20 [-6.21, 8.61]
**Total (95% CI)**			**8**			**10**	**100.0%**	**1.20 [-6.21, 8.61]**
Heterogeneity: Not applicable								
Test for overall effect: Z = 0.32 (P = 0.75)								

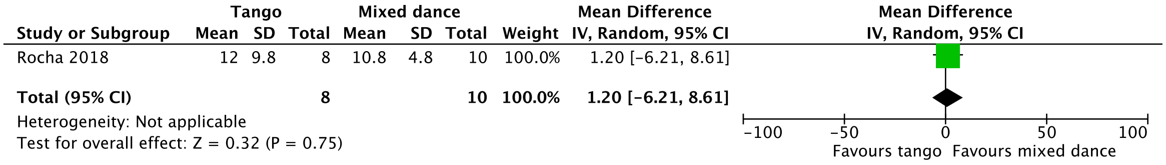

2.2.5. Motor experiences of daily living: MDS-UPDRS 2

Study or Subgroup	Tango Mean	Tango SD	Tango Total	Mixed dance Mean	Mixed dance SD	Mixed dance Total	Weight	Mean Difference IV, Random, 95% CI
Rocha 2018	20.4	12.6	8	28.5	7.8	10	100.0%	-8.10 [-18.08, 1.88]
**Total (95% CI)**			**8**			**10**	**100.0%**	**-8.10 [-18.08, 1.88]**
Heterogeneity: Not applicable								
Test for overall effect: Z = 1.59 (P = 0.11)								

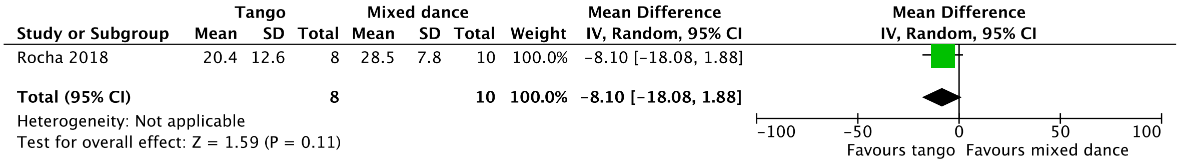

2.2.6. Quality of life: PDQ39

2.3. Partnered versus non-partnered dance

Study or Subgroup	Partnered dance Mean	Partnered dance SD	Partnered dance Total	Non-partnered dance Mean	Non-partnered dance SD	Non-partnered dance Total	Weight	Mean Difference IV, Random, 95% CI
Hackney 2010	48.2	7.8	19	49	6.5	20	100.0%	-0.80 [-5.32, 3.72]
**Total (95% CI)**			**19**			**20**	**100.0%**	**-0.80 [-5.32, 3.72]**
Heterogeneity: Not applicable								
Test for overall effect: Z = 0.35 (P = 0.73)								

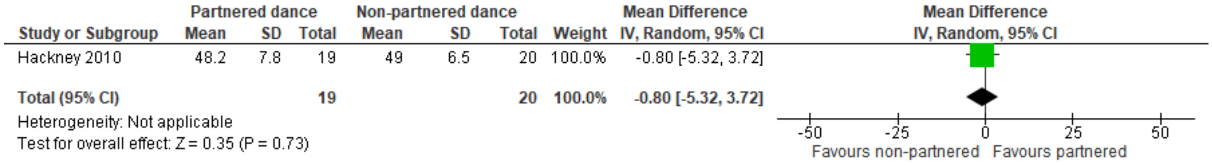

2.3.1. Balance: Berg Balance Scale (0-56, higher score indicates better balance)

Study or Subgroup	Partnered dance Mean	Partnered dance SD	Partnered dance Total	Non-partnered dance Mean	Non-partnered dance SD	Non-partnered dance Total	Weight	Mean Difference IV, Random, 95% CI
Hackney 2010	13.4	11	19	9.6	4	20	100.0%	3.80 [-1.45, 9.05]
**Total (95% CI)**			**19**			**20**	**100.0%**	**3.80 [-1.45, 9.05]**
Heterogeneity: Not applicable								
Test for overall effect: Z = 1.42 (P = 0.16)								

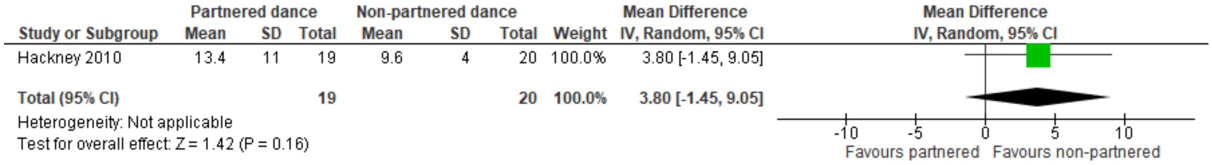

2.3.2. Agility: Timed Up and Go Test (seconds)

3. Dance versus different exercises

3.1. Tango versus treadmill

Study or Subgroup	Tango Mean	Tango SD	Tango Total	Treadmill Mean	Treadmill SD	Treadmill Total	Weight	Mean Difference IV, Random, 95% CI
**4.1.1 3 months**								
Rawson 2019	35.2	12	36	32.8	11.8	29	100.0%	2.40 [-3.41, 8.21]
**Subtotal (95% CI)**			**36**			**29**	**100.0%**	**2.40 [-3.41, 8.21]**
Heterogeneity: Not applicable								
Test for overall effect: Z = 0.81 (P = 0.42)								
**4.1.2 6 months**								
Rawson 2019	35	10.8	36	33	12.9	29	100.0%	2.00 [-3.87, 7.87]
**Subtotal (95% CI)**			**36**			**29**	**100.0%**	**2.00 [-3.87, 7.87]**
Heterogeneity: Not applicable								
Test for overall effect: Z = 0.67 (P = 0.50)								
Test for subgroup differences: Chi² = 0.01, df = 1 (P = 0.92), I² = 0%								

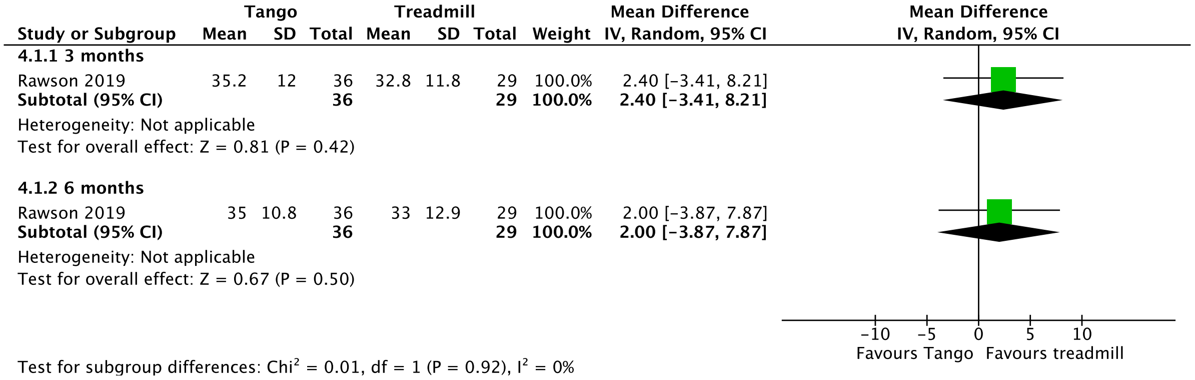

3.1.1. Disease severity: MDS-UPDRS 3 (motor examination) (0-108, higher score worse)

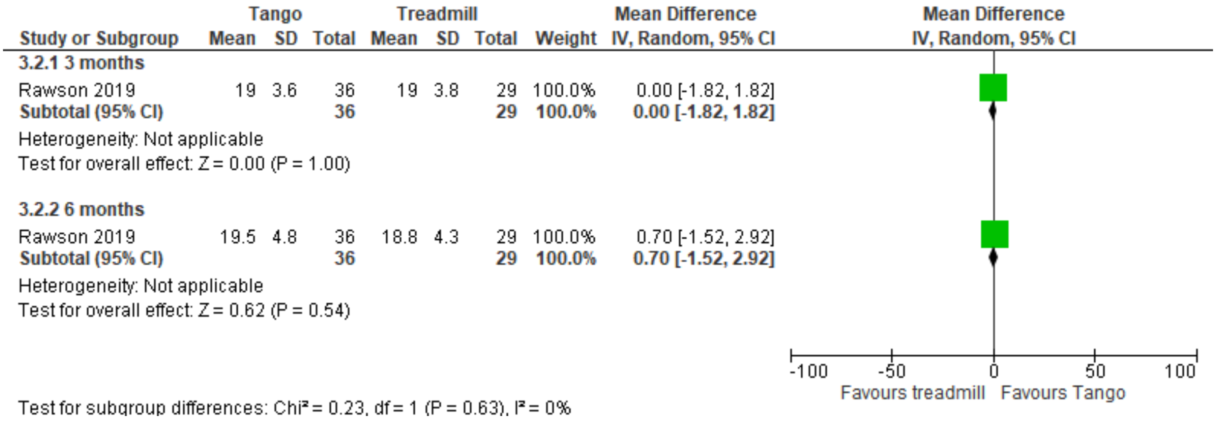

3.1.2. Balance: Mini-BEST Test (0-28, higher score indicates better balance)

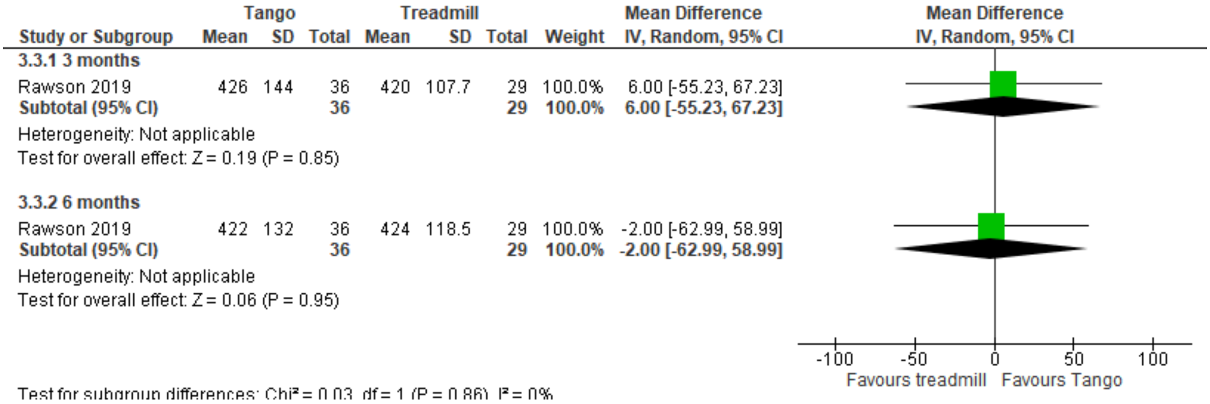

3.1.3. Walking distance: Six-minute walk test (meters)

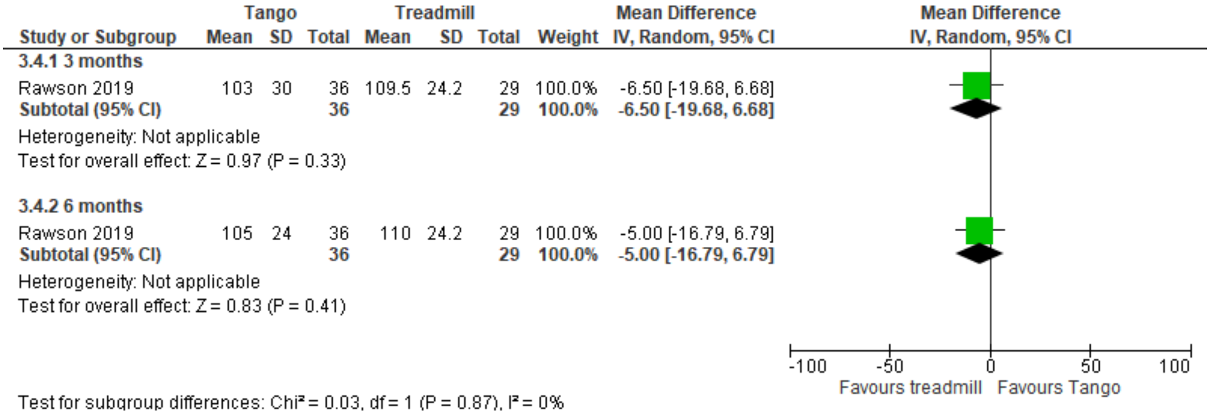

3.1.4. Forward velocity (meter/second)

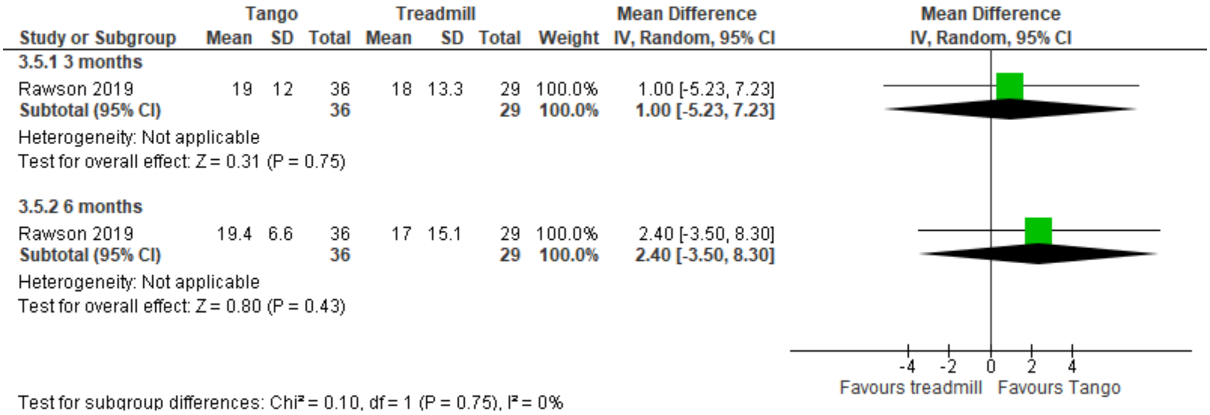

3.1.5. Quality of life: PDQ 39 score (0-100, higher score worse)

3.2. Tango versus stretching or flexibility exercises

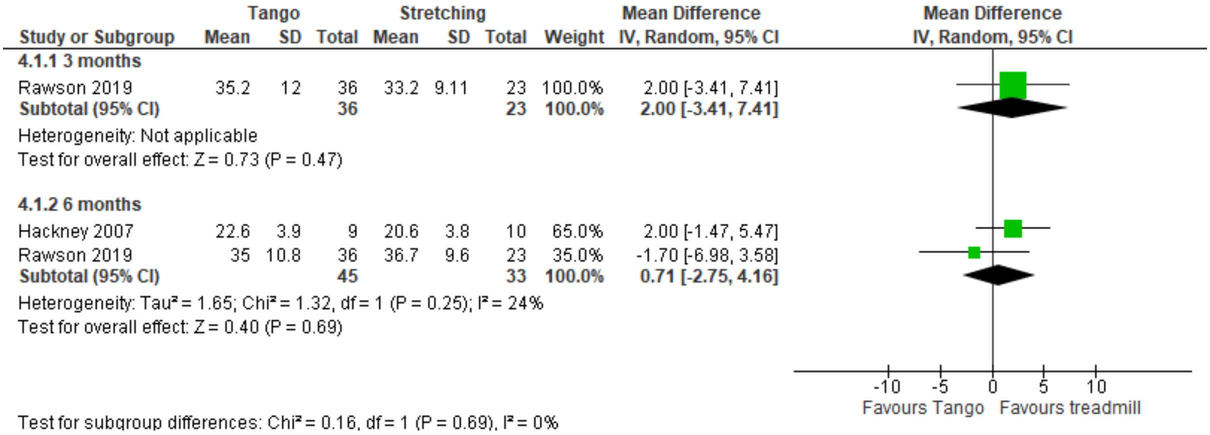

3.2.1. Disease severity: MDS-UPDRS 3 (motor examination) (0-108, higher score worse)

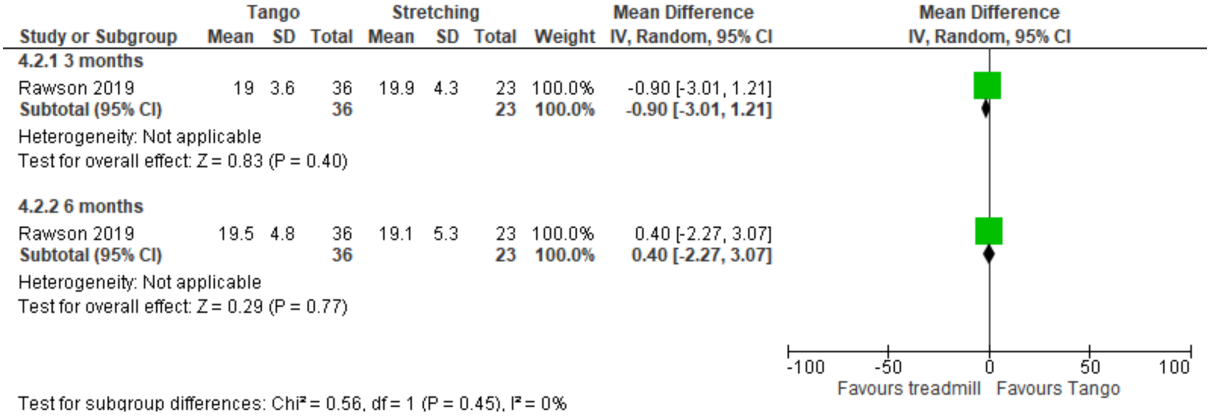

3.2.2. Balance: Mini-BEST Test (0-28, higher score indicates better balance)

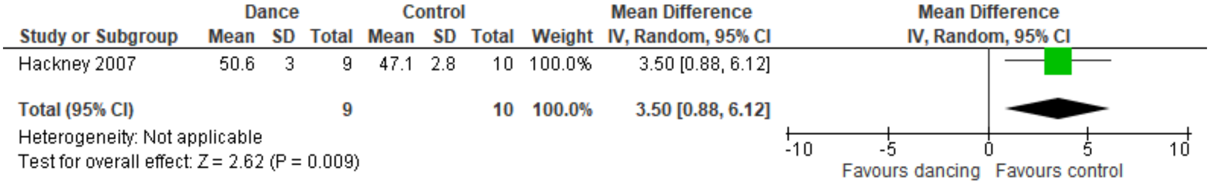

3.2.3. Balance: Berg Balance Scale (0-56, higher score indicates better balance)

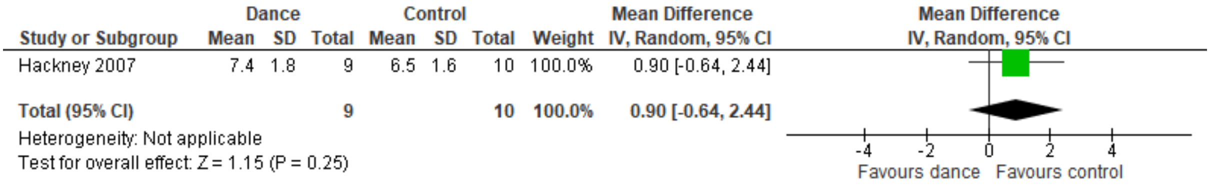

3.2.4. Freezing of gait questionnaire (0-24, higher score indicates more severe condition)

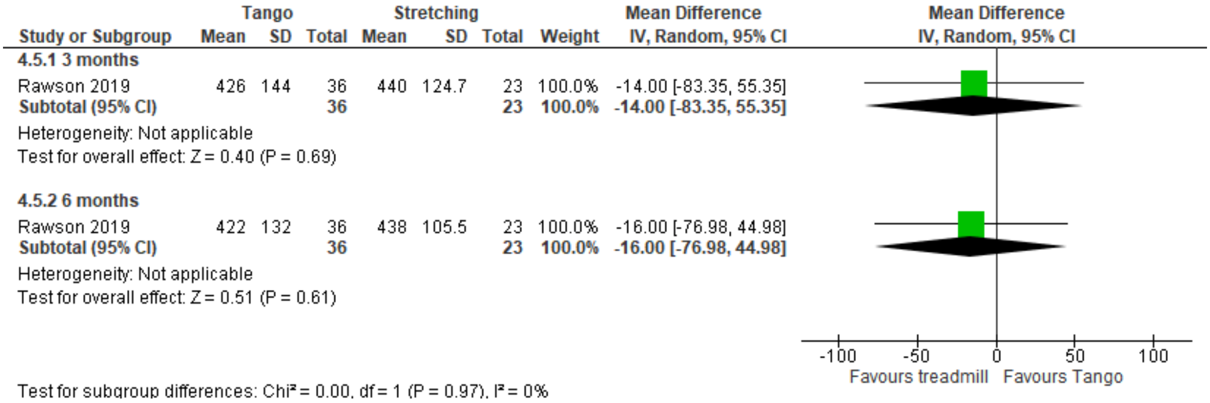

3.2.5. Walking distance: Six-minute walk test (meters)

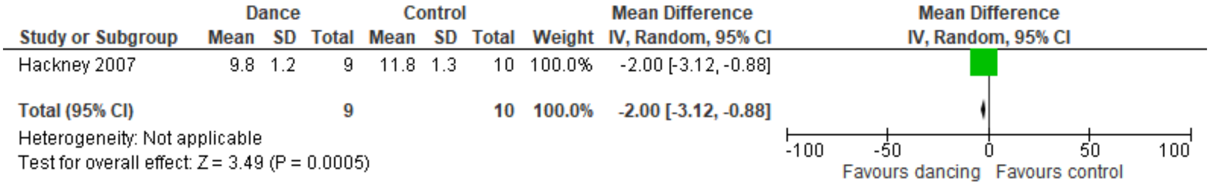

3.2.6. Agility: Timed Up and Go Test (seconds)

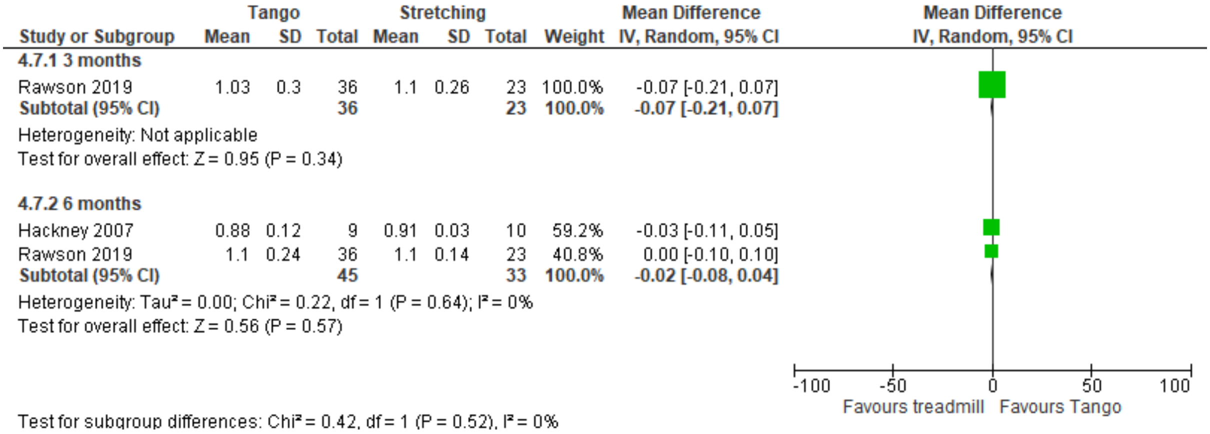

3.2.7. Forward velocity (meter/second)

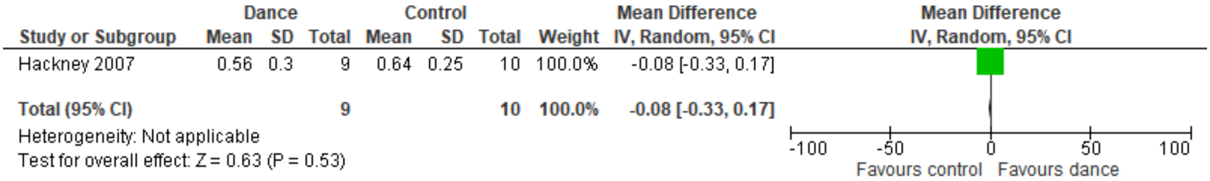

3.2.8. Dual-task walking velocity (meter/second)

Study or Subgroup	Tango Mean	Tango SD	Tango Total	Stretching Mean	Stretching SD	Stretching Total	Weight	Mean Difference IV, Random, 95% CI
**4.9.1 3 months**								
Rawson 2019	19	12	36	16.2	10.6	23	100.0%	2.80 [-3.04, 8.64]
**Subtotal (95% CI)**			**36**			**23**	**100.0%**	**2.80 [-3.04, 8.64]**
Heterogeneity: Not applicable								
Test for overall effect: Z = 0.94 (P = 0.35)								
**4.9.2 6 months**								
Rawson 2019	19.4	6.6	36	9.2	5.8	23	100.0%	10.20 [7.00, 13.40]
**Subtotal (95% CI)**			**36**			**23**	**100.0%**	**10.20 [7.00, 13.40]**
Heterogeneity: Not applicable								
Test for overall effect: Z = 6.24 (P < 0.00001)								

Test for subgroup differences: $Chi^2 = 4.74$, df = 1 (P = 0.03), $I^2 = 78.9\%$

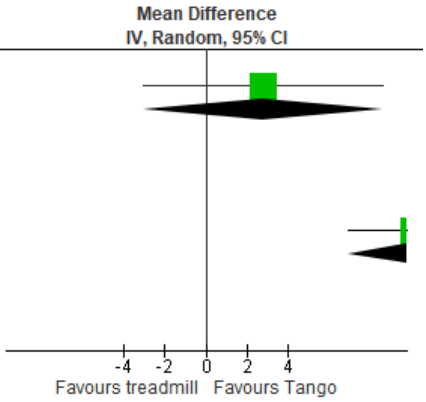

3.2.9. Quality of life: PDQ 39 score (0-100, higher score worse)

3.3. Tango versus Tai-Chi

Study or Subgroup	Tango Mean	Tango SD	Tango Total	Tai-Chi Mean	Tai-Chi SD	Tai-Chi Total	Weight	Mean Difference IV, Random, 95% CI
Poier 2019	27.3	10.9	14	28.9	11.2	15	100.0%	-1.60 [-9.65, 6.45]
**Total (95% CI)**			**14**			**15**	**100.0%**	**-1.60 [-9.65, 6.45]**
Heterogeneity: Not applicable								
Test for overall effect: Z = 0.39 (P = 0.70)								

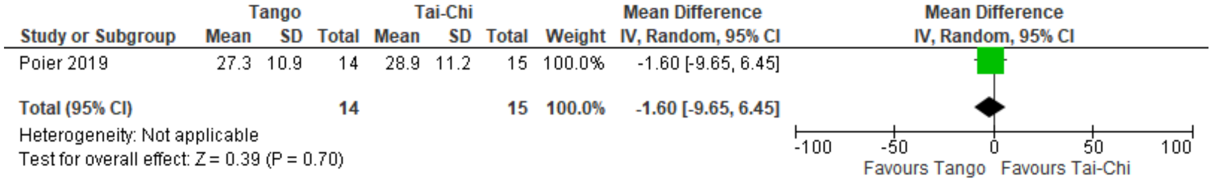

3.3.1. Quality of life: PDQ 39 score (0-100, higher score worse)

Study or Subgroup	Tango Mean	Tango SD	Tango Total	Tai-Chi Mean	Tai-Chi SD	Tai-Chi Total	Weight	Mean Difference IV, Random, 95% CI
Poier 2019	65.6	18.1	14	62.3	17.6	15	100.0%	3.30 [-9.71, 16.31]
**Total (95% CI)**			**14**			**15**	**100.0%**	**3.30 [-9.71, 16.31]**
Heterogeneity: Not applicable								
Test for overall effect: Z = 0.50 (P = 0.62)								

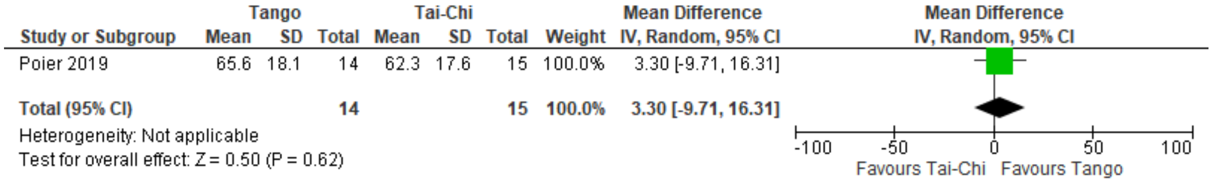

3.3.2. Brief Multidimensional Life Satisfaction Scale (BMLSS) (0-100, higher score better)

4. Dance versus physiotherapy

4.1. Irish Dance versus physiotherapy

Study or Subgroup	Dance Mean	Dance SD	Dance Total	Physiotherapy Mean	Physiotherapy SD	Physiotherapy Total	Weight	Mean Difference IV, Random, 95% CI
Volpe 2013	17.4	3.9	12	21	3.1	12	100.0%	−3.60 [−6.42, −0.78]
**Total (95% CI)**			**12**			**12**	**100.0%**	**−3.60 [−6.42, −0.78]**
Heterogeneity: Not applicable								
Test for overall effect: Z = 2.50 (P = 0.01)								

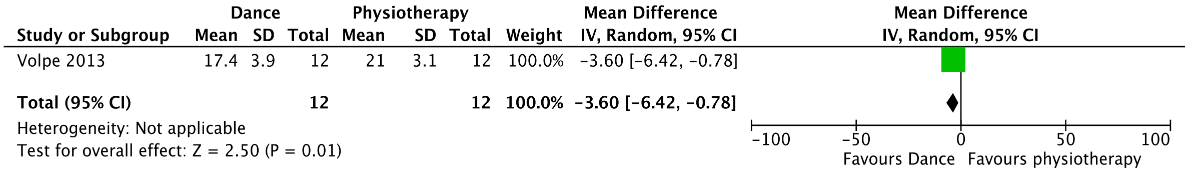

4.1.1. Disease severity: MDS-UPDRS 3 (motor examination) (0-108, higher score worse)

Study or Subgroup	Dance Mean	Dance SD	Dance Total	Physiotherapy Mean	Physiotherapy SD	Physiotherapy Total	Weight	Mean Difference IV, Random, 95% CI
Volpe 2013	46.1	6.8	12	38.9	10	12	100.0%	7.20 [0.36, 14.04]
**Total (95% CI)**			**12**			**12**	**100.0%**	**7.20 [0.36, 14.04]**
Heterogeneity: Not applicable								
Test for overall effect: Z = 2.06 (P = 0.04)								

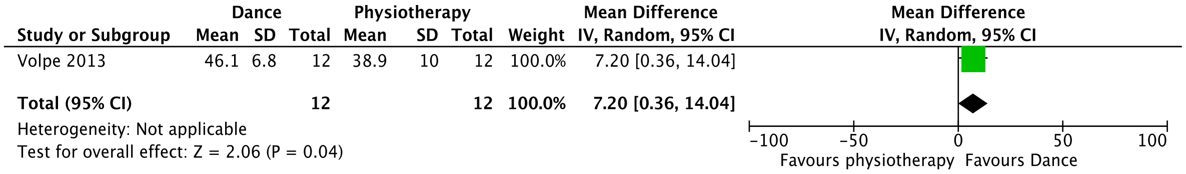

4.1.2. Balance: Berg Balance Scale (0-56, higher score indicates better balance)

Study or Subgroup	Dance Mean	Dance SD	Dance Total	Physiotherapy Mean	Physiotherapy SD	Physiotherapy Total	Weight	Mean Difference IV, Random, 95% CI
Volpe 2013	4.9	2.1	12	10.2	4.5	12	100.0%	-5.30 [-8.11, -2.49]
**Total (95% CI)**			**12**			**12**	**100.0%**	**-5.30 [-8.11, -2.49]**
Heterogeneity: Not applicable								
Test for overall effect: Z = 3.70 (P = 0.0002)								

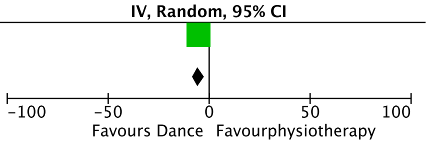

4.1.3. Freezing of gait questionnaire (0-24, higher score indicates more severe condition)

Study or Subgroup	Dance Events	Dance Total	Physiotherapy Events	Physiotherapy Total	Weight	Risk Ratio M-H, Random, 95% CI
Volpe 2013	9	12	10	12	100.0%	0.90 [0.60, 1.36]
**Total (95% CI)**		**12**		**12**	**100.0%**	**0.90 [0.60, 1.36]**
Total events	9		10			
Heterogeneity: Not applicable						
Test for overall effect: Z = 0.50 (P = 0.62)						

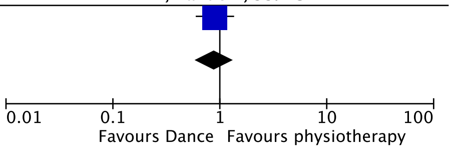

4.1.4. Adverse events: falls (any cause) during study period

Study or Subgroup	Dance Events	Dance Total	Physiotherapy Events	Physiotherapy Total	Weight	Risk Ratio M-H, Random, 95% CI
Volpe 2013	1	12	0	12	100.0%	3.00 [0.13, 67.06]
**Total (95% CI)**		**12**		**12**	**100.0%**	**3.00 [0.13, 67.06]**
Total events	1		0			
Heterogeneity: Not applicable						
Test for overall effect: Z = 0.69 (P = 0.49)						

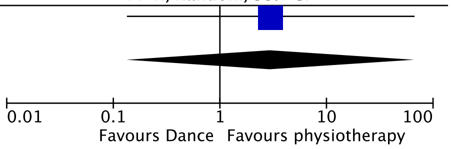

4.1.5. Adverse events: falls during the time of intervention (dancing or physiotherapy)

Study or Subgroup	Dance Mean	Dance SD	Dance Total	Physiotherapy Mean	Physiotherapy SD	Physiotherapy Total	Weight	Mean Difference IV, Random, 95% CI
Volpe 2013	22.2	10.2	12	27.6	7.7	12	100.0%	-5.40 [-12.63, 1.83]
**Total (95% CI)**			**12**			**12**	**100.0%**	**-5.40 [-12.63, 1.83]**
Heterogeneity: Not applicable								
Test for overall effect: Z = 1.46 (P = 0.14)								

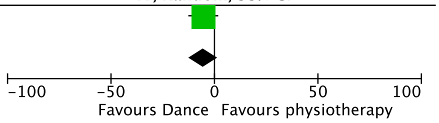

4.1.6. Quality of life: PDQ 39 score (0-100, higher score worse)
