# Supplementary material for: Evidence of disease severity, cognitive and physical outcomes of dance interventions for persons with Parkinson’s Disease: a systematic review and meta-analysis
Source: BMC Geriatr. 2021 Sep 22;21:503. doi: 10.1186/s12877-021-02446-w (PMC8456607; doi:10.1186/s12877-021-02446-w)
Supplement: Supplementary file 9 — Additional file 9. Completed PRISMA checklist for reporting of systematic review. [file 12877_2021_2446_MOESM9_ESM.pdf]

Evidence of disease severity, cognitive and physical outcomes of dance interventions for persons with Parkinson's Disease: a systematic review and meta-analysis

Sophia Rasheeqa Ismail[1*], Shaun Wen Huey Lee[2], Dafna Merom[3], Puteri Sofia Nadira Megat Kamaruddin[1], Min San Chong[4], Terence Ong[4], Nai Ming Lai[2,5]

[1] Institute for Medical Research, National Institutes of Health, Ministry of Health, Malaysia

[2] School of Pharmacy, Monash University Malaysia, Malaysia

[3] University of Western Sydney, Australia

[4] University of Malaya Medical Centre, Malaysia.

[5] School of Medicine, Faculty of Health and Medical Sciences, Taylor's University Malaysia

Additional File 9

Completed PRISMA checklist for reporting of systematic review

Section/topic	#	Checklist item	Reported on page #
**TITLE**			
Title	1	Identify the report as a systematic review, meta-analysis, or both.	1, Title
**ABSTRACT**			
Structured summary	2	Provide a structured summary including, as applicable: background; objectives; data sources; study eligibility criteria, participants, and interventions; study appraisal and synthesis methods; results; limitations; conclusions and implications of key findings; systematic review registration number.	1, Abstract
**INTRODUCTION**			
Rationale	3	Describe the rationale for the review in the context of what is already known.	3-4, background
Objectives	4	Provide an explicit statement of questions being addressed with reference to participants, interventions, comparisons, outcomes, and study design (PICOS).	4-5, objectives
**METHODS**			
Protocol and registration	5	Indicate if a review protocol exists, if and where it can be accessed (e.g., Web address), and, if available, provide registration information including registration number.	5, The systematic review is registered with PROSPERO (CRD42018081017) (https://www.crd.york.ac.uk/prospero/display_record.php?RecordID=81017)
Eligibility criteria	6	Specify study characteristics (e.g., PICOS, length of follow-up) and report characteristics (e.g., years considered, language, publication status) used as criteria for eligibility, giving rationale.	5-6, Methods, type of studies, population, intervention and comparison, outcomes and search strategies.
Information sources	7	Describe all information sources (e.g., databases with dates of coverage, contact with study authors to identify additional studies) in the search and date last searched.	6, Search Strategies.
Search	8	Present full electronic search strategy for at least one database, including any limits used, such that it could be repeated.	6, Search Strategies, Additional File 1, Medline (PubMed) search strategies
Study selection	9	State the process for selecting studies (i.e., screening, eligibility, included in systematic review, and, if applicable, included in the meta-analysis).	7, Selection of studies

Data collection process	10	Describe method of data extraction from reports (e.g., piloted forms, independently, in duplicate) and any processes for obtaining and confirming data from investigators.	7, Data extraction and management, and Additional File 2, Data extraction and management
Data items	11	List and define all variables for which data were sought (e.g., PICOS, funding sources) and any assumptions and simplifications made.	Additional File 2, Data extraction and management.
Risk of bias in individual studies	12	Describe methods used for assessing risk of bias of individual studies (including specification of whether this was done at the study or outcome level), and how this information is to be used in any data synthesis.	7, Assessment of risk of bias, and Additional File 2, Assessment of risk of bias in included studies
Summary measures	13	State the principal summary measures (e.g., risk ratio, difference in means).	7, Meta-analysis, and Additional File 2, Measurement of treatment effects and Data synthesis.
Synthesis of results	14	Describe the methods of handling data and combining results of studies, if done, including measures of consistency (e.g., I^2) for each meta-analysis.	7, Assessment of heterogeneity and meta-analysis, and Additional File 2, Assessment of heterogeneity and Data synthesis.

Section/topic	#	Checklist item	Reported on page #
Risk of bias across studies	15	Specify any assessment of risk of bias that may affect the cumulative evidence (e.g., publication bias, selective reporting within studies).	Additional File 2, Reporting biases
Additional analyses	16	Describe methods of additional analyses (e.g., sensitivity or subgroup analyses, meta-regression), if done, indicating which were pre-specified.	Additional File 2, subgroup analysis and investigation of heterogeneity, and Sensitivity analysis.
**RESULTS**			
Study selection	17	Give numbers of studies screened, assessed for eligibility, and included in the review, with reasons for exclusions at each stage, ideally with a flow diagram.	8, Results, Figure 1(PRISMA flow diagram)
Study characteristics	18	For each study, present characteristics for which data were extracted (e.g., study size, PICOS, follow-up period) and provide the citations.	Table 1, Additional File 3
Risk of bias within studies	19	Present data on risk of bias of each study and, if available, any outcome level assessment (see item 12).	9, Results, Risk of bias in included studies, Figure 2 (risk of bias graph), Figure 3 (Risk of bias summary), Additional File 3, Detailed risk of bias assessment.
Results of individual studies	20	For all outcomes considered (benefits or harms), present, for each study: (a) simple summary data for each intervention group (b) effect estimates and confidence intervals, ideally with a forest plot.	10-17, Effect estimates, Additional File 6, report of all effect estimates in detail, Additional File 7, all forest plots.
Synthesis of results	21	Present results of each meta-analysis done, including confidence intervals and measures of consistency.	10-17, Effect estimates, Additional File 6, report of all effect estimates in detail.

Risk of bias across studies	22	Present results of any assessment of risk of bias across studies (see Item 15).	9, Results, Risk of bias in included studies, Results, effect estimates, GRADE certainty of evidence rating, Additional File 4, Summary of findings table with GRADE certainty of evidence rating.
Additional analysis	23	Give results of additional analyses, if done (e.g., sensitivity or subgroup analyses, meta-regression [see Item 16]).	N/A
**DISCUSSION**			
Summary of evidence	24	Summarize the main findings including the strength of evidence for each main outcome; consider their relevance to key groups (e.g., healthcare providers, users, and policy makers).	18-20, Discussion, paragraphs 1-3.
Limitations	25	Discuss limitations at study and outcome level (e.g., risk of bias), and at review-level (e.g., incomplete retrieval of identified research, reporting bias).	19-20, Discussion, paragraph 3-4.
Conclusions	26	Provide a general interpretation of the results in the context of other evidence, and implications for future research.	20, Conclusions
**FUNDING**			
Funding	27	Describe sources of funding for the systematic review and other support (e.g., supply of data); role of funders for the systematic review.	21, Sources of support.

From: Moher D, Liberati A, Tetzlaff J, Altman DG, The PRISMA Group (2009). Preferred Reporting Items for Systematic Reviews and Meta-Analyses: The PRISMA Statement. PLoS Med 6(7): e1000097. doi:10.1371/journal.pmed1000097

For more information, visit: **www.prisma-statement.org**.
