# Supplementary material for: Evidence of disease severity, cognitive and physical outcomes of dance interventions for persons with Parkinson’s Disease: a systematic review and meta-analysis
Source: BMC Geriatr. 2021 Sep 22;21:503. doi: 10.1186/s12877-021-02446-w (PMC8456607; doi:10.1186/s12877-021-02446-w)
Supplement: Supplementary file 10 — Additional file 10: Table S1. Characteristics of included studies [file 12877_2021_2446_MOESM10_ESM.pdf]

Evidence of disease severity, cognitive and physical outcomes of dance interventions for persons with Parkinson's Disease: a systematic review and meta-analysis

Sophia Rasheeqa Ismail[1*], Shaun Wen Huey Lee[2], Dafna Merom[3], Puteri Sofia Nadira Megat Kamaruddin[1], Min San Chong[4], Terence Ong[4], Nai Ming Lai[2,5]

[1] Institute for Medical Research, National Institutes of Health, Ministry of Health, Malaysia

[2] School of Pharmacy, Monash University Malaysia, Malaysia

[3] University of Western Sydney, Australia

[4] University of Malaya Medical Centre, Malaysia.

[5] School of Medicine, Faculty of Health and Medical Sciences, Taylor's University Malaysia

Additional Table 1

Characteristics of included studies

Study ID (first author and year)	Design, country and total participants	Population and setting	Intervention	Comparison	Outcome
Duncan 2012	RCT, US, n=62	Inclusion criteria: Clinically defined “definite PD” (Hoehn& Yahr Stages I-IV)<br>Exclusion criteria: 1) serious medical condition, 2) evidence of abnormality other than PD related changes on brain imaging (previously done for clinical evaluations), 3) history or evidence of neurological deficit other than PD, or 4) history or evidence of musculoskeletal problem.	One hour twice weekly community-based Argentine Tango classes for 12 months.	No prescribed exercise and were instructed to go about their lives as usual.	Assessment of outcomes at baseline and 12 months.<br>1. Disease Severity: MDS-UPDRS sections 1-3 (higher scores indicate greater disease severity).<br>2. Balance: MiniBESTest (lower scores indicate greater deficits in balance).<br>3. Gait: Freezing of Gait Questionnaire (FOGQ) for freezing gait, Six Minute Walk Test (6MWT) for walking endurance, walking velocity.<br>4. Upper Extremity Function: Nine Hole Peg Test (9HPT)
Duncan 2014	RCT, US, n=10	Inclusion criteria: "Definite" idiopathic Parkinson's Disease on levodopa, aged older than 40 years old Control participants were matched by age, sex, and MDS-UPDRS III score to AT participants<br>Exclusion criteria: (1) a serious medical condition, (2) history or evidence of neurologic deficit other than PD, (3) evidence of brain abnormality other than PD-related changes on brain imaging (previously done, not a part of this study), or (4) history or evidence of a musculoskeletal problem that limited movement.	Argentine Tango (AT)- twice-weekly, 1-hour community-based sessions.<br>Duration: 24 months	No prescribed exercise. Required to maintain their current levels of physical activity during the study.	Assessment of outcomes at baseline, 12 months and 24 months<br>1. Disease Severity: MDS-UPDRS sections 1-3 (higher scores indicate greater disease severity).<br>2. Balance: MiniBESTest (lower scores indicate greater deficits in balance).<br>3. Gait: Freezing of Gait Questionnaire (FOGQ) for freezing gait, Six Minute Walk Test (6MWT) for walking endurance, walking velocity.
Foster 2013	RCT, US, n=62	Inclusion criteria:<br>1. PD classified as Hoehn and Yahr stages I–IV 31, and experienced clear motor benefit from levodopa.<br>2. Participants had to be able to walk independently for 10 feet with or without an assistive device<br>Exclusion criteria:<br>1. History of neurological deficit other than PD<br>2. Serious medical problem(s)<br>3. Evidence of abnormality other than PD-related changes on brain imaging<br>4. History or evidence of musculoskeletal or psychological problems	Intervention: Progressive Argentine tango lessons - one-hour dance classes two times per week for 12 months.<br>Duration of intervention: 12 months	Participants were asked to continue the normal life routine that they had engaged in before enrolling in the study.	Assessment of outcomes at 3, 6 and 12 months.<br>Primary Outcome:<br>1. Participation - Activity Card Sort (ACS) - perceived level of participation in daily life activities as well as changes in participation in relation to certain events (e.g., the onset of disease or disability, beginning a new treatment regimen) or over specified periods of time (e.g., in the past five years) (higher score indicates more active participation)<br>Secondary Outcomes:<br>1. Motor function - Unified Parkinson's Disease Rating Scale (Sections 1-3) (higher scores indicate greater disease severity).

Study ID (first author and year)	Design, country and total participants	Population and setting	Intervention	Comparison	Outcome
					2. Depressive symptoms - Beck Depression Inventory II (higher score indicates more severe depressive symptoms).
Hackney 2007	RCT, US, n=19	Diagnosis: idiopathic "definite" PD<br>Inclusion criteria: Each subject also demonstrated clear benefit from PD medications.<br>Exclusion criteria: not stated.	Intervention group: Progressive tango dance lessons<br>- two one-hour sessions per week for a total of 20 sessions completed within 13 weeks<br>- Structured strength/flexibility exercise classes designed for people with PD and/or elderly individuals.<br>- two one-hour sessions per week for a total of 20 sessions completed within 13 weeks.	Flexibility exercise	Assessment of outcomes at baseline and after 20th training session<br>1. UPDRS, Motor Subscale 3 (higher score indicates more severe disease)<br>2. Balance: Berg Balance Scale (higher score indicates better balance)<br>3. Gait velocity: 5-m path with and without a concurrent dual task<br>4. Agility: Timed Up and Go (TUG)<br>5. Freezing of gait: Freezing of Gait questionnaire (higher score indicates more severe condition).
Hackney 2009	RCT, US, n=58	Inclusion criteria:<br>1. At least 40 years of age<br>2. Ability to stand for at least 30 min<br>3. Ability to walk independently for ≥ 3 m with or without an assistive device<br>4. PD with Hoehn and Yahr (H&Y) stages I–III<br>5. Demonstrated clear benefit from levodopa and were tested on medications at a standardized time to reduce the effects of medication-related fluctuations in performance.<br>Exclusion criteria:<br>1. History of neurological deficit other than PD.	Intervention group 1: Waltz/foxtrot dance lessons<br>Intervention group 2: Tango dance lessons<br>Duration of intervention: 20 completed sessions in 13 weeks	No dance lesson	Assessment of outcomes during the week prior to the initiation of training lessons and during the week of completion of the 20th training lesson.<br>1. UPDRS, Motor Subscale 3 (higher score indicates more severe disease).<br>2. Berg Balance Scale (higher score indicates better balance)<br>3. Gait velocity: 5-m path with and without a concurrent dual task<br>4. Agility: Timed Up and Go (TUG)<br>5. Freezing of Gait questionnaire (higher score indicates more severe condition).<br>6. Walking distance: 6-minute walk test (6MWT)<br>7. Forward and backward gait: 5 m instrumented, computerized GAITRite walkway (CIR Systems, Inc., Havertown, PA, USA).
Hackney 2009b	RCT, US,n=75	Diagnosis: Idiopathic PD (Hoehn and Yahr stages of I-III)<br>N: 75<br>Age:<br>1. Waltz/Foxtrot: mean 66.8 (SE2.4) years<br>2. Tango: mean 68.2 (SE 1.4) years<br>3. Tai Chi: mean 64.9 (SE 2.3) years<br>4. No intervention: mean 66.5 (SE 2.8) years<br>Sex:<br>1. Waltz/Foxtrot: Male 11 / Female 6<br>2. Tango: Male 11 / Female 3<br>3. Tai Chi: Male 11 / Female 2	Duration of intervention: 13 weeks<br>Intervention timing: 20 twice weekly one-hour sessions<br>Intervention group 1: Waltz/Foxtrot (n=19)<br>Intervention group 2: Tango (n=19)<br>Intervention group 3: Tai Chi (n=17)	Control group: No intervention (n=20)	**1.** Health Related Quality of Life (HRQoL)

Study ID (first author and year)	Design, country and total participants	Population and setting	Intervention	Comparison	Outcome
		4. No intervention: Male 12 / Female 5<br>Inclusion criteria:<br>1. At least 40 years of age<br>2. Could stand for at least 30 minutes<br>3. Walk independently 3 or more meters with or without an assistive device<br>Exclusion criteria:<br>1. history of neurological deficit other than PD<br>2. participants received alterations in their medication schedules or doses			
Hackney 2010	RCT, US, n=39	Diagnosis: idiopathic "definite" PD (Hoehn and Yahr [H&Y] stages I-III)<br>Other inclusion criteria:<br>1. No history of other neurological deficits<br>2. At least 40 years of age<br>3. Able to stand for at least 30 minutes<br>4. Able to walk independently for 3 or more meters with or without an assistive device<br>5. Demonstrated clear benefit from levodopa<br><br>Exclusion criteria: a history of other neurological deficits.	Partnered Tango dance<br>Duration of intervention: 10 weeks, twice per week.	Non-partnered Tango dance	1. Berg Balance Scale (BBS) (higher score indicates better balance).<br>2. Timed Up and Go test<br>3. 6-minute walk test. Comfortable and fast-as-possible gait were assessed along a 5-m instrumented,<br>4. Computerized GAITRite walkway
Hulbert 2017	RCT, UK, n=27	Diagnosis: Parkinson's disease (Hoehn and Yahr (H & Y) scale 1–3)<br>Exclusion criteria:<br>1. Not able to follow commands or remember instructions<br>2. Had uncorrected visual or hearing impairments<br>3. Unable to tolerate 90 min of data collection<br>4. Had other concurrent neurological conditions affecting their physical performance	Twice-weekly partnered dance classes of 1 hour, for 10 weeks. These covered the basic steps for beginner classes of ballroom and Latin American dance<br>Duration of intervention: 10 weeks	Usual care	Assessment of outcomes were performed within 14 days prior to and after the 10-week intervention period.<br>1. Whole body coordination during turning - 12 on-the-spot turns at a self-selected pace<br>2. Movement of eyes during turning - VNG Ulmer<br>3. Centre of pressure before and during the turn - Kistler force plate.<br>4. Number of turning steps, turn time, type and quality - Standing Start 180' Turn Test
Kunkel 2017	RCT, UK, n=51	Inclusion criteria:<br>Confirmed diagnosis of Parkinson's disease, Hoehn and Yahr scale of 1–3 indicating mild to moderate disease severity, lived at home, could understand and follow commands and had previous falls recorded.<br>Exclusion criteria: not stated.	Intervention group= Three ballroom (Social Foxtrot, Waltz, Tango) and 3 Latin American (Rumba, Cha Cha, Rock 'n' Roll) dances were taught by professional teachers in a dance centre. The classes lasted one hour, twice a week, for 10 weeks and PwP danced with their spouse, a friend or a volunteer.	"Control group participants were encouraged to continue with usual care, which typically comprised medication, attending medical clinics and routine visits from Parkinson's nurses."	Assessment of outcomes at 3 months and 6 months<br>1. Berg Balance Scale (higher score indicates better balance).<br>2. Activity-specific balance confidence (higher score indicates higher confidence).<br>3. Timed Up and Go Test<br>4. 6-minute walk test<br>5. Quality of life (PDQ-39: higher score indicates worse quality of life)<br>6. Feasibility<br>- a. recruitment<br>- b. retention<br>- c. outcome measures

Study ID (first author and year)	Design, country and total participants	Population and setting	Intervention	Comparison	Outcome
					- d. dance selection
Lee 2018	Randomized, blinded, waiting-list controlled partial crossover trial, Rep of Korea, n=32	Diagnosis: PD<br>N: 32<br>Age: mean 65.7 (SD 6.8) years<br>Sex: 58.5% female<br>Inclusion criteria:<br>1. age between 50 and 80 years<br>2. stage 1 to 3 on the Hoehn and Yahr scales3. no other neurological or cognitive impairments (K-MMES > 20)<br>4. not having received any exercise therapy within the 3 months prior to the study<br>Exclusion criteria: Not reported	Duration of intervention: 8 weeks<br>Timing of intervention: 60-minute sessions twice a week<br>Intervention group 1: Turo PD group	Control group 1: Wait-list control	1. Motor function: UPDRS<br>2. Perceived health status: PDQL<br>3. Depression: Beck Depression Inventory (BDI)<br>4. Balance: Berg Balance Scale (BBS)
McKee 2013	Controlled trial (unclear sequential allocation), US, n= 31	Diagnosis: idiopathic "definite PD"<br>N: 33<br>Inclusion criteria:<br>1. Hoehn and Yahr (H&Y) stages I–III)<br>2. No history of other neurological insult<br>3. 50 years or older<br>4. Could walk 3+ meters with or without assistance.	Tango Classes<br>90 minutes sessions with a total of 20 sessions over a 12-week period	Education<br>90 minute sessions with a total of 20 sessions over a 12 week period	1. Cognitive measures: MoCA, Reverse Corsi Blocks, and Brooks Spatial Task<br>2. Disease severity, mobility and balance: UPDRS-III, Fullerton Advanced Balance Scale, Four-Square Step Test, Single/Dual timed up and go with single task (TUG), dual-cognitive (Cognitive-TUG: counting backward by 3s from a randomly generated number between 20 and 100) and dual-manual (Manual-TUG: carrying full cup of water) conditions and everyday fall incidence outside of class.
McNeely 2015	Controlled trial (basis of allocation unclear), US, n=19	Diagnosis: idiopathic "definite" PD.<br>Other inclusion criteria:<br>1. Clear benefit from levodopa<br>2. Able to stand independently for at least 30 min<br>3. No evidence of dementia (MMSE ≥ 26)<br>4. No serious medical problem (aside from PD)<br>5. No evidence of abnormality on brain imaging (previously done for clinical evaluations – not part of this research)<br>6. No history or evidence of other neurological deficit, such as previous stroke or muscle disease<br>7. No deep brain stimulation<br>8. No other recent surgeries or injuries affecting movement.<br>Exclusion criteria: as above.	Dance for Parkinson's Disease (D4PD) Classes<br>-Participants participated in 12 weeks of dance, meeting two times per week for 1 hour each session.	Tango dance lessons	Outcomes assessment were performed before and after the 12-week dance intervention<br>1. Motor Sign Severity & Quality of life - MDS-UPDRS-III (higher score indicates more severe disease), Mini mental status exam (MMSE)(higher score indicates higher cognitive function) & PDQ-39 (higher score indicates worse quality of life).<br>2. Balance - Mini-BESTest (higher score indicates better balance).<br>3. Mobility<br>1) Time up and go (TUG)<br>2) 6-min walk test<br>3) four square step test<br>4) five times sit-to-stand test<br>4. Gait -<br>1) forward preferred speed<br>2) forward as fast as possible<br>3) backward<br>4) dual task walking

Study ID (first author and year)	Design, country and total participants	Population and setting	Intervention	Comparison	Outcome
Michels 2018	RCT, US, n=13	Diagnosis: Idiopathic PD<br>N: 13<br>Age: Mean 69.2 (SD 8.7) years<br>Sex: 6 male and 7 female participants<br>Inclusion criteria:<br>1. Any Hoehn & Yahr (H&Y) stage or disease severity<br>2. On a stable PD medication regimen for at least one month prior to the study and continue that regimen without any changes throughout the course of the study.<br>Exclusion criteria:<br>1. Participated in any therapeutic dance intervention within the three months before the start of the study<br>2. Initiated any new PD treatments<br>3. Involvement in other PD-focused interventions throughout the course of the study<br>4. Significant cognitive impairment determined by a Montreal Cognitive Assessment score (MoCA) less than 24<br>5. Under the age of 18	Duration of intervention : 10 weeks<br>Timing of intervention: 60 minutes weekly<br>Intervention group 1: Dance/movement Therapy (DT)	Control group 1: Support group control	1. Motor outcomes: UPDRS MDS, Berg Balance Scale (BBS), and Timed Up and GO (TUG)<br>2. Non-motor outcomes: MOCA, PDQ39, Beck Depression Inventory (BDI), Fatigue Severity Scale (FSS), Visual Analog Fatigue Scale
Poier 2019	RCT, Germany, n=29	Participants consisted of "Individuals diagnosed with PD (aged between 50 and 90 years and with signed informed consent)."	Argentinian Tango – one hour per week for 10 weeks	Tai-Chi – one hour per week for 10 weeks	1. Quality of life: 39-item Parkinson's Disease Questionnaire (PDQ-39)<br>2. The 10-item Brief Multidimensional Life Satisfaction Scale (BMLSS).
Rawson 2019	Controlled trial (alternate allocation into three arms), US, n=119	Inclusion criteria:<br>1. 30 years and older<br>2. clear benefit from levodopa<br>3. Hoehn & Yahr stages I to IV<br>4. ability to walk independently with or without an assistive device for at least 10 ft<br>5. no history of vestibular disease or dementia<br>6. diagnosis of "clinically definite PD."<br>Exclusion criteria:<br>1. Medical condition for which exercise is contradicted<br>2. evidence of abnormality other than PD-related changes on brain imaging<br>3. history or evidence of neurological deficit other than PD such as stroke or muscle disease, or orthopedic or muscular problem	Intervention 1: Tango<br>Participants practiced Argentine tango using an adapted curriculum for persons with PD.<br><br>Intervention 2: Treadmill<br>Treadmills were arranged in groups of 4 (2 pairs facing each other) to allow for social interactions.<br><br>Duration of intervention: twice per week for 12 weeks	Stretching<br>This active control group focused on gentle stretching and whole-body flexibility exercises designed for people with PD..	Outcomes assessments were performed at baseline, after the 12-week intervention (posttest), and follow-up (12 weeks after posttest).<br>1. Dynamic balance - Mini-Balance Evaluation Systems Test (Mini- BESTest)(higher score indicates better balance).<br>2. Motor function - MDS-UPDRS-III scores with Hoehn & Yahr staging (higher score indicates more severe disease).<br>3. Quality of life - Parkinson Disease Questionnaire-39 (PDQ-39) (higher score indicates worse quality of life).<br>4. Gait<br>1) 5-m GAITRite walkway (CIR Systems Inc, Franklin, New Jersey).<br>2) forward (FWD)<br>3) backward (BKD)<br>4) 6-minute walk test (SMWT)

Study ID (first author and year)	Design, country and total participants	Population and setting	Intervention	Comparison	Outcome
Rios 2015	RCT, Canada, n=33	Diagnosis: Idiopathic PD with Hoehn and Yahr stage I—III.<br>Other inclusion criteria:<br>1. Spoke either English or French sufficiently to fill out questionnaires and understand the instructions for dance classes<br>Exclusion criteria:<br>1. Could not stand for at least 30 min or walk for ≥3 m without an assistive device<br>2. Dementia (defined according to MDS dementia criteria)<br>3. Severe hearing and vision problems<br>4. Change in dopaminergic therapy over the preceding three months<br>5. Serious medical conditions which precluded dancing or could be worsened by exercise<br>6. More than 3 falls in the 12 preceding months (to ensure safety of intervention)<br>7. Other medical conditions which could affect study participation (e.g. drug abuse/alcoholism).	Tango<br>- 1-hour ‘‘traditional Argentine tango’’ classes twice a week for 12 weeks	Usual care with instructions to exercise.<br>- The control group was a wait-list group of patients with PD.<br>- Control participants followed their usual schedule of pharmacological treatment. In addition, they were provided a pamphlet about exercise in PD	Outcomes assessment were performed at the end of the intervention, 3 months after its commencement.<br>Primary Outcome<br>1. Motor function - MDS-UPRDS-3 (higher score indicates more severe disease)<br>Secondary Outcomes<br>1) MDS-UPDRS IV (fluctuation and dyskinesia) (higher score indicates more severe disease).<br>2. Balance - Mini-Balance Evaluation Systems Test (Mini-BESTest) (higher score indicates better balance).<br>3. Gait & balance - Timed Up and Go and Dual-task Timed Up and Go<br>4. Fall<br>5. Freezing gait - Freezing of Gait Questionnaire (FOG Q) (higher score indicates more severe condition).<br>6. Upper extremity function - Purdue Pegboard<br>2) Cognitive/Mood outcomes<br>1. Cognitive function - Montreal Cognitive Assessment (MoCA) (higher score indicates better cognitive function).<br>2. Depression - Beck Depression Inventory (BDI) (higher score indicates more severe depressive symptoms).<br>3. Apathy - Apathy Scale (AS) (higher score indicates less apathy).<br>3.Fatigue - Krupp Fatigue Severity Scale (higher score indicates less fatigue severity).<br>4. Quality of life - Parkinson’s Disease Questionnaire-39 (PDQ-39) (higher score indicates worse quality of life).<br>5. Clinical impression of change<br>6. Level of enjoyment
Rocha 2018	RCT, Australia, n=21	Diagnosis: Idiopathic PD<br>N: 21<br>Age:<br>1. Argentine tango group: mean 70.2 (SD5.5) years<br>2. Mixed dance group: mean 72.9 (SD5.5) years<br>Sex:<br>1. Argentine tango group: male 4 female 6<br>2. Mixed dance group: male 4 female 7<br>Inclusion criteria:<br>1. Rated I–IV on the modified Hoehn and Yahr scale	Duration of intervention: 8 weeks<br>Timing of intervention: 60-minute, in-person community dancing class once a week<br>Intervention group 1: Artegntine tango group<br>Co-intervention for both groups: Once-weekly, 40-minute, self-managed home dance programme.	Control group 1: Mixed dance group	1. Time Up and Go Test<br>2. Berg Balance Scale<br>3. Functional Gait Assessment<br>4. Freezing of Gait questionnaire<br>5. Movement Disorders Society Unified Parkinson’s Disease Rating Scale sections II and III<br>6. 39-item Parkinson’s Disease Questionnaire.

Study ID (first author and year)	Design, country and total participants	Population and setting	Intervention	Comparison	Outcome
		2. able to stand for at least 2 minutes<br>3. able to walk independently for more than 3 metres with or without assistive devices<br>Exclusion criteria:<br>1. Could not understand spoken English<br>2. Had scores on the Mini-Mental State Examination (MMSE) lower than 24 out of 30<br>3. Had any comorbidities that prevented exercise<br>4. Were unable to travel to the dance venue<br>5. Had previously received deep-brain stimulation surgery			
Shanahan 2017	RCT, Ireland, n=90	Individuals with idiopathic Parkinson disease<br>Exclusion criteria: not stated in the paper.	Dance<br>- Weekly 1.5-hour class for 10 weeks<br>- Classes started with a warm-up, targeting movement speed and size, postural alignment, and other physiological systems required for dance.<br>- Exercises were progressed from sitting to standing according to abilities.	Usual care	Outcome assessment was performed at around 3 months.<br>Primary outcome:<br>1) Feasibility<br>1. Quantifying the success of randomization and allocation procedures.<br>2. Resources. -Availability and cost of buildings, dance studios, and personnel (researchers, assessors, independent mediator, dance partners, dance teachers, and health practitioners) were documented.<br>3. Success of recruitment - If 100 participants could be recruited in 1 year. T<br>4. Willingness of participants to be randomized<br>5. Attrition rates for the entire study<br>6. Safety<br>7. Adherence to the Irish set dancing intervention<br><br>Secondary outcomes<br>1. Motor function - Unified Parkinson's Disease Rating Scale (higher score indicates more severe disease).<br>2. Functional endurance - 6-minute walk test<br>3. Balance - mini-BESTest (higher score indicates better balance).<br>4. Quality of life- Parkinson's Disease Questionnaire-39 (PDQ-39) (higher score indicates worse quality of life).
Solla 2019	RCT, Italy, n=20	Clinical diagnosis of PD according to Gelb's criteria, a score ≤ 3 on the Hoehn and Yahr (H&Y) scale, ability to walk without walking aids, stable medication regimen in the 4 weeks before the study and a score ≥ 24 on the Mini-Mental State Examination.	Sardinian folk dance<br>- 90-min class sessions, performed twice per week for 12 weeks.	Usual medical care	1. Unified Parkinson's Disease Rating Scale Part-III (UDPRS-III).<br>2. Functional performance: 6-minute walking test (6MWT), Five Times Sit-To-Stand Test (FTSST), Timed Up-and-Go (TUG) test

Study ID (first author and year)	Design, country and total participants	Population and setting	Intervention	Comparison	Outcome
					3. Berg Balance Scale (BBS) to evaluate static balance.<br>4. Mobility: Sit-and-Reach Test (SRT), and the Back Scratch Test (BST).
Volpe 2013	RCT, Italy, n=24	Diagnosis: Idiopathic Parkinson's disease as diagnosed by a medical practitioner and were rated level 0–2.5 on the modified Hoehn & Yahr scale.<br>Other inclusion criteria:<br>1.Mild to moderately severe PD for safety reasons<br>2. Scored more than 24/30 on the MMSE<br>Exclusion criteria:<br>1. did not speak Italian<br>2. co-morbidities that prevented dancing, mobility or safe exercise<br>3. received deep brain stimulation surgery<br>4. unable to travel to the dancing or physiotherapy venues)	Irish set dancing<br>- 90-minute set dancing class weekly for six months	Physiotherapy exercise	Outcomes assessments performed at baseline (3 weeks prior to therapy), within 3 weeks of the final week of the six-month therapy period, and 3 weeks after discharge.<br>Primary outcome:<br>1) Feasibility<br><br>Secondary outcomes:<br>1) Motor function – MDS-UPDRS subscale 3 (higher score indicates more severe disease)<br>2) Agility - Timed Up and Go (seconds)<br>3) Berg Balance Scale (higher score indicates better balance).<br>4) Freezing of Gait Questionnaire (higher score indicates more severe condition).<br>5) Quality of life - PDQ-39 (higher score indicates worse quality of life).
